# Discovery of a novel cannabidiol-derived transient receptor potential vanilloid 4 inhibitor to reduce pulmonary edema and lung vascular permeability in mice

**DOI:** 10.1186/s11658-026-00939-3

**Published:** 2026-05-17

**Authors:** Yassir Arfath, Pankaj Singh Cham, Tusharika Kotra, Rahila Akhter, Sumeer Ahmed, Mandeep Kour, Sheikh Tasduq Abdullah, Parvinder Pal Singh, Sheikh Rayees, Zabeer Ahmed

**Affiliations:** 1 Pharmacology Division, CSIR-Indian Institute of Integrative Medicine, Canal Road, Jammu, 180001 India; 2 Academy of Scientific and Innovative Research (AcSIR), Ghaziabad, India; 3 NPMC Division, CSIR-Indian Institute of Integrative Medicine, Canal Road, Jammu, 180001 India

**Keywords:** TRPV4, Inflammation, ALI, Ca^2+^, Cannabidiol, Edema, Vascular permeability, NLRP3

## Abstract

**Introduction:**

Activation of TRPV4 ion channel during acute lung injury (ALI) exacerbates lung dysfunction by promoting edema and inflammation. Pharmacological inhibition of TRPV4 signaling in the lungs offers protective benefits, reducing vascular leakage, enhancing blood oxygenation, and alleviating edema.

**Objectives:**

We designed, synthesized, and preclinically evaluated cannabidiol-derived TRPV4 channel inhibitors for potential therapeutic application in ALI and future clinical translation.

**Methods:**

We identified a lead cannabidiol-derived TRPV4 inhibitor through specific* in vitro* screening assays. The lead compound was then tested in a series of animal models of ALI. Initial evaluation employed the lipopolysaccharide (LPS) induced lung injury model, followed by models involving TRPV4 overexpression in alveolar macrophages, as well as models featuring TRPV4 hyperactivation. These models were strategically chosen to replicate key pathological features of clinical ALI.

**Results:**

Our investigation revealed that administration of the lead derivative CS-85(**4j**) demonstrated significant protective effects in a mouse model of ALI. CS-85 effectively prevented lung edema and maintained the integrity of pulmonary vascular barrier. Notably, it inhibited neutrophil influx into the lung, reduced proinflammatory cytokine production, and mitigated associated pathological changes. In additional relevant preclinical *in vivo* models, we further investigated how TRPV4 hyperactivation via pharmacological stimulation and overexpression in alveolar macrophages through liposome-mediated gene delivery exacerbated key features of ALI. CS-85 effectively reduced this exaggerated lung inflammation and alleviated the ALI features. In exploring the downstream mechanisms of CS-85, we found that its pharmacological efficacy is mediated through modulation of the NLRP3-caspase-1, NFAT, and NF-ĸB signaling pathways, all of which are crucial inflammatory cascades.

**Conclusions:**

We identified CS-85 as a potent and promising TRPV4 inhibitor that demonstrates strong preclinical efficacy in mitigating ALI by preserving vascular integrity and modulating key inflammatory signaling pathways. Its dual mechanism of action highlights its therapeutic potential for ALI and supports further clinical evaluation.

**Supplementary Information:**

The online version contains supplementary material available at 10.1186/s11658-026-00939-3.

## Background

Acute lung injury is a severe inflammatory condition characterized by arterial hypoxemia, lung edema, disruption of epithelial–endothelial capillary barrier integrity, and infiltration of immune cells into the lung [1]. Prompt suppression of this inflammatory condition is critical for reinstatement of tissue homeostasis. Failure to do so can lead to acute respiratory distress syndrome (ARDS), the most severe form of acute lung injury (ALI), which often results in respiratory failure. Currently, no specific single drug is available for ARDS, and treatment is primarily supportive [2, 3]. ALI/ARDS has been widely recognized as a significant healthcare challenge owing to its high morbidity and mortality rates [4, 5]. Approximately 3 million cases of ARDS are reported each year worldwide, and the mortality rate ranges from 35% to 46% [6, 7]. Treatment for ALI mainly involves supportive care, aiming to resolve pulmonary edema and restore endothelial and epithelial barrier integrity. This includes reduced tidal volume, fluid-conservative therapy, and anti-inflammatory drugs [8].

Macrophages and neutrophils constitute the majority of immune cells that infiltrate the lung during ALI. Macrophage activation substantially contributes to the pathogenesis of ALI by releasing proteases, cytokines, and reactive oxygen species (ROS) [9, 10]. Alveolar macrophages (AMs), the sentinel immune cells of the lung, are the primary responders that sense foreign particles, orchestrate respiratory immune responses, and initiate the inflammatory cascade that leads to ALI. Transient receptor potential vanilloid 4 (TRPV4) ion channel plays a pivotal role in mediating the proinflammatory response of AMs, and its activation plays a substantial role in lung injury. TRPV4 is a calcium-permeable, mechanosensitive cation channel in the TRPV subfamily of transient receptor potential (TRP) channels [11]. It is highly expressed in lung cells such as epithelium, alveolar-capillary structures, major pulmonary arteries, and immune cells such as AMs and neutrophils [12, 13]. TRPV4 plays a substantial role in regulating lung injury [14]. Abrogation of TRPV4 mediated signaling in the lungs by pharmacological intervention exerts a vasculoprotective effect by inhibiting vascular leakage, improving blood oxygenation, and resolving edema [10, 15, 16]. Activation of TRPV4 by selective pharmacological agonists or through other mechanisms augments lung dysfunction in ALI [17, 18, 19]. The gating behavior of this channel is influenced by both exogenous and endogenous factors, which contribute to its diverse physiological and pathophysiological role. They include heat [20], mechanical stress [21], osmolarity [22], pH [23], and UV radiation [24]. Plant-derived compounds, known for their high specificity and potency as ligands for the TRPV4 ion channel, effectively modulate TRPV4 activity. Some of the plant-based TRPV4 ligands include bisandrograholide-A [25], apigenin [26], phorbol-12-myristate-13-acetate, 4α-phorbol 12,13-didecanoate [27], cannabidiol [28], and ginkgetin [29]. Cannabidiol, the major non-psychoactive component of cannabis, exhibits a wide range of pharmacological effects through its interaction with TRP channels [30].

Over the past two decades, significant progress has been made in developing specific TRPV4 channel inhibitors. However, clinical advancement has been largely impeded by several challenges. Here, we synthesized a targeted series of cannabidiol (CBD-**1**) derivatives and identified CS-85 as the most effective TRPV4 inhibitor of this series. In a mouse model of ALI, administration of CS-85 prevented protein-rich lung edema, reduced cytokine production, maintained vascular endothelial–epithelial integrity, and blocked neutrophil influx to the lung. The effectiveness of CS-85 emanates from interaction with TRPV4, NLRP3-caspase-1, and NF-kB pathways, which are central to several inflammatory cascades.

### Material and methods

#### Plant

Authentic aerial parts of *Cannabis sativa* were collected from a designated and licensed cultivation site (permit no. 559 of 2017, dated 31 March 2017) at Chatha Farm, Jammu, J&K, India. The plant material was taxonomically identified, and a voucher specimen (no. 25119, dated 15 June 2021) was deposited in the Janaki Ammal Herbarium at the Council of Scientific and Industrial Research–Indian Institute of Integrative Medicine (CSIR–IIIM), Jammu, India.

#### Cell lines

A375 cells were kindly gifted by Dr. Sheikh Tasduq Abdullah, senior principal scientist at CSIR–IIIM. RAW 264.7 cell line was purchased from NCCS (Pune, India). HCT 116 cells were kindly gifted by Dr. Shashank K. Singh, chief scientist at CSIR–IIIM, and bone marrow-derived macrophages (BMDMS) were isolated from the bone marrow of mice.

#### Reagents and enzyme-linked immunosorbent assay (ELISA) kits

The following reagents and ELISA kits were used in this study: Sigma Aldrich, USA: 2′,7′-dichlorofluorescein diacetate (DCFH-DA), D6883; lipopolysaccharide, L2880; Griess reagent, G4410; RIPA lysis buffer, 20188; Evans blue, E2129; GSK1016790A, 530533; GSK2193874, SML0942; adenosine triphosphate (ATP), A6419-5G; capsaicin, M2028; and carvacrol, 42632. Thermo Fisher Scientific, USA: i-NOS primary antibody (rabbit), 53592082; fetal bovine serum, 10270106; calcium-containing HBSS, 14025,076; calcium-free HBSS, 14175095; and mouse IL-1β ELISA kit, 887013A88. R&D Systems, USA: mouse IL-6 ELISA kit, DY406; mouse IL-1β ELISA kit, DY401; and IL-1β primary antibody, AF-401NA. Cell Signaling Technology, USA: phospho-NF-κB p65, 3033S; anti-rabbit IgG, 7074P2; anti-mouse IgG,7076; NFAT1, 5861T; caspase-1, 24232S; caspase-1, 89332S; NLRP3, 15101; and β-actin, 4970S. Cayman Chemicals, USA: 4α-phorbol 12,13-didecanoate, 20446, and probenecid, 26294.

#### Experimental animals

The animal facility of CSIR–IIIM, Jammu, India, is a state-of-the-art Good Laboratory Practice (GLP) standard pathogen-free housing facility. C57BL/6 mice (both genders, 6–8 weeks old, and ~30 g) used in the study were bred and maintained at this facility. All mouse experiments were performed as per the guidelines of the Committee for Control and Supervision of Experiments on Animals (CCSEA) in line with International Council for Laboratory Animal Science (ICLAS) standards, with the approval of the Institutional Animal Ethics Committee (IAEC) (IAEC approval nos. 333/82/2/2023 and 360/84/2/2024). A total of 345 mice were used in the study.

#### Bone marrow-derived macrophage extraction

BMDMs were harvested from mice as described [31, 32]. Briefly, following euthanasia, the femur and tibia of mice were harvested and flushed with complete Roswell Park Memorial Institute (RPMI) medium containing 30 ng/mL macrophage colony-stimulating factor (M-CSF). The cell suspension was filtered via a 70-μm cell strainer to remove debris. The cells were then cultured at 37 °C and 5% CO_2_. On day 3, the exhausted medium was removed and fresh complete RPMI (M-CSF-free) was added and the cells kept for an additional 2–3 days [31].

#### Measurement of calcium dynamics in the cytoplasm

We evaluated the influx of calcium following pretreatment with test compounds and subsequent exposure to respective ion channel agonists in a fluorescence-based assay, by determining 340/380 nm ratio of Fura-2 AM, on a plate reader (Tecan Infinite M200 PRO). To measure cytosolic Ca^2+^ influx, cells were cultured in black 96-well plates. Intracellular Ca^2+^ changes were evaluated using Fura-2 AM dye (3 µM) as described [31]. In brief, the cells were loaded with Fura-2 AM in incomplete Dulbecco’s modified eagle medium (DMEM) for 40 min at room temperature (RT) in the dark, followed by a gentle wash with calcium-free HBSS. The cells were exposed to test compounds in calcium-containing HBSS for 15 min and then stimulated by respective channel agonists for the indicated time. The fluorescence was recorded at 340/510 nm and 380/510 nm, for calcium-bound and calcium-free dye, respectively. The data are presented as a ratio of 340/380 to quantify changes in intracellular calcium levels.

#### Cell viability assay

We performed a 3-(4,5-dimethylthiazol-2-yl)-2,5-diphenyltetrazolium bromide (MTT) assay to evaluate cytotoxicity of test compounds. The cells were cultured at a density of 1 × 10^5^ cells per mL (100 µL per well) and incubated at 37 °C with 5% CO_2_ in a cell culture incubator overnight. On the following day, the cells were treated with test compounds at multiple concentrations and further incubated for 24 h. The cells in each well were then incubated with 20 μL of MTT dye (2.5 mg/mL phosphate-buffered saline [PBS]) for 4 h at 37 °C before termination. After 4 h, the media was discarded completely, formazan crystals were dissolved in 100 μL of dimethylsulfoxide (DMSO), and optical density was recorded using a spectrophotometer (Tecan Infinite M200 PRO) at 570 nm. The percentage of cell viability was calculated by using the formula:

Percentage (%) cell viability = absorbance of treated cells/absorbance of untreated cells × 100.

#### Nitric oxide quantification

Nitric oxide is a commonly used and reliable readout of lipopolysaccharide (LPS) induced TLR4 activation in macrophages. The release of nitric oxide from LPS-stimulated and CS-85-pretreated cells was determined using Griess reagent. For the nitric oxide quantification, the cells were cultured in a 96-well plate at a density of 5 × 10^5^ cells per mL (100 µL per well) in a 96-well flat-bottom plate. The cells were treated with the test compound, L-NAME (100 μM) for 1 h followed by LPS (1 μg/mL) stimulation for 24 h. The supernatant was harvested, and nitric oxide levels were quantified. This assay was performed by mixing Griess reagent and cell culture supernatant in a 1:1 ratio at room temperature for 5 min. In addition, optical density was measured at 540 nm using a plate reader (Tecan Infinite M200 PRO). LPS-stimulated cells served as a positive control. The final estimation was based on the sodium nitrite curve.

#### Estimation of intracellular reactive oxygen species (ROS)

The release of ROS was measured using the fluorogenic probe DCFH-DA. BMDMs and RAW 264.7 cells (2 × 10^5^ per mL, 100 µL/well) were plated in 96-well, non-optical-bottom black plates and incubated at 37 °C with 5% CO_2_ in a cell culture incubator overnight. On the day of experiment, the cells were pretreated with indicated concentrations of CS-85 for 1 h followed by LPS (1 µg/mL) exposure for another 24 h. The cells were then washed with PBS, and 5 µM DCFH-DA dye was loaded in the phenol-free RPMI and incubated for 30 min at 37 °C in the dark. Post incubation, the cells were again washed twice with PBS, and the fluorescence was recorded using a spectrofluorometer (Tecan Infinite M200 PRO) at 493 nm and 527 nm excitation and emission, respectively.

#### Measurement of cytokine secretion using ELISA

The cell culture supernatants were collected, briefly centrifuged, and used to estimate IL-1β and IL-6 using ELISA. Similarly, in animal experiments, bronchoalveolar lavage fluid was subjected to brief centrifugation and quantified for IL-6 and IL-1β levels using ELISA. The ELISA assays were performed according to manufacturer’s protocols (R&D Systems and Thermo Fisher Scientific). Absorbance was measured at 450 nm, and cytokine concentrations were determined by comparison with the respective standard curve.

#### NLRP3 inflammasome activation

We conducted an NLRP3 inflammasome activation assay to evaluate the inhibitory potential of CS-85 against NLRP3 inflammasome complex formation and activation. We used LPS for priming followed by ATP stimulation to specifically activate NLRP3 inflammasome assembly. The IL-1β collected from cell supernatant was quantified by ELISA as readout. The cells were seeded in 24-well plates for ELISA and 60-mm dishes for western blotting. Following priming with LPS (1 μg/mL) for 3.5 h, the cells were washed with serum-free media to remove any residual serum particles. ATP (5 mM) was then applied as the activation signal for NLRP3 inflammasome in the LPS-primed cells. Treatment of CS-85 was carried out for 1 h prior to ATP exposure. CS-85 and ATP treatment for NLRP3 activation was carried out in incomplete media. ATP was dissolved in freshly prepared chilled 1N NaOH and distilled water and was added for 30 min before the termination of the experiment. MCC950 (100 nM) was used as a reference standard.

#### Confocal imaging

Live-cell confocal imaging was performed on A375 cells to monitor Ca^2+^ dynamics under indicated treatment conditions. Intracellular Ca^2+^ influx was assessed using Fluo-3 AM, a fluorescent calcium dye. The cells were imaged using a laser-scanning confocal microscope with optimized acquisition settings optimized for the Fluo-3 AM (excitation/emission: 506/526 nm). The cells were seeded at a density of 2 × 10^5^/mL (1.5 mL per dish) in a 35-mm glass-bottom petriplate and incubated over night. The cells were then loaded with Fluo-3 AM for 40 min at room temperature and rinsed with calcium-free HBSS. The cells were treated with CS-85 for 15 min in calcium-containing HBSS. Following this, GSK1016790A (GSK-A) was applied to the cells, which were then incubated for a further 15 min. Images were acquired on a confocal microscope (CQ1 confocal imaging cytometer, Yokogawa, Japan). A quantitative analysis of images was performed by Image J software.

#### Induction of lung injury

Animals were anesthetized by intraperitoneal injection of a ketamine (80 mg/kg) and xylazine (15 mg/kg) cocktail. ALI was induced by noninvasive endotracheal instillation of LPS (75 μg/mouse). Following 24 h of LPS instillation, the mice were euthanized, thoracotomy was performed, and lungs were perfused with phosphate-buffered saline. The lung lobes were collected.

Pulmonary edema was assessed by calculating the lung wet-to-dry weight ratio. To do that, the left lung lobe was excised, immediately weighed (wet weight), and then dried at 55 °C for 48 h to obtain the dry weight. The wet-to-dry ratio was calculated as an indicator of edema, as described previously [33]. To assess lung vascular permeability/leakiness in the above mentioned experimental mice, Evans blue (20mM) -conjugated albumin (EBA) was administered retro-orbitally (100 µL/mouse) 30 min prior to thoracotomy/sacrifice, as previously described [33, 34]. Blood was collected from the right ventricle into heparinized tubes, and plasma was separated by centrifugation. Right lung lobes were harvested, homogenized, and lysates were prepared. The lung lysates and plasma samples were incubated with a 2:1 formamide:PBS solution at 55 °C for 48 h, followed by centrifugation at 5000 rpm for 30 min. The absorbance of the supernatant was measured using a spectrophotometer (Tecan Infinite M200 PRO) at 620 nm for Evans blue and 740 nm for hemoglobin correction. EBA extravasation was quantified as the ratio of EBA concentration in lung tissue to that in plasma.

#### Bronchoalveolar lavage

ALI was induced in mice by LPS as described above. Then, 24 h after induction of lung injury, each mouse was anesthetized using ketamine/xylaxine, and bronchoalveolar lavage was performed as described previously [33, 35]. Briefly, a tracheotomy was carried out, and BAL was performed with a 20-gauge blunt needle. Cold PBS (1 mL) was gently instilled into the lungs and then aspirated back. Approximately 1 mL of BAL fluid was collected and briefly centrifuged, and the supernatant was used for further experiments.

#### Histopathology and myeloperoxidase assay

The mice were pretreated with CS-85, and ALI was induced by LPS as indicated above. The experiment was terminated 24 h after LPS instillation. The lungs were then perfused with PBS, collected, and prepared for hematoxylin and eosin (H&E) staining and MPO assay. For H&E staining, lung lobes were fixed in formalin and then embedded in paraffin to create a formalin-embedded block for histology. After deparaffinization and rehydration, 4-μM sections of the lung tissues were stained with H&E according to standard procedures. The remaining lung lobes were harvested and homogenized in cold PBS containing proteinase inhibitor. The homogenate was centrifuged for 5 min at 5000 rpm and 4 °C, and the pellets were dissolved in lysis buffer (50 mM HEPES, pH 7.4, 50 mM NaCl, 1% Triton X-100, 5 mM EDTA, 1 mM DTT, 10 mM sodium pyrophosphate, 50 mM sodium fluoride, and 1 mM sodium orthovanadate) supplemented with fresh proteinase inhibitor cocktail. The homogenate was vigorously vortexed for 10–15 s on ice and then allowed to rest for 7 min at 4 °C. This procedure was repeated four times in total. The samples were centrifuged at 13,000 rpm for 10 min at 4 °C, after which the supernatants were collected and the pellets were discarded. Protein samples 50 µL (100–200 µg) were mixed with 950 μL reaction buffer (50 mM phosphate buffer containing 0.167 mg/mL of *o*-dianisidine dihydrochloride and 0.005% H_2_O_2_) for 30 min. The reaction was stopped by adding stop solution (2N H_2_SO_4_) for 5 min. Absorption was measured at 460 nm to estimate MPO activity.

#### Liposome-mediated gene delivery

Cationic liposomes were procured commercially (Sigma Aldrich, USA). Trpv4 plasmid DNA was quantified and gently mixed with liposomes. The liposome-loaded plasmid DNA (50 µg) was administered endotracheally into the lungs of the mice. Then, 24 h after liposome administration, the mice were pretreated with CS-85, and 1 h later, they were exposed to LPS (75 μg/mouse) as described earlier [35, 33]. The lungs were collected for assessment of lung edema and vascular permeability by calculating wet-to-dry ratio and EBA extravasation, respectively. For the confirmation of successful delivery of Trpv4 plasmid DNA into alveolar macrophages, we measured *Trpv4* expression in alveolar macrophages by real-time polymerase chain reaction (PCR). Toward this end, BAL was performed in *Trpv4*transduced and control mice 24 h after liposome administration, and alveolar macrophages were harvested. Additionally, we measured *Trpv4* expression in lung homogenate of lavaged lung to assess nonspecific delivery of Trpv4 plasmid DNA to other cell types.

#### Immunoblotting

The standard western blotting procedure was adopted for estimation of protein levels in both* in vitro* and mouse samples. Proteins were separated by sodium dodecyl sulfate polyacrylamide gel electrophoresis (SDS–PAGE) and transferred onto nitrocellulose membranes using a BioRad miniblot apparatus (Biorad, USA). Following protein transfer, the nitrocellulose membrane was blocked for 2 h in 5% fat-free milk or 3–5% BSA. Primary antibody incubation of membranes was carried out overnight at 4 °C with gentle shaking. After washing, the bound primary antibodies were detected using secondary antibodies. The membranes were scanned using a Chemidoc MP gel imager (Biorad, USA) or Syngene Gel documentation System (Cambridge, UK), and the blots were analyzed with Image J software.

#### Transfection

Raw 264.7 macrophages were transfected with green fluorescent-tagged expression plasmids of human *Trpv1* (hTRPV1), human *Trpv3* (hTRPV3), and human *Trpv4* (hTRPV4) using Lipofectamine 3000 (Thermo Fisher Scientific, USA) [36, 37], following the manufacturer’s instructions. Then, 100 ng of plasmid DNA in 0.4 μL of Lipofectamine 3000 was added to each well of a 96-well, black optical-bottom plate, in 50 μL of Opti-MEM media (Thermo Fisher Scientific, USA) and incubated for 6–7 h. The medium was then replaced with fresh, complete DMEM. The cells were used 24 h after transfection. The experimental controls were transfected with nontargeted (scrambled) DNA.

#### Alveolar macrophages depletion

Commerically available clodronate liposomes (Encapsula Nanosciences, USA) were administered endotracheally to the mice, as described in our previous study [33]. Then, 24 h after clodronate liposome instillation/AM depletion, the mice were treated with the indicated concentration of CS-85 and then exposed to endotoxin LPS for an additional 24 h, as shown in the outline. The mice were sacrificed 49 h after clodronate instillation, and bronchoalveolar lavage was collected and analyzed to quantify inflammatory cytokine levels.

#### Molecular modeling

The three-dimensional (3D) structure of the TRPV4 channel (PDB ID: 8J1F) was retrieved from the RCSB Protein Data Bank (www.rcsb.org) and prepared using Chimera’s DockPrep tool. All water molecules, ligands, and ions were removed, and missing hydrogen atoms and charges were added using AutoDock Tools (ADT) to generate the final protein structure in pdbqt format. Ligands were initially sketched using ChemDraw and saved in MOL format, then converted to PDB format using OpenBabel (version 2.4.1). Subsequently, Gasteiger–Marsili charges were applied, nonpolar hydrogens were merged, AutoDock atom types were assigned, and torsions were defined using ADT version 1.5.7, resulting in ligand pdbqt files. Potential binding pockets of TRPV4 were identified by combining results from LigPlot and the web-based tool PrankWeb, which guided the selection of critical amino acids and docking regions. A grid box with dimensions of 114.145 × 119.7581 × 148.3518 Å with 1 Å spacing was generated using AutoDock Tools version 4.2 for docking simulations. Structure-based and ligand-based virtual screening was conducted via rigid docking using AutoDock Tools version 1.5.7, and the resulting protein–ligand complexes were visualized and analyzed using PyMOL and Discovery Studio Visualizer.

#### Statistical analysis

Data are expressed as mean ± standard deviation (SD). Statistical analysis was carried out using Graph Pad Prism version 8.0. Differences among groups were evaluated by one-way analysis of variance (ANOVA) with Tukey’s test to assess significance.

## Results

### Chemistry

**Fig. 1:**
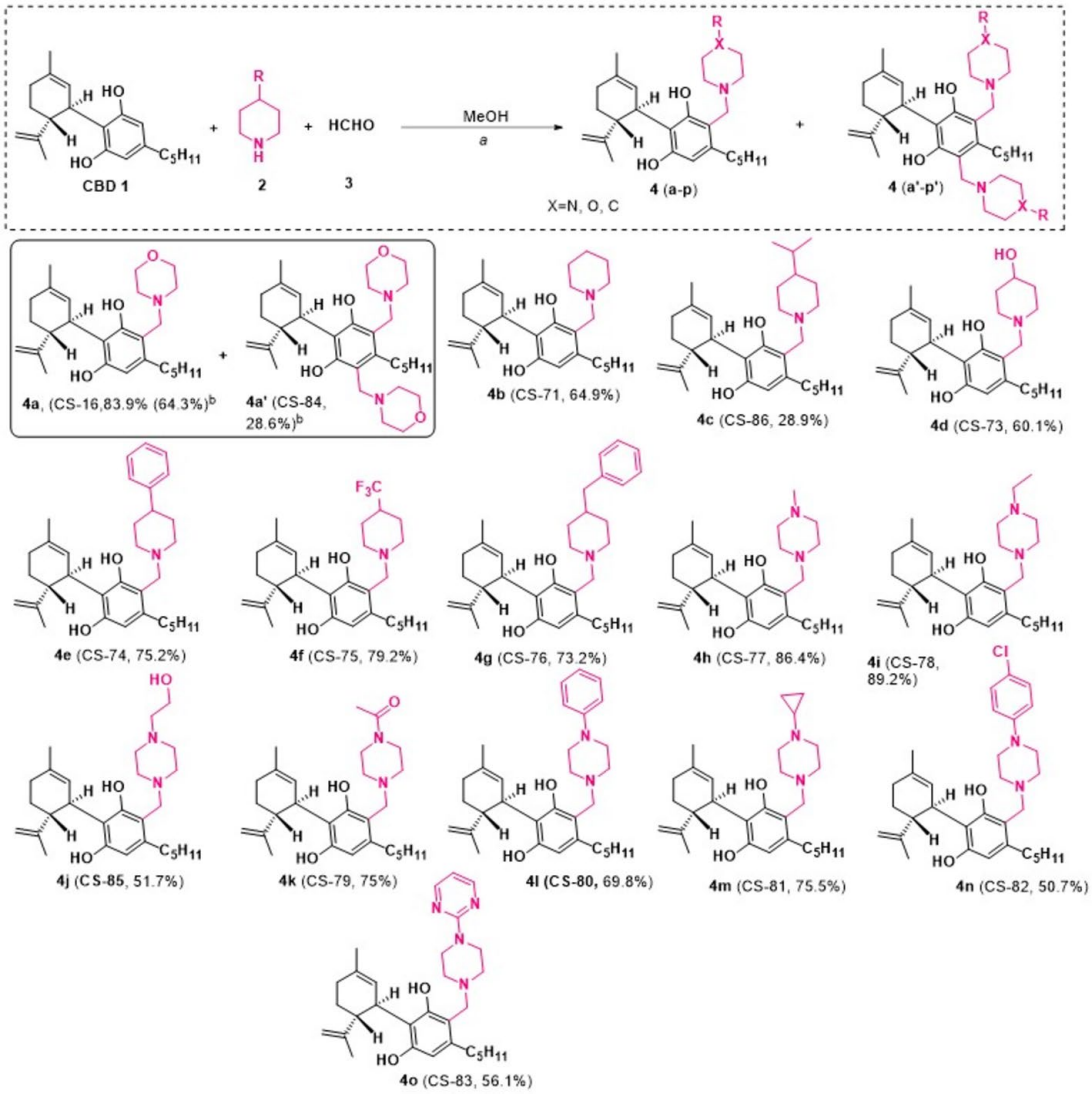
Synthesis of novel CBD-1 analogs. ^a^ Reagents and conditions: **1** (0.382 mmol, 120 mg); **2** (0.458–1.146 mmol, 1.2 eq); **3** (37%, 83 µL, 0.840 mmol, 2.2 eq); MeOH (4 mL), room temperature, under nitrogen atmosphere, 15–17 h; ^b^ Reaction was performed **3** equivalent of **2**, **4** equivalent of **3.**

Here, we focused on the synthesis of a novel series of heterocyclic-ring-containing cannabidiol analogs (Fig. 1). CBD-**1** was isolated from *Cannabis sativa* as reported in our previous studies (Supplementary Fig. S1) [38, 39, 40]. Subsequently, the novel CBD-**1** analogs were synthesized through the coupling of CBD-**1** with various secondary amines (**2**) in the presence of formaldehyde (HCHO), using methanol (MeOH) as the solvent (Fig. 1, Supplementary Figs. S2–S4, and Supplementary Tables S1 and S2). The coupling reactions involved CBD-**1** and a series of secondary amines, including piperidine (**2b**), 4-isopropylpiperidine (**2c**), piperidin-4-ol (**2d**), 4-phenylpiperidine (**2e**), 4-(trifluoromethyl)piperidine (**2f**), 4-benzylpiperidine (**2 g**), 1-methylpiperazine (**2 h**), 1-ethylpiperazine (**2i**), 2-(piperazin-1-yl)ethanol (**2j**), 1-(piperazin-1-yl)ethanone (**2 k**), 1-phenylpiperazine (**2 l**), 1-cyclopropylpiperazine (**2 m**), 1-(4-chlorophenyl)piperazine (**2n**), and 2-(piperazin-1-yl) pyrimidine (**2o**). This approach yielded the corresponding derivatives (**4a–o**) with yields of 83.9%, 64.9%, 28.9%, 60.1%, 75.2%, 79.2%, 73.2%, 86.4%, 89.2%, 51.7%, 75.0%, 69.8%, 75.5%, 50.7%, and 56.1%, respectively. The reaction of 1.2 equivalents (eq) of morpholine (**2a**) provided the monomorphonyl-CBD-**1** derivative (**4a**) as a single product (83.9%). Interestingly, increasing the amount of coupling reagents to 3 equivalents yielded compounds **4a** and **4a′** in 64.3% and 28.6%, respectively. All compounds were characterized using NMR and mass spectrometry, with detailed spectra provided in the Supplementary Information (SI) (Figs. S5–S68). Overall, 16 novel CBD-**1** analogs were synthesized (Fig. 1) and subjected to biological evaluation.

TRPV4 possesses two separate ligand-binding domains, an agonistic site that facilitates channel activation, and an antagonistic site that inhibits it [41, 42]. The reference molecule CBD-**1** preferentially binds to the agonistic pocket, leading to channel activation. Molecular docking studies were conducted to examine the binding behavior of the synthesized compounds at both identified TRPV4 binding sites. The results demonstrated distinct site-selective binding, with specific molecules occupying the antagonistic pocket, while others bonded at the agonistic site (Supplementary Fig. S69A–P and Supplementary Table S3). The compounds with oxygen-atom-containing hydrogen-bond donors or acceptors predominantly interacted with the antagonistic site. Among the active hits, compound **4j** exhibiting the most robust interaction (−6.75 kcal/mol) (Supplementary Fig. S69A, L), followed by **4 k** (−6.12 kcal/mol) and **4d** (−5.78 kcal/mol). The compounds **4i** (−4.13 kcal/mol) and **4a′** (−3.87 kcal/mol) exhibited moderate binding at the antagonistic site.

### Solubility studies

The effectiveness of CBD-**1** is limited by its low solubility. Therefore, active compounds **4a**, **4d**, **4j** (CS-85), and **4k** were evaluated for solubility in various biorelevant media such as water, simulated gastric fluid (SGF), and simulated intestinal fluid (SIF), along with CBD-**1** (Supplementary Table S4). Piperine was used as a standard, and results showed good concordance with previously reported values [43]. CBD-**1** demonstrated low solubility in both SGF and SIF. All candidates showed over 300-fold higher solubility in SGF and enhanced solubility in SIF with varying degrees of enhancement compared with the parent. Notably, **4j** (CS-85) showed over 300-fold increase in solubility in SGF and over 16-fold increase in SIF compared with CBD-**1** (Supplementary Table S4). For *in vitro* bioevaluation studies, the CBD-**1** analogues were solubilized in DMSO.

### Identification of TRPV4 inhibitors

**Fig. 2:**
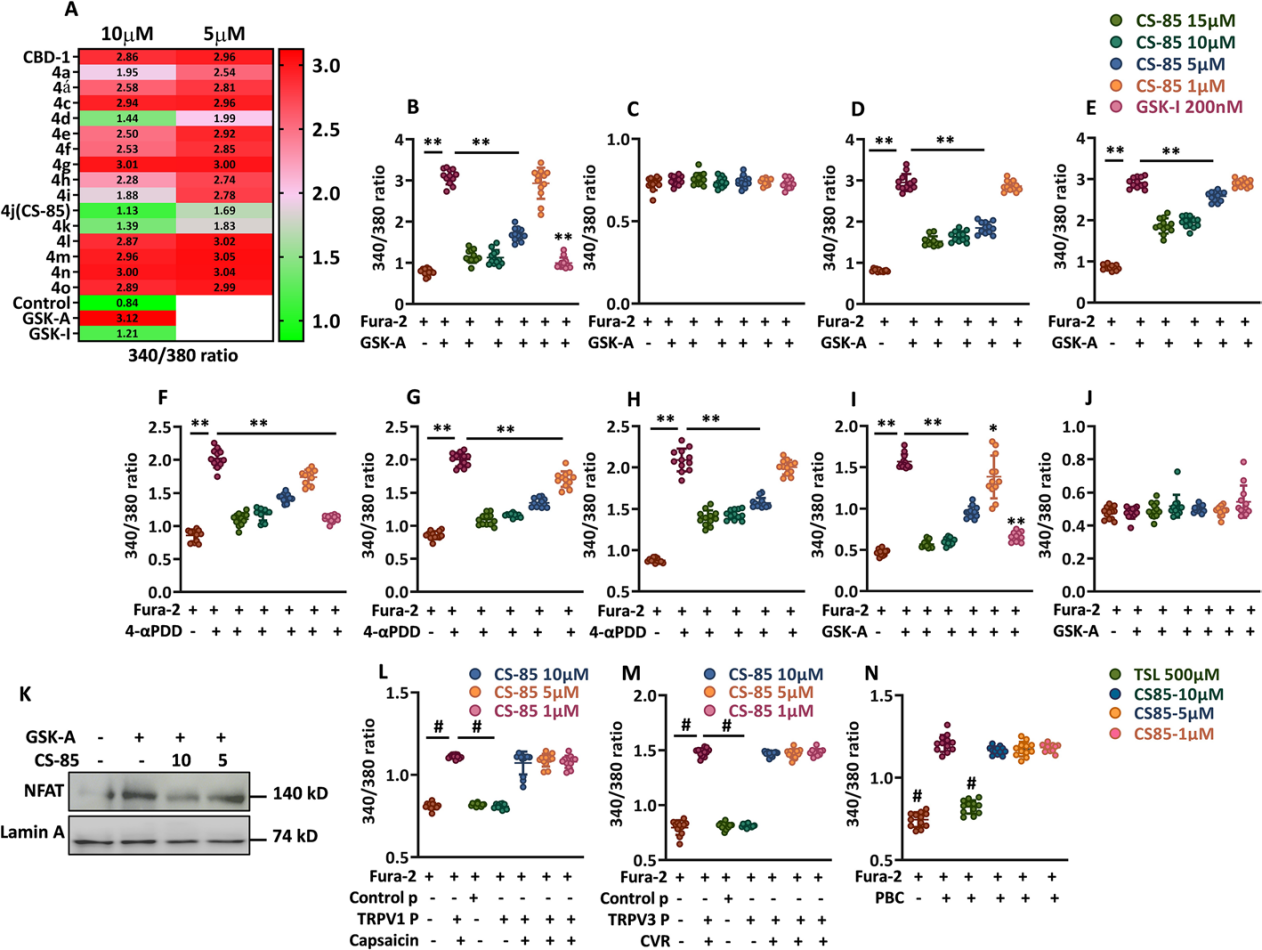
CS-85 blocks TRPV4 mediated Ca^2+^ influx into the macrophages. **A** Fura-2 AM dye (3 µM) was loaded on A375 cells for 40 min in the dark, followed by one wash with calcium-free HBSS. The cells were then treated with test compounds at indicated concentrations for 15 min, and subsequently stimulated with TRPV4specific agonist GSK-A (100 nM) for an additional 15 min in calcium-containing HBSS (1.8 mM). The fluorescence was recorded at wavelengths of 340/510 and 380/510 (*n* = 3). **B** Fura-2 AM-loaded A375 cells were washed with HBSS and then treated with indicated concentrations of CS-85 in calcium-containing HBSS for 15 min. The cells were then stimulated with GSK-A (100 nM) for an additional 15 min, and fluorescence was measured at 340/510 and 380/510. Data are presented as 12 individual points from three independently performed experiments (*n* = 3). **C** Fura-2 AM-loaded A375 cells were washed with HBSS, followed by treatment with an indicated concentration of CS-85 in calcium-free HBSS for 15 min. This was followed by stimulation of cells with GSK-A (100 nM) for an additional 15 min, in the same medium. The fluorescence was measured at 340/510 and 380/510. Data are presented as 12 individual points from three independently performed experiments (*n* = 3). **D**, **E** Fura-2 AM-loaded A375 cells were either concurrently stimulated with GSK-A (100 nM) and CS-85 at indicated concentrations for 15 min (**D**), or the cells were first stimulated with GSK-A (100 nM) for 3 min followed by adding CS-85 at indicated concentrations for another 12 min (**E**). The fluorescence was measured at 340/510 and 380/510. Data are presented as 12 individual points from three independently performed experiments (*n* = 3). **F**, **G**, **H** Fura-2 AM was loaded on A375 cells. The treatment conditions were: pretreatment (15 min CS-85 pretreatment followed by stimulation with 4 α-PDD, **F**), cotreatment (CS-85 added concurrently with 4 α-PDD, **G**), and therapeutic conditions (CS-85 added 5 min after 4 α-PDD treatment, **H**). The 4 α-PDD treatment in all the experimental conditions lasted 15 min. The fluorescence was measured at 340/510 and 380/510. Data are presented as 12 individual points from three independently performed experiments (*n* = 3). **I** BMDMs were incubated with Fura-2 AM for 40 min, followed by a single wash in calcium-free HBSS. Subsequently, the cells were treated with CS-85 for 15 min, followed by stimulation with GSK-A (100 nM) in calcium-containing HBSS (**I**) and calcium-free HBSS (**J**) for an additional 15 min. Data are presented as 12 individual points from three independently performed experiments (*n* = 3). **K** BMDMs were stimulated with indicated concentrations of CS-85 for 30 min, prior to GSK-A (100 nM) stimulation for 4 h. The cytosolic fraction of cells was prepared using cytoplasmic fractionation buffer, and the nuclear fraction of NFAT1 was extracted by using RIPA lysis, which was analyzed by immunoblotting. **L**, **M** RAW 264.7 cells (2 × 10^4^) were seeded in black optical-bottom plates. The cells were transfected with hTRPV1 or hTRPV3 plasmid DNA. 24 h after transfection, cells were loaded with Fura-2 AM dye for 40 min. The cells were then washed and treated with CS-85 at indicated concentrations for 15 min. The *Trpv1*-transfected cells were incubated with capsaicin, 5 µM for 12 min at 37 °C for TRPV1 activation (**L**), while *Trpv3*-transfected cells were stimulated with carvacrol (CVR), 300 µM for 12 min at RT for TRPV3 activation (**M**). **N** For TRPV2 activation, HCT (2 × 10^4^) cells were seeded in black optical-bottom plates. The cells were loaded with Fura-2 AM dye for 40 min and washed. The cells were then treated with CS-85 or tranilast (TSL) for 15 min and then stimulated with probenecid (1 mM) for another 9 min at RT. The fluorescence was measured at 340/510 and 380/510. Data are presented as 12 individual points from three independently performed experiments (*n* = 3). Data are presented as mean ± SD or individual data points. **p < 0.001, compared with either GSK-A- or 4 α-PDD-treated cells as indicated in the respective figures. ^#^*p* < 0.001 compared with capsaicin-, CVR-, or PBC-treated cells as indicated in panels **L**, **M**, and **N**

The activation of TRPV4 ion channel facilitates cellular Ca^2+^ influx, which acts as a second messenger, influencing cellular processes such as inflammation [44, 45]. We aimed to assess the TRPV4 antagonistic action of a focused series of CBD-**1** derivatives. The Ca^2+^ influx was quantified using a fluorescence-based assay by determining 340/380 nm ratio on a plate reader (Tecan Infinite 200 PRO). To achieve this, Fura-2 AM loaded A375 cells were treated with test compounds, followed by stimulation with specific TRPV4 agonist GSK1016790A (GSK-A) to induce Ca^2+^ influx (Fig. 2A and Supplementary Fig. S70A). The results showed that the test compound treatment reduced Ca^2+^ entry to varying extents with some significantly reducing influx (Fig. 2A) without affecting cell viability at the tested concentrations (Supplementary Table S5). Of all the compounds tested, CS-85 demonstrated the most significant results. We then tested CS-85 at multiple concentrations to determine the optimal inhibition of TRPV4 channel, which was achieved between 5 µM and 15 µM (Fig. 2B). We also observed that CS-85 pretreatment specifically blocks TRPV4 mediated Ca^2+^ influx without affecting intracellular calcium signaling, under calcium-free conditions (Fig. 2C). After confirming the inhibitory action on TRPV4 channel under pretreatment conditions, we evaluated the effect of CS-85 under cotreatment (added concurrently with GSK-A; Fig. 2D) and therapeutic (added after GSK-A treatment; Fig. 2E) conditions. Interestingly, CS-85 demonstrated a marked inhibition of Ca^2+^ influx in these two conditions as well.

To validate these findings, we used another well-known TRPV4 agonist, 4 alpha phorbol 12,13-didecanoate (4α-PDD) (Supplementary Fig. S70B). The effect of CS-85 was evaluated under three experimental conditions, as above: pretreatment (Fig. 2F), cotreatment (Fig. 2G), and therapeutic conditions (Fig. 2H). All the three conditions validated CS-85 as a TRPV4 inhibitor and corroborated the findings obtained in the preceding experiments (Fig. 2B,D,E). We reaffirmed our findings by performing an imaging experiment with another calcium-sensitive dye, Fluo-3 AM, in A375 cells, which confirmed that CS-85 strongly inhibits TRPV4 mediated calcium influx in A375 (Supplementary Fig. S72 A, B).

After identifying CS-85 as a TRPV4 inhibitor in the A375 cell line, we aimed to evaluate its inhibitory potential in primary macrophages, specifically bone marrow derived macrophages (BMDMs). The findings from BMDMs (Figs. 2I,J) corroborate the results observed in the A375 cell line (Fig. 2A–C). To further evaluate the specificity of CS-85, we stimulated TRPV4 by its specific ligand GSK-A and measured NFAT1 activation, as readout of TRPV4 activity (Fig. 2K and Supplementary Fig. S73) as demonstrated previously [44, 33]. NFAT is a downstream proinflammatory target of TRPV4 in macrophages that amplifies inflammatory responses in the lung, resulting in severe lung dysfunction [33, 46, 44]. The experiment demonstrated that TRPV4 activation induced by GSK-A led to maximal NFAT activation, but this effect was significantly reduced by treating the cells with CS-85 (Fig. 2K and Supplementary Fig. S73). Therefore, the results demonstrate that CS-85 significantly antagonizes both human (Fig. 2A–H) and mouse TRPV4 ion channels (Fig. 2I–K) in response to GSK-A- or 4α-PDD-induced activation, without affecting cell viability (Supplementary Fig. S71A,B).

We next assessed the interaction of CS-85 with other related TRPV channels such as TRPV1, TRPV2, and TRPV3 (Fig. 2L–N and Supplementary Fig. S74A–C). For TRPV1 and TRPV3 activation, RAW 264.7 cells were transfected with either hTRPV1 (Supplementary Fig. S75A) or hTRPV3 (Supplementary Fig. S75B) plasmid DNA, respectively. The cells were treated with CS-85, followed by the respective indicated agonists to induce Ca^2+^ influx. For TRPV2 activation, we used HCT-116 cell line (human colon cell line) owing to its comparatively higher expression of *Trpv2*. The cells were treated with CS-85, followed by its TRPV2 agonist. CS-85 did not affect Ca^2+^ entry mediated by TRPV2 (Fig. 2N) or TRPV3 (Fig. 2M), but produced mild nonsignificant reduction in TRPV1-mediated (Fig. 2L) Ca^2+^ influx into the cells. These findings indicate good specificity of CS-85 toward TRPV4.

### CS-85 inhibits proinflammatory cytokine release

**Fig. 3:**
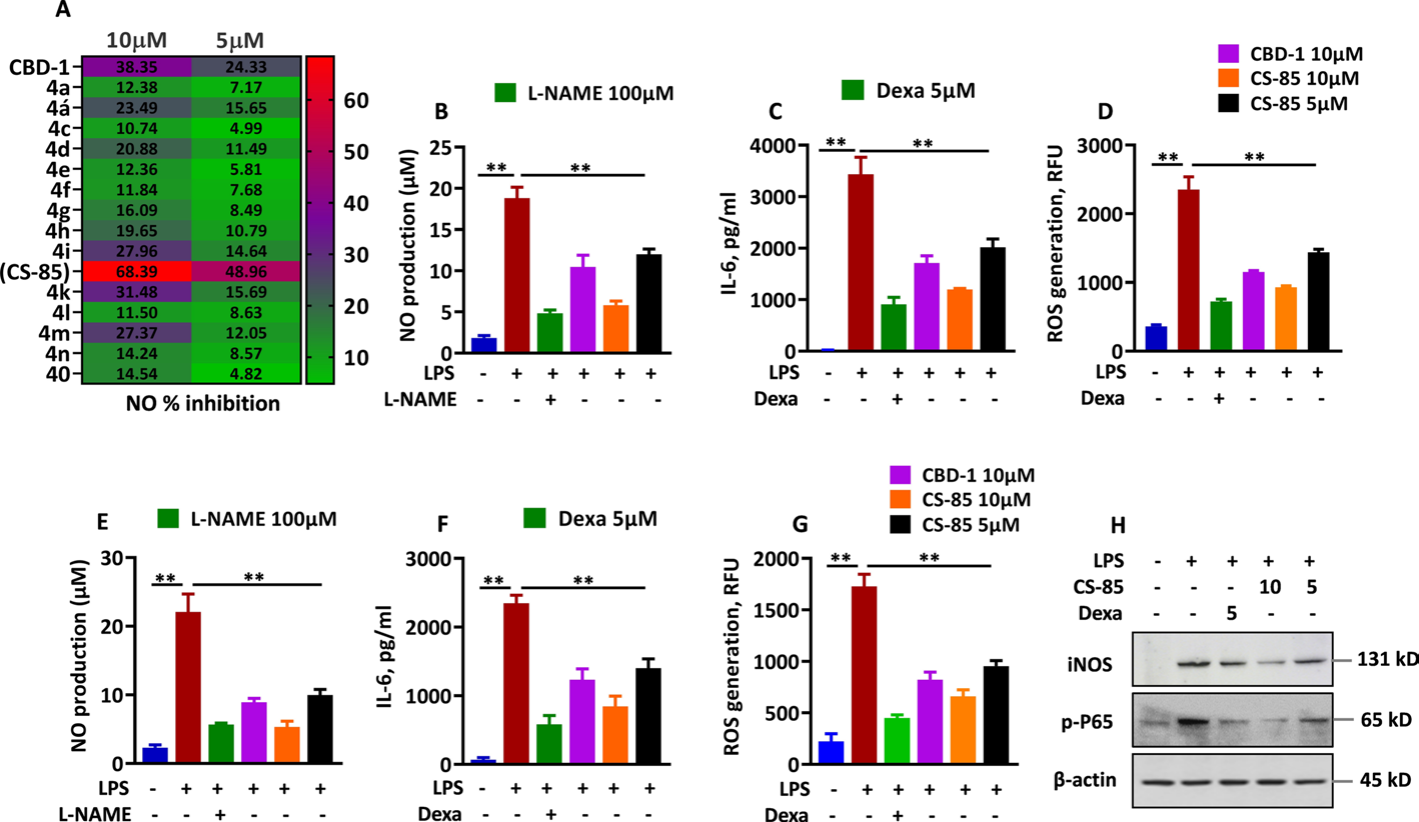
CS-85 inhibits production of proinflammatory cytokines. **A** RAW 264.7 cells were pretreated with test compounds, including CS-85 and parent molecule CDB-1, for 1 h, before being exposed to LPS (1 µg/mL) for 24 h. After 24 h, the supernatants were collected and analyzed for percentage of nitric oxide inhibition. **B**, **C** RAW 264.7 cells were pretreated with indicated concentration of CS-85, CBD-**1**, L-NAME (100 μM), or dexamethasone (5 μM), followed by LPS (1 μg/mL) exposure for 24 h. After 24 h, the supernatants were collected and analyzed for NO inhibition (**B**) and IL-6 release (**C**). **D** RAW264.7 cells were treated with indicated concentrations of CS-85, CBD-**1**, or dexamethasone (5 μM) for 1 h, and subsequently exposed to LPS for 24 h. After 24 h, the cells were incubated with DCFH-DA (5 µM) for 30 min and then washed with PBS. ROS production was measured by using a spectrofluorometer plate reader at a wavelength of 493 nm and 527 nm, respectively. **E**, **F** BMDMs were pretreated with a specified concentration of CS-85, CBD-**1**, L-NAME (100 μM), or dexamethasone (5 μM) for 1 h followed by LPS (1 μg/mL) stimulation for 24 h. After 24 h, the supernatants were collected and analyzed for NO inhibition (**E**) and IL-6 release (**F**). **G** BMDMs were pretreated with indicated concentrations of CS-85, CBD-**1**, or dexamethasone (5 μM) for 1 h, and subsequently challenged with LPS for 24 h. After 24 h, the cells were incubated with DCFH-DA (5 µM) for 30 min and washed with PBS. Release of ROS was recorded using a spectrofluorometer plate reader, at 493 nm excitation wavelength and 527 nm emission wavelength, respectively. **H** BMDMs were treated with CS-85 or dexamethasone (5 μM) for 1 h, followed by LPS stimulation for 24 h. After 24 h, the cell lysates were prepared and analyzed for phospho-p65 and iNOS by immunoblotting. Representative blots are shown from at least three independently performed experiments (*n* = 3). Data are presented as mean ± SD. **p < 0.001, compared with LPS-treated cells

The TLR4/MyD88 pathway is crucial for regulating LPS induced inflammation [47], and targeting this pathway offers a means to control excessive inflammation. We assessed the anti-inflammatory potential of test compounds on RAW 264.7 macrophages stimulated with LPS (1 μg/mL), a well-known inducer of inflammation. We measured IL-6, nitric oxide (NO) inhibition, and ROS generation by absorbance or fluorogenic probe DCFH-DA-based assays. The cells were pretreated with test compounds at 5 and 10 µM before LPS stimulation, and supernatants were analyzed for NO inhibition. Several compounds from the series exhibited strong inhibition of NO release (Fig. 3A) without impacting cell viability (Supplementary Table S6). CS-85 demonstrated the highest inhibition with ~49% at 5 µM and 68.3% at 10 µM (Fig. 3A). CS-85 more effectively inhibited proinflammatory markers such as IL-6, NO, and ROS across multiple concentrations compared with its parent compound, CBD-**1**, in these cells (Fig. 3B–D). Additionally, when reassessed in BMDMs, CS-85 significantly reduced LPS induced oxidative stress and inflammation, as indicated by decreased levels of NO, IL-6, and ROS (Fig. 3E–G). Given that NF-ĸB is a prime transcription factor activated by LPS, and its phosphorylated form regulates most of the proinflammatory genes, including iNOS [48], which primarily drives NO production in macrophages [49], we measured phosphorylation of NF-ĸB and iNOS expression in BMDMs by immunoblotting (Fig. 3H and Supplementary Fig. S76A,B). We found that prior treatment with CS-85 rescued the phosphorylation of NF-ĸB and expression of iNOS compared with the LPS-treated cells (Fig. 3H and Supplementary Fig. S76 A,B).

### CS-85 treatment reduces acute lung injury in mice

Before conducting the *in vivo* studies, we assessed CS-85 for potential adverse effects following endotracheal administration, across a dose range of 30, 300, and 1500 µg per mouse, to identify a safe therapeutic dose (Supplementary Fig. S77A–C). For all *in vivo* experiments, CS-85 was dissolved in 5% DMSO, 5% Tween 80, 35% PEG-40, and 55% quantum satis (qs) water vol/vol. A 50µL volume of this formulation was administered per mouse via noninvasive endotracheal route, as demonstrated earlier [50]. Multiple biochemical parameters were evaluated, such as IL-1β (Supplementary Fig. S77A), IL-6 (Supplementary Fig. S77B), and myeloperoxidase (MPO) (Supplementary Fig. S77C), as indicators of epithelial–alveolar damage, inflammation, and neutrophilia. None of the parameters exhibited a significant increase compared with the control mice (Fig. S77A–C).

**Fig. 4:**
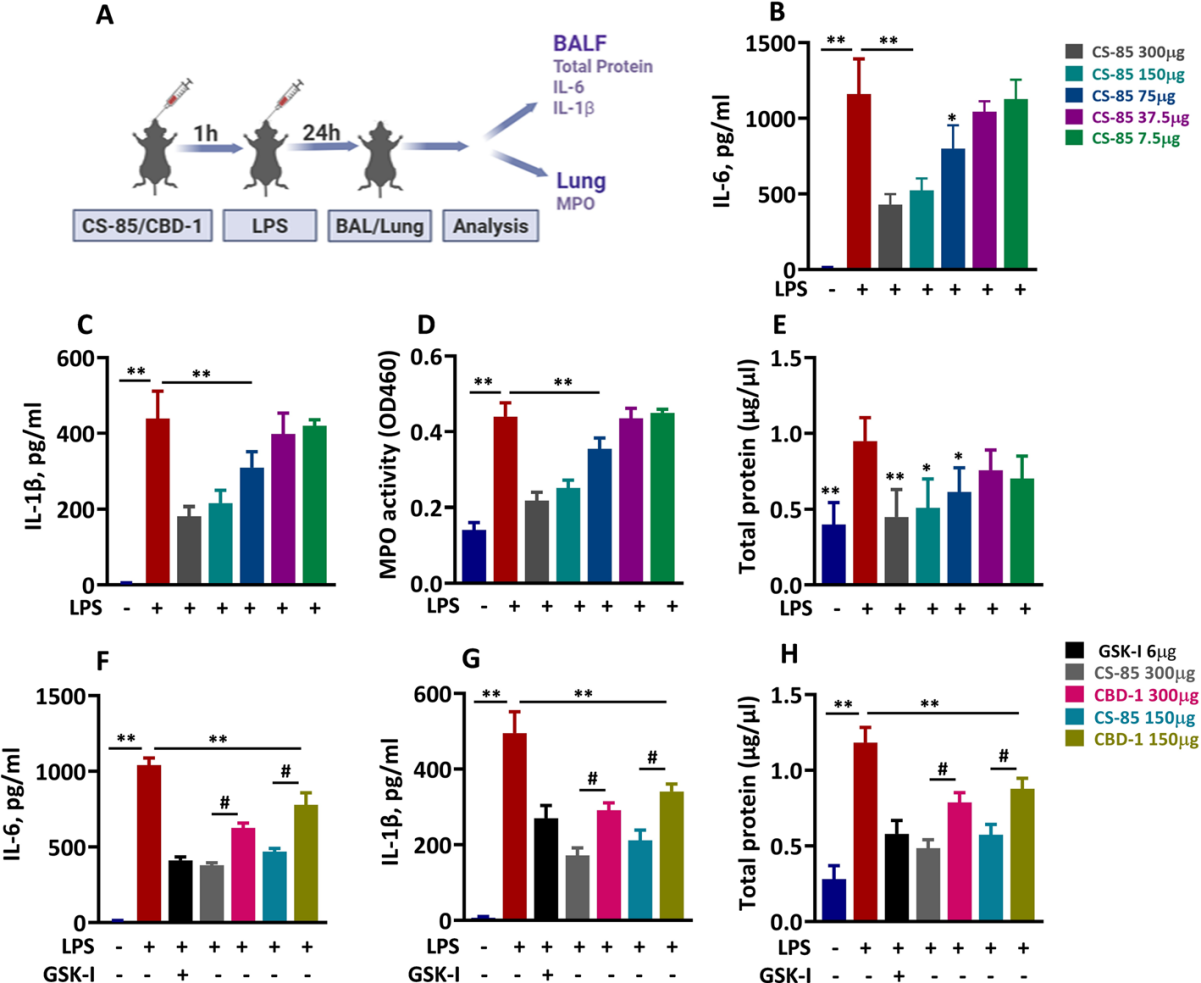
CS-85 reduces lung inflammation injury in mice. **A** Schematic presentation of lung inflammation following LPS induced injury. **B**, **C** The mice were anesthetized with a ketamine–xylazine cocktail (80 and 15 mg/kg, respectively). The anesthetized mice were pretreated with CS-85 (7.5, 37.5,75, 150, and 300 µg/mouse), which was administered endotracheally 60 min prior to exposure of LPS (75 µg/mouse). Then, 24 h after LPS exposure, BAL was performed on mice, and their lungs were collected and homogenized. Cytokines IL-6 (**B**) and IL-1β (**C**) were estimated in BAL fluid from the above mentioned mice by ELISA (*n* = 5). **D** The lung homogenate was used to perform the MPO assay, as indicated in scheme A (*n* = 5). **E** The total protein content in the BAL fluid was determined by using the Bradford method (*n* = 5). **F**, **G**, **H** The mice were pretreated with CBD-**1** or CS-85 followed by LPS exposure. BAL fluid was collected, and IL-6 (**F**), IL-1β (**G**), and total protein levels (**H**) were quantified (*n* = 5). The data are presented as mean ± SD. **p < 0.001, *p < 0.05 compared with LPStreated mice, ^#^*p* < 0.001 compared with corresponding CS-85-treated mouse group

We subsequently conducted an in depth study on the effects of CS-85 on ALI induced by LPS, a widely used agent to induce the inflammatory response in murine models of ALI [51]. As an initial step, we conducted a pilot study across a dose range of 7.5, 37.5, 75, 150, and 300 µg/mouse, and measured inflammatory parameters such as IL-6, IL-1β, MPO, and total protein to identify a therapeutic dose (Fig. 4A–E). The findings identified 150 and 300 µg/mouse as the most effective doses, which markedly reduced inflammatory markers in the lung and were therefore selected for further experiments (Fig. 4B–E). Additionally, we evaluated the comparative efficacy of CS-85 with its parent compound, cannabidiol (CBD-**1**) in an LPS induced ALI model. Both CS-85 and CBD-**1** reduced levels of proinflammatory parameters significantly (Fig. 4F–H). However, the reduction was more pronounced in CS-85-treated mice, which comparatively demonstrated significant reduction in IL-6 (Fig. 4F), IL-1β (Fig. 4G), and total protein (Fig. 4H) levels at indicated doses. The reduction in inflammatory parameters was significantly more in CS-85-treated mice as compared with CBD-**1**-treated mice at respective doses.

### CS-85 reduces lung vascular permeability and edema in mice, preventing lung damage and associated cellular infiltration

**Fig. 5:**
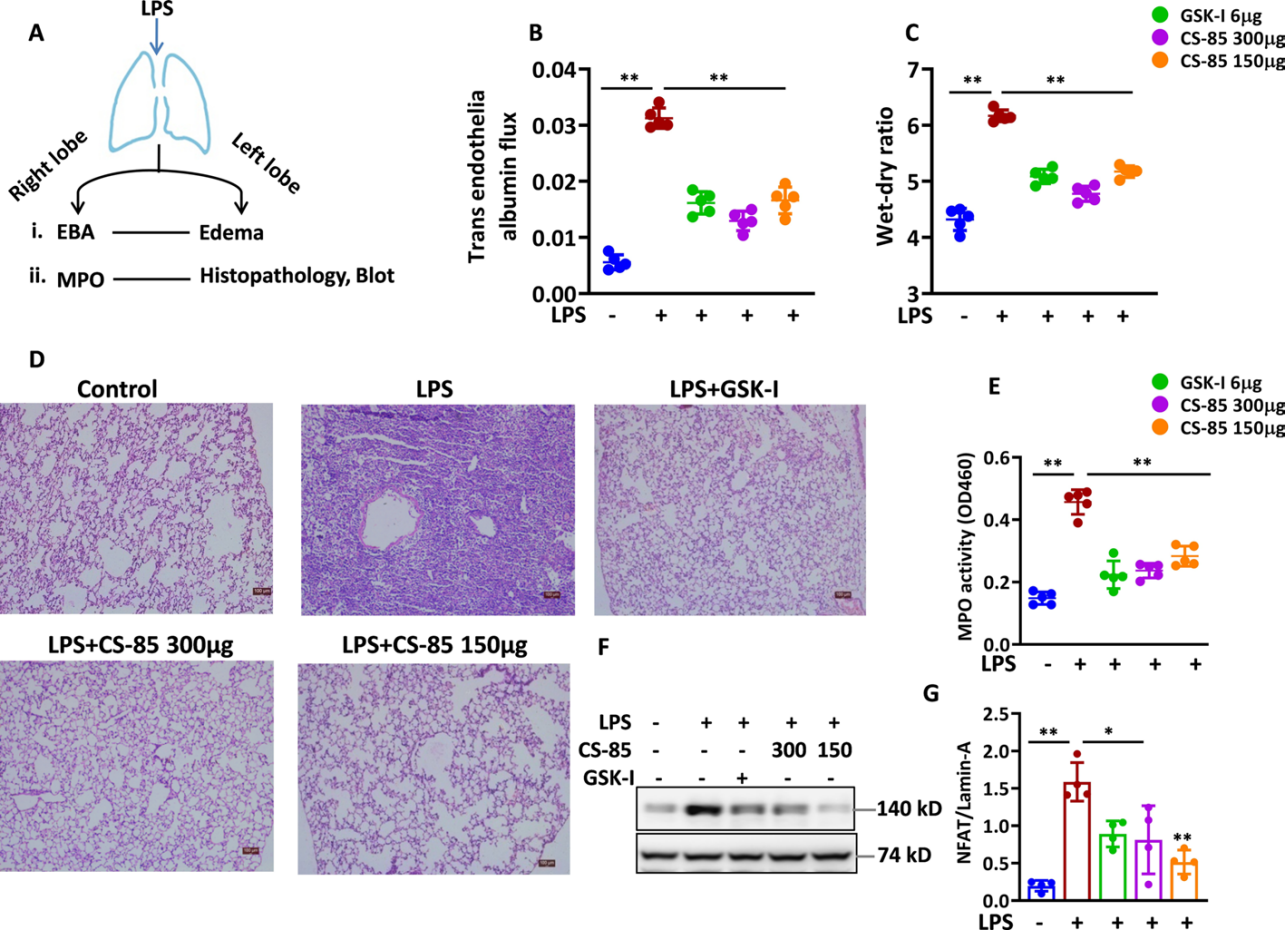
CS-85 maintains lung vascular integrity and reduces pulmonary edema. **A** Schematic presentation of LPS induced acute lung injury. **B**, **C** Mice were treated with CS-85 or GSK-I, endotracheally at indicated doses 60 min prior to exposure of LPS (75 µg/mouse) for 24 h. Then, 30 min before sacrificing the mice, 100 μL/mouse of albumin-tagged Evans blue was injected retro-orbitally. The lung vascular leakage was determined by measuring albumin influx (**B**), and lung edema was measured by lung wet-to-dry ratio (**C**) (*n* = 5). **D**, **E** In a separate cohort, mice were treated with CS-85 or GSK-I before being exposed to LPS. Then, 24 h after LPS exposure, the mice were sacrificed, and lungs were harvested. The left lung lobe from the mice was fixed in formalin, sectioned, and stained with hematoxylin and eosin. Representative images (scale bars, 100 µm) are shown (**D**). The right lung lobes were homogenized, and MPO activity was determined (**E**) (*n* = 5). **F** Alveolar macrophages were harvested by BAL 24 h after LPS treatment. The cytoplasmic and nuclear fractions were extracted by cytoplasmic fractionation and RIPA lysis buffer, respectively. Nuclear NFAT1 levels were analyzed by immunoblotting. A representative blot is shown (*n* = 4). **G** The graph shows densitometry analysis of NFAT1 of four independent experiments. The data are presented as mean ± SD or data points from individual mice. **p < 0.001, *p < 0.05 compared with LPS-treated mice

ALI is characterized by compromised lung vascular permeability and pulmonary edema formation [52], and LPS is known to effectively induce these features in animal models. To assess the efficacy of CS-85, we investigated its impact on lung vascular leakage and edema in an LPS induced ALI model. Lung vascular leakage was assessed by measuring the extravasation of Evans blue-labeled albumin, while lung edema was evaluated by determining the lung wet-to-dry weight ratio (Fig. 5A–C). LPS induced inflammatory vascular injury at 24 h was observed by an increased lung wet-to-dry weight ratio and enhanced transendothelial albumin extravasation in the LPS-treated mouse group (Fig. 5B,C). Interestingly, CS-85 at 150 and 300 µg/mouse effectively maintained the endothelial integrity and prevented LPSinduced vascular leakage (Fig. 5B). CS-85 markedly reduced edema formation and fluid accumulation, as indicated by a reduction in the wet-to-dry ratio of the lung (Fig. 5C).

Cellular infiltration into the lungs during ALI intensifies the inflammatory environment by releasing proinflammatory mediators and enzymes that damage the lung architecture [53]. We pretreated mice with CS-85 and exposed them to LPS. We measured MPO in the lungs as an indicator of inflammatory status and specifically to assess neutrophil infiltration. Additionally, we assessed the changes in lung architecture and inflammatory cell recruitment resulting from ALI. The LPS-treated group of mice exhibited a significant increase in MPO activity (Fig. 5E) and intra-alveolar infiltrates in the lung sections as visualized on H&E-stained slides (Fig. 5D), indicating high cellular infiltration, while the CS-85-treated mice showed comparatively low MPO level and intact lung architecture (Fig. 5D,E), Overall, our findings suggest that CS-85 may be effective in preserving lung architecture and preventing the influx of inflammatory infiltrates to the lung during ALI. Next, we assessed NFAT activation in the lungs of CS-85-pretreated, LPS exposed mice to determine whether our *in vitro *findings were recapitulated *in vivo*. We found that LPS lead to maximum NFAT activation (Fig. 5F,G), which was however markedly reduced by CS-85 or GSK-I treatment (Fig. 5F,G), indicating that CS-85 exerts its anti-inflammatory effects via NFAT, a known downstream signaling event in TRPV4 pathway.

### CS-85 reduces severe inflammation, triggered by *Trpv4* overexpression via liposome-mediated *in vivo* gene delivery and its pharmacological overactivation

**Fig. 6:**
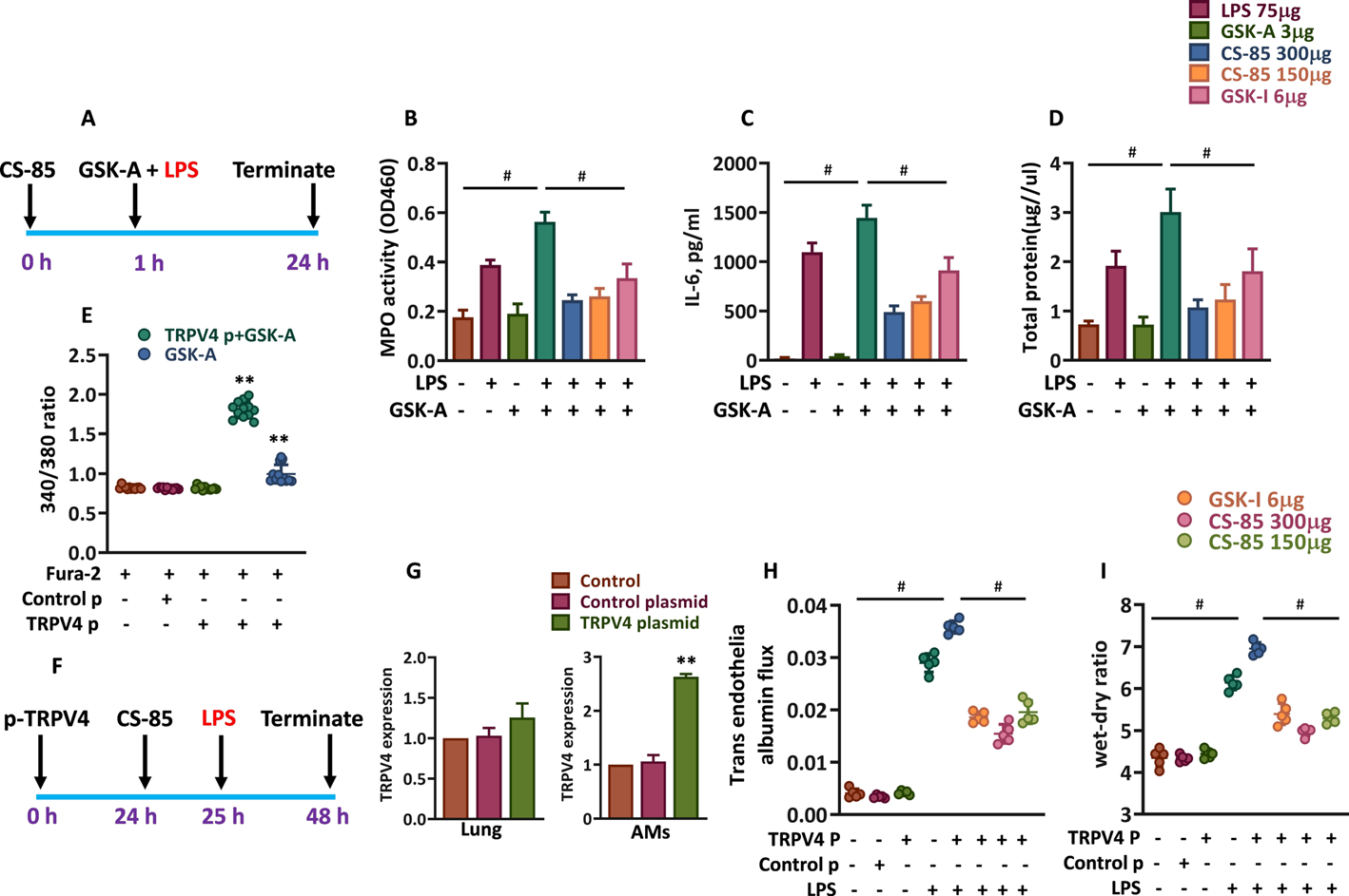
Magnitude of TRPV4 activation and expression influences lung injury. **A** Schematic outline illustrating CS-85 treatment schedule, lung injury induction, and GSK-A + LPS-mediated hyperactivation of TRPV4. **B** Mice were treated with CS-85 at indicated doses, 60 min prior to coexposure of LPS and GSK-A (3 μg, 50 µL per mouse), administered via endotracheal route. Then, 24 h after LPS + GSK-A exposure, BAL was performed on mice, and lungs were collected and homogenized. The lung homogenate was used for MPO activity estimation (*n* = 5). **C**, **D** The BAL fluid was collected and analyzed for cytokine IL-6 (**C**) and total protein content (**D**) (*n* = 5). **E** RAW 264.7 cells (2 × 10^4^/well) seeded in a black 96-well plate were transfected with hTRPV4 plasmid. Then, 24 h after transfection, the cells were loaded with Fura-2 AM at RT in incomplete DMEM. The cells were washed and stimulated with GSK-A (100 nM) for 15 min. The fluorescence was measured at 340/510 and 380/510 (*n* = 3). **F** Schematic outline illustrating TRPV4 loaded liposome delivery into mice, CS-85 treatment schedule, lung injury induction, and the experimental termination protocol. **G** *Trpv4* expression was measured by real-time qPCR in alveolar macrophages (AMs) (**G**) and lung homogenate (**G**) to confirm the successful delivery and expression of *Trpv4* complementary DNA (cDNA). GAPDH was used as a control (*n* = 3). **H**, **I** *Trpv4 *overexpressed mice were treated with CS-85 or GSK-I (6 µg/mouse), and subsequently exposed to LPS (75 µg/mouse), as indicated in the outline. The right lung lobes were collected and used to estimate albumin leakage (**H**), and the left lobe of each mouse was used for edema estimation by measuring wet-to-dry ratio (**I**) (*n* = 5). Data are expressed as mean ± SD and/or individual data points, each corresponding to a single mouse. ^#^*p* < 0.001 compared with either LPS + GSK-A (panels B–D) or LPS + p-TRPV4 (panels H–I) mice. **p < 0.001 compared with control cells (panel E) or control mice (panel G)

LPS is known to induce TRPV4 activation and amplify TLR4-NF-κB-mediated inflammatory signaling [44, 33]. This activation produces blebs and breaks in epithelial and endothelial layers of the alveolar septal wall [54], exacerbating lung injury. Above all, prolonged or excessive activation of TRPV4 is particularly associated with severe lung dysfunction [54, 55, 56]. In the present study, we adopted two approaches to test efficacy of CS-85 against TRPV4 mediated severe lung inflammation. Firstly, in a mouse model of ALI, we overactivated TRPV4 by cotreatment of mice with GSK-A and LPS (Fig. 6A) and assessed inflammatory parameters directly associated with neutrophil recruitment and lung inflammatory edema such as MPO, IL-6, and total protein (Fig. 6B–D). We observed a marked increase in these parameters in mice coexposed to GSK-A and LPS, compared with those exposed to LPS or GSK-A alone (Fig. 6B–D). Notably, such severity in inflammation was not seen in mice treated solely with LPS. Pretreatment with CS-85 significantly attenuated this TRPV4 mediated hyperinflammation and associated parameters induced by GSK-A and LPS coexposure, reducing them nearly to basal levels (Fig. 6B–D). On the basis of these findings, we reiterate that magnitude of TRPV4 activation determines the intensity of pathophysiology in ALI. To establish proof of concept, we overexpressed *Trpv4* in RAW264.7 cells and measured Ca^2+^ influx, induced by GSK-A (Fig. 6E). A markedly increased Ca^2+^ influx was observed in *Trpv4* overexpressing cells as compared with nontransfected cells, indicating that *Trpv4* expression can directly determine its downstream calcium signaling. Therefore, we used a related approach in a mouse ALI model, where we overexpressed *Trpv4* in AMs *in vivo* through liposome-mediated delivery of Trpv4 plasmid DNA as shown in Fig. 6F. The expression of *Trpv4* in transfected AMs increased ~2.5-fold compared with the control AMs (Fig. 6G). Following liposome administration, the mice were pretreated with CS-85 before LPS exposure, as indicated in the outline (Fig. 6F). It was observed that lung vascular permeability and edema were significantly exacerbated in LPS exposed mice with *Trpv4* overexpression, compared with the group exposed to LPS alone (Fig. 6H,I). This finding suggests that *Trpv4* transfection in AMs increased TRPV4 activity following LPS exposure, resulting in a more severe inflammatory response as compared with the LPS only group (Fig. 6H,I). We observed that CS-85 pretreatment markedly reduced the hallmark features of ALI such as lung vascular leakage and edema, indicating that CS-85 has a promising anti-inflammatory potential in lung. This suggests that CS-85 effectively interacts with and blocks TRPV4 mediated signaling, mitigating the heightened inflammatory response.

**Fig. 7:**
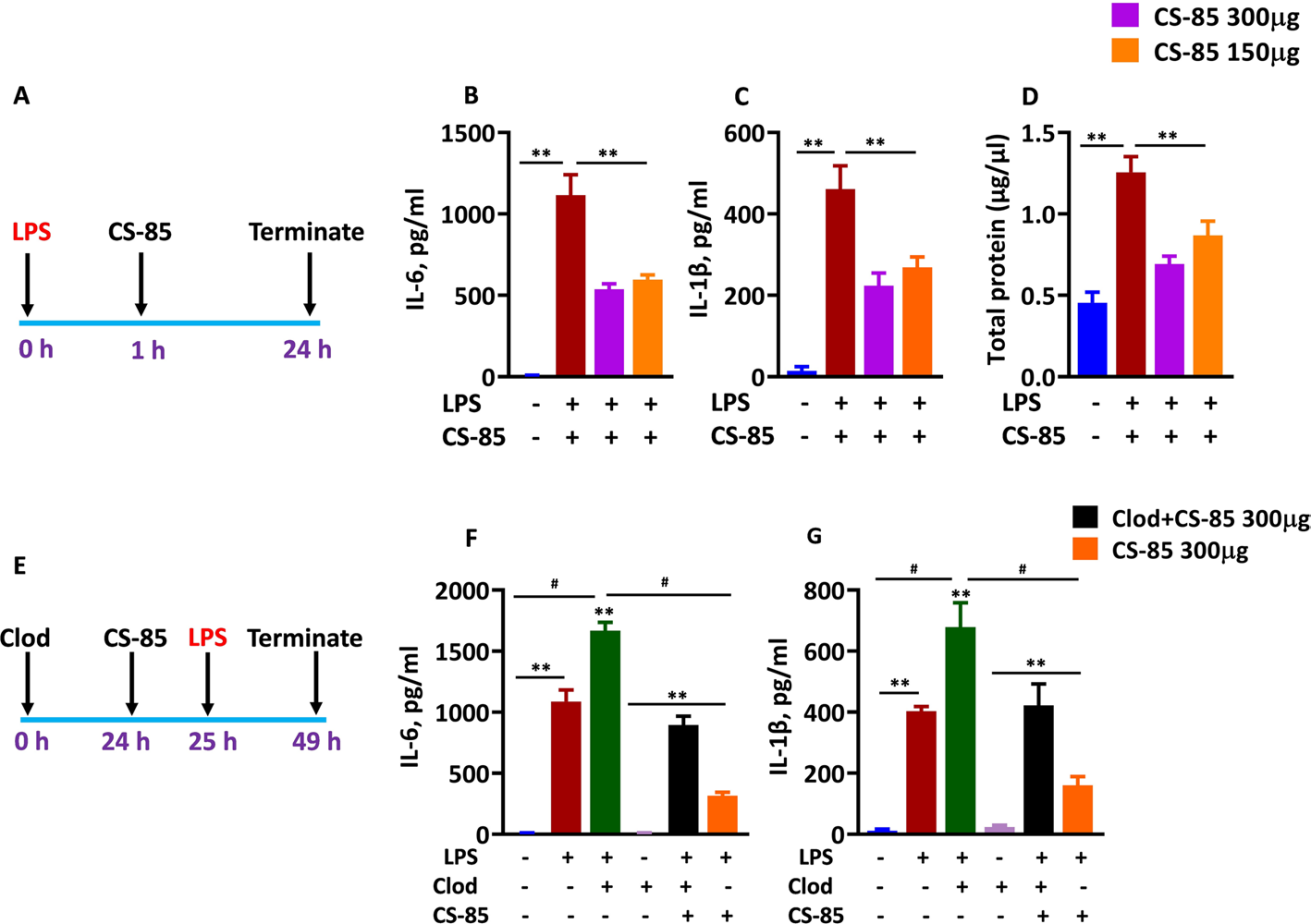
CS-85 attenuates ALI features in a therapeutic model of ALI. **A** Schematic presentation of LPS induced therapeutic model of ALI. **B**, **C** The mice were anesthetized with a ketamine–xylazine cocktail (80 and 15 mg/kg, respectively). The anesthetized mice were exposed to LPS (75 µg/mouse) for 1 h followed by CS-85 treatment for 24 h. BAL was performed 24 h after CS-85 treatment, and cytokines IL-6 (**B**) and IL-1β (**C**) were estimated by ELISA (*n* = 5). **D** The total protein content in the BAL fluid of the above mentioned mice was determined by using the Bradford method (*n* = 5). **E** Schematic presentation of experimental timeline showing clodronate, LPS administration and CS-85 treatment in mouse model of ALI. The clodronate liposomes (70 μL/mouse) were administered endotracheally to anesthetized mice. CS-85 treatment was given 1 h prior to LPS (75 µg/mouse) instillation. The BAL was performed 24 h after LPS instillation. **F**, **G** The BAL fluid of the mice was analyzed for IL-6 (**F**) and IL-1β (**G**) levels by ELISA (*n* = 5). The data are presented as mean ± SD. **p < 0.001 compared with LPS-treated mice, ^#^*p* < 0.001 compared with Clod + LPS-treated mice (panels F and G)

Next, we tested the efficacy of CS-85 in a therapeutic mouse model of LPS induced ALI (Fig. 7A). We quantified inflammatory cytokines such as IL-6 and IL-1β and assessed total protein accumulation in the lung alveoli as an indication of alveolar barrier leak and resulting edema. Interestingly, we observed a marked decrease in IL-6 (Fig. 7B) and IL-1β (Fig. 7C) release and also total protein levels (Fig. 7D) at indicated doses. The magnitude of reduction in these parameters corroborated with prophylactic model (Fig. 4B,C,E), adding a new dimension in the efficacy portfolio of CS-85. Next, we wanted to investigate the cellular target of CS-85 in our disease model. Toward this end, we used clodronate liposomes (Clod) to deplete AMs from lung and determined lung injury following LPS challenge (Fig. 7E). Consistent with the above findings (Figs. 7B,C and 4B,C), CS-85 markedly reduced inflammatory parameters (with AMs present in the lung) as compared with LPS treated mice alone (Fig. 7F,G). In contrast, depletion of AMs weakened the anti-inflammatory property of CS-85 (Clod + LPS + CS-85). Notably, the absence of CS-85 further escalated cytokine release in Clod + LPS mice (Fig. 7F,G), indicating that CS-85 possibly exerts its anti-inflammatory effects via AMs. However, cytokine levels remained low in Clod + LPS + CS-85 mice as compared with Clod + LPS, indicating that CS-85 may modestly modulate other cell types as well. Collectively, these data indicate that CS-85 exerts its anti-inflammatory effects predominantly through interactions with AMs and partial contribution from other inflammatory cell populations.

### CS-85 downstream signaling

**Fig. 8:**
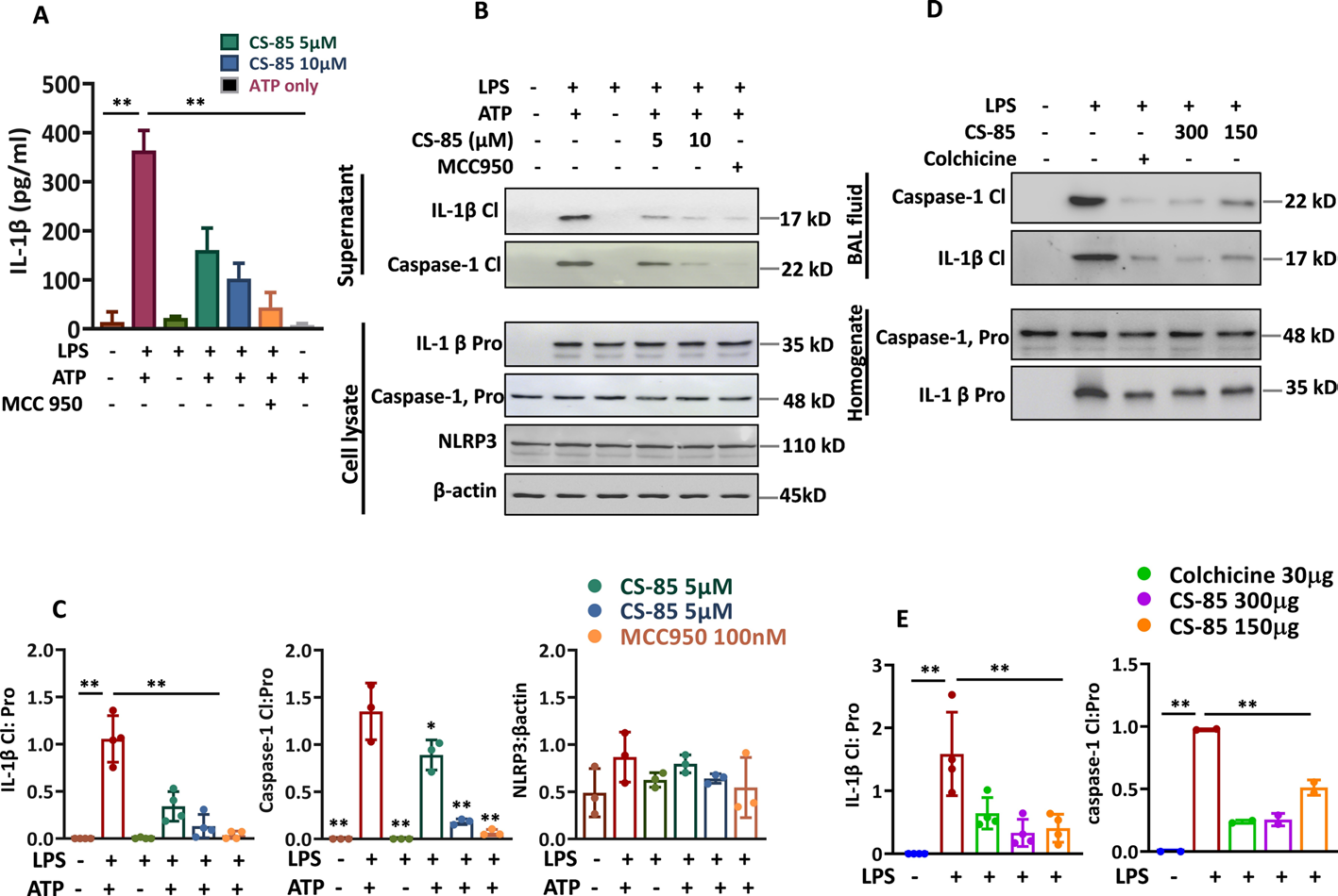
CS-85 blocks NLRP3 inflammasome activation. **A**. LPS-primed BMDMs were treated with indicated concentration of CS-85, or MCC950 (100 nM) for 1 h, followed by activation with ATP (5 mM, 30 min). The supernatants were collected and used for IL-1β estimation by ELISA. **B** LPS-primed BMDMs were treated with indicated concentration of CS-85, or MCC950 (100 nM) for 1 h, followed by activation with ATP (5 mM, 30 min). The supernatants were analyzed for cleaved IL-1β and cleaved caspase-1 by immunoblotting, whereas the cell lysates were used for estimation of pro-IL-1β, pro-caspase-1, and NLRP3. β-actin was used as loading control. Data are presented as mean + SD of three independent experiments. **C** The graph shows the densitometry analysis of indicated proteins in Fig. 7B for at least three independently performed experiments (*n* = 3). **D** Mice were subjected to CS-85 or colchicine treatment for 1 h, followed by LPS instillation (75 µg/mouse). Colchicine was administered via intraperitoneal route at 1 mg/kg of body weight. Then, 24 h after LPS exposure, BAL was performed on mice, and their lungs were collected and homogenized. The lung homogenates were analyzed for pro-IL-1β and pro-caspase-1, whereas cleaved-IL-1β and cleaved-caspase-1 were assessed from BAL fluid (*n* = 3). **E** The graph shows the densitometry analysis of indicated proteins in Fig. 7D for three experiments (*n* = 3). The data are presented as mean ± SD. **p < 0.001, *p < 0.05 compared with either LPS + ATP-treated cells or LPS-treated mice

The Ca^2+^–TRPV4 axis has been shown to activate several inflammatory pathways crucial to ALI pathogenesis, including NF-κB [57], NFAT [57], and the NLRP3 inflammasome pathway [58, 59]. We investigated whether CS-85 exerts its anti-inflammatory effect as a TRPV4 inhibitor by modulating these pathways across various experimental contexts. We selectively stimulated these pathways and then assessed their activation by western blot following CS-85 treatment. NFAT signaling was activated by GSK-A (TRPV4-NFAT) (Figs. 2K and 5F) and TLR4 by LPS (Fig. 3H). In addition, NLRP3 inflammasome assembly was induced by sequential LPS priming followed by ATP stimulation (Fig. 8A).

We observed that CS-85 significantly reduced NFAT activation (Figs. 2K and 5F) and NFκB phosphorylation (Fig. 3H). The TRPV4–NLRP3 axis plays a crucial role in various pathologies, with TRPV4 inhibition or knockdown reducing NLRP3 inflammasome activation and associated events [58, 59]. Therefore, we assessed the impact of CS-85 on the activation of the NLRP3 inflammasome complex in BMDMs. We performed an NLRP3 inflammasome activation assay by using extracellular ATP in LPS primed mouse BMDMs and found that CS-85 significantly reduced IL-1β release into the supernatant by ELISA (Fig. 8A). This initial finding prompted us to further investigate this pathway. We further evaluated the effect of CS-85 treatment on caspase-1 activation and IL-1β maturation/cleavage, two key sequential steps in NLRP3 inflammasome activation. Our findings showed that CS-85 inhibited NLRP3 inflammasome assembly by blocking caspase-1 activation (p22 subunit) and IL-1β cleavage (p17 subunit) (Fig. 8B,C). The densitometry analysis of IL-1β, caspase-1, and NLRP3 in Fig. 8B is shown (Fig. 8C).

Given that the NLRP3 inflammasome is known to be activated in ALI and plays a crucial role in its development and pathogenesis [28], we aimed to assess whether our *in vitro* findings could be replicated in our ALI model used in this study and whether CS-85 influences this pathway. We analyzed the BAL fluid and lung homogenate from these mice for effect on IL-1β and caspase-1 maturation/activation and found that CS-85 significantly inhibited IL-1β cleavage (p17 subunit) and caspase-1 activation (p22 subunit) (Fig. 8D,E). The densitometry analysis of IL-1β and caspase-1 in Fig. 8D is shown (Fig. 8E). Overall, this data suggest that CS-85 effectively inhibits the NLRP3 inflammasome signaling cascade in ALI, likely through Ca^2+^ TRPV4 mediated signaling.

## Discussion

ARDS treatment is largely supportive. However, there are several therapeutic approaches under clinical trials based on small-molecule inhibitors [60, 61] or biological interventions [61, 62]. Additionally, cell-based therapy has shown promising results in clinical and preclinical studies, which involved the use of mesenchymal stem cell and non-stem-cell-based therapy [62]. Emerging evidence implicates TRPV4 as an important contributor to pathophysiology of ALI and associated inflammation [33, 63, 64]. In this study, we synthesized a focused series of novel cannabidiol (CBD-**1**) derivatives as small-molecule inhibitors of TRPV4. CBD-**1** was isolated from *Cannabis sativa* and used as a precursor for the synthesis. Subsequently, the derivatives were synthesized by coupling of CBD-**1** with various secondary amines in the presence of formaldehyde, using methanol as the solvent. CBD-**1** has reported agonistic properties against TRPV4 [28]. We aimed to assess the TRPV4 antagonistic action of these compounds. To achieve this, we utilized A375 cell line to screen the compounds for TRPV4 inhibition, as this cell line exhibits relatively higher TRPV4 expression. We assessed calcium dynamics in these cells by monitoring the 340/380 ratio using Fura-2 AM to indicate Ca^2+^ influx. Our test compounds exhibited varying degrees of inhibition on TRPV4 mediated Ca^2+^ entry, with some significantly reducing the influx. Of all the compounds tested, CS-85 demonstrated the most significant results. We then conducted additional specificity and efficacy assays on CS-85, which were consistent with the screening data. Additionally, we assessed the TRPV4 inhibitory effects of CS-85 in BMDMs, and the results corroborated and confirmed the screening data. We performed an additional TRPV4 specificity assay in BMDMs where CS-85 was tested for its ability to block NFAT activation following ligand mediated TRPV4 activation. CS-85 significantly inhibited NFAT activation in response to this stimulation. We next assessed the anti-inflammatory potential of the CBD-**1** series in the RAW 264.7 cell line in an LPS induced inflammation assay, with NO release as the readout, where CS-85 demonstrated maximum inhibition. We further investigated the anti-inflammatory effects of CS-85 in RAW 264.7 cells by measuring additional parameters such as IL-6 and oxidative stress, both of which showed significant reduction. In the above assays, CS-85 demonstrated improved anti-inflammatory properties than its parent CBD-**1**.

When reassessed in BMDMs, both CS-85 and CBD-1 significantly reduced LPS induced oxidative stress and inflammation, as indicated by decreased NO, ROS, and IL-6 levels. However, unlike CS-85, CBD-1 exerted its anti-inflammatory effect independently of TRPV4. Given the marked decrease in LPS induced inflammatory parameters, we evaluated the phosphorylation of NF-κB, a key transcription factor in LPS induced inflammation, as well as iNOS expression. Both parameters showed significant reductions.

Before testing the *in vivo* efficacy of CS-85, we assessed its potential adverse effects in mice across a dose range of 30, 300, and 1500 µg/mouse to identify a safe and effective dose. However, no significant signs of adverse effect were observed. Notably, several preclinical studies have reported prophylactic administration of their test drugs as effective strategy for ALI treatment in their models [65, 66, 67]. Given the exploratory nature of this study, we performed prophylactic administration of CS-85 in LPS induced ALI model to test its efficacy. Multiple doses of CS-85 were tested to identify the most effective dose and its impact on various biochemical parameters such as IL-1β, IL-6, MPO, and total protein. On the basis of these findings, we identified 150 and 300 µg/mouse as the optimal doses for further ALI models.

Next, we assessed the potential of CS-85 against the hallmark features of ALI such as alveolar edema, lung vascular permeability, and intra-alveolar neutrophilic infiltration following LPS exposure in mice. We found that CS-85 significantly reduced edema formation and fluid accumulation, as well as lung vascular leakage in mice, as indicated by reduction in the wet-to-dry ratio and transendothelial albumin extravasation. Additionally, CS-85 led to a significant decrease in MPO activity in the lungs, suggesting reduced neutrophil infiltration into the alveoli. The compound also effectively preserved lung architecture, as shown in histological examination of lung sections.

Next, we sought to investigate whether pharmacological overactivation and/or overexpression of *Trpv4* in AMs can affect lung inflammation and ALI features. Toward this end, we adopted a two-pronged approach. Firstly, we induced TRPV4 overactivation by cotreatment of mice with GSK-A and LPS. Intriguingly, a significant overactivation was observed with this approach as indicated by comparative elevation of proinflammatory markers in the BAL fluid of mice. However, GSK-I and CS-85 effectively mitigated this response, resulting in a marked reduction in inflammatory markers, including MPO, IL-6, and total protein. Despite lower affinity for TRPV4 than GKS-I, CS-85 effectively attenuated lung injury owing to its dual inhibitory action. In our second approach, we overexpressed *Trpv4* in AMs via liposome-mediated delivery of Trpv4 plasmid DNA, followed by LPS treatment, as *Trpv4* expression is known to increase in inflammatory states [68, 69, 70]. We chose to target AMs, as the clinical outcomes in ARDS have been associated with transcriptional programming of these cells [71]. We observed that *Trpv4* overexpression and subsequent LPS exposure exacerbated ALI features in mice. However, pretreatment with CS-85 significantly reduced these features, which included alveolar edema and vascular leak, as indicated by a reduction in the wet-to-dry ratio and transendothelial albumin extravasation. These models strongly emphasize the role of TRPV4 in the progression and pathogenesis of ALI and highlight the critical need for its inhibition to prevent the escalation of ALI into ARDS. We then tested the efficacy of CS-85 in a therapeutic mouse model of ALI. The data corroborated with the findings of prophylactic mouse models of ALI. CS-85 demonstrated a marked inhibition of cytokines release and protein accumulation in the lung, indicating its efficacy to treat ALI after disease induction. To investigate the likely cellular target of CS-85, we depleted AMs from lungs using clodronate liposomes and determined lung injury following LPS challenge. We observed that CS-85 primarily targets AMs and significantly reduces cytokine levels in their presence. However, it also moderately interacts with other cell populations as indicated by its effect on cytokine release.

As demonstrated above, CS-85 reduced both NF-κB phosphorylation and NFAT activation. Additionally, we observed that CS-85 effectively reduced the activation of the NLRP3 inflammasome complex in BMDMs, as shown by its reduction in IL-1β release, in an NLRP3 inflammasome activation assay. We further investigated this finding by examining two critical sequential steps in NLRP3 inflammasome activation: caspase-1 activation and IL-1β cleavage, both of which were significantly reduced by CS-85. Considering the established role of NLRP3 inflammasome activation in the development of ALI, we analyzed the BAL fluid and lung homogenate from these mice for IL-1β and caspase-1 levels, observing that CS-85 significantly inhibited caspase-1 activation (p22 subunit) and IL-1β cleavage (p17 subunit). Standard compounds MCC950 and colchicine were also evaluated alongside CS-85 for their inhibitory potential, as both are established NLRP3 inflammasome inhibitors. The NLRP3 inflammasome activation assay is TRPV4 independent; a standard TRPV4 inhibitor was not included in this set of experiments. These findings corroborate our *in vitro* results, suggesting that CS-85 effectively suppresses the NLRP3 inflammasome activation in ALI. This dual-target mechanism positions CS-85 as a promising candidate for managing ALI, emphasizing the urgent need for TRPV4 based therapeutic strategies.

CS-85 did not demonstrate any significant side effects in our tested models. It did however demonstrate a minimal and statistically nonsignificant increase in MPO activity at 1500 µg/mouse dose, which is five times higher than its effective dose, 300 µg/mouse. Although, our findings showed CS-85 as an effective TRPV4 inhibitor, it minimally reduced TRPV1-mediated Ca^2+^ influx into the cells. Our study demonstrates the pivotal role of TRPV4 in modulating the inflammatory response during ALI and demonstrates the therapeutic potential of targeting this ion channel. We identified CS-85 as a promising small-molecule inhibitor that effectively mitigates the features of ALI in mice. The benefit of this cannabidiol-based TRPV4 inhibitor is its multi-target capability. CS-85 simultaneously inhibits TRPV4, TLR4, and NLRP3 inflammasome pathways, thereby enhancing efficacy and reducing compensatory resistance.

## Conclusions

Our study highlights the therapeutic potential of CS-85, a novel TRPV4 antagonist, in alleviating ALI. CS-85 treatment maintained pulmonary vascular integrity, reduced neutrophil influx, prevented lung flooding with protein-rich edema, and suppressed inflammation. It is effective against both pharmacologically induced and genetically exacerbated ALI. Mechanistically, CS-85 targeted TRPV4 and NLRP3-caspase-1 pathways to exert its anti-inflammatory effects. These findings position CS-85 as a promising and safe candidate for ALI therapy. The current study did not involve testing CS-85 via inhalation, oral, and intraperitoneal routes. However, we intend to test the efficacy of CS-85 through these routes and its efficacy in more severe disease models of ALI, which will advance the therapeutic development of CS-85 for clinical evaluation in ALI. Additionally, optimizing medicinal chemistry of this compound may enhance its therapeutic potential.

## Supplementary information

Additional file 1.

## Data Availability

All data generated or analyzed during this study are included in this published article (and its Supplementary Information files).

## Abbreviations

TRPV4: Transient receptor potential vanilloid 4
ALI: Acute lung injury
ARDS: Acute respiratory distress syndrome
VILI: Ventilator-induced lung injury
BAL: Bronchoalveolar lavage
ANOVA: Analysis of variance
BSA: Bovine serum albumin
DCFH-DA: Diacetyl dichlorofluorescein
DMSO: Dimethylsulfoxide
DMEM: Dulbecco’s modified eagle medium
FBS: Fetal bovine serum
LPS: Lipopolysaccharide
IL1-β: Interleukin-1 beta
IL-6: Interleukin 6
EB: Evans blue
L-NAME: Nitro-l-arginine methyl ester
MTT: 3-(4,5-Dimethylthiazol-2-yl)-2,5-diphenyltetrazolium bromide
NO: Nitric oxide
i-NOS: Inducible nitric oxide synthase
NF-κB: Nuclear factor-κB
PBS: Phosphate-buffered saline
TNF-α: Tumor necrosis factor-alpha
TLR4: Toll-like receptors
PEG: Polyethylene glycol
ATP: Adenosine triphosphate
MCC950: *N*-(1,2,3,5,6,7-hexahydro-*s*-indacen-4-ylcarbamoyl)-4-(2-hydroxy-2-propanyl)-2-furansulfonamide
EDTA: Ethylenediaminetetraacetic acid
PMSF: Phenylmethylsulfonyl fluoride
NaCl: Sodium chloride
H_2_O_2_: Hydrogen peroxide
RPMI: Roswell Park Memorial Institute Medium
GSK-A: GSK1016790A
GSK-I: GSK2193874
PCR: Polymerase chain reaction
CVR: Carvacrol
PBC: Probenecid
