# Supplementary material for: Discovery of a novel cannabidiol-derived transient receptor potential vanilloid 4 inhibitor to reduce pulmonary edema and lung vascular permeability in mice
Source: Cell Mol Biol Lett. 2026 May 17;31:117. doi: 10.1186/s11658-026-00939-3 (PMC13374314; doi:10.1186/s11658-026-00939-3)
Supplement: Supplementary file 1 — Additional file 1. [file 11658_2026_939_MOESM1_ESM.pdf]

Supporting Information

Discovery of a novel Cannabidiol-Based TRPV4 Inhibitor to Reduce Pulmonary Edema and lung vascular permeability in mice

Yassir Arfath†[1,3], Pankaj Singh Cham†[2,3], Tusharika Kotra[1,3], Rahila Akhter[1,3], Sumeer Ahmed[2], Mandeep Kour[2], Sheikh Tasduq Abdullah[1,3], Parvinder P Singh*[2.3], Sheikh Rayees*[1,3] Zabeer Ahmed*[1,3]

[1]Pharmacology Division, CSIR-Indian Institute of Integrative Medicine, Jammu, India
[2]Natural Products and Medicinal Chemistry Division, CSIR-Indian Institute of Integrative Medicine, Jammu, India
[3]Academy of Scientific and Innovative Research (AcSIR), Ghaziabad, India

† The authors contributed equally

Correspondence:

Zabeer Ahmed
Director
CSIR-Indian Institute of Integrative Medicine, Canal Road, Jammu-180001
Email: zahmed.iiim@csir.res.in

Sheikh Rayees
Ramalingaswami Fellow, & Assistant Professors AcSIR,
CSIR-Indian Institute of Integrative Medicine, Canal Road, Jammu-180001
Email: rayees.sheikh@gmail.com, rayeesh.iiim@csir.res.in

Parvinder P Singh
Principal Scientist
CSIR-Indian Institute of Integrative Medicine, Canal Road, Jammu-180001
Email: ppsingh.iiim@csir.res.in , ppsingh@iiim.ac.in

Table of Contents

General information and materials……………………..………………………….…….……S3
Experimental details.……….……...………………...……….……………………...…S3-S4
Figure S1……………………………………………………….…………………………..…S4
HPLC analysis……………………………………..………………………….………………S5
Table S1……………………………………………………………………………………..…..S5
Figure S2………………………………………………………………………………………….S6
Figure S3………………………………………………………………………………………….S7
Figure S4…………………………………………………………………………….……………S8
General procedure for the synthesis of novel CBD analogs (**4a,a'-o**) of CBD **1**….............S9
Table S2……………………………………………………………………………………..….S9
Spectral data of cannabidiol derivatives **………………...…………………….....……..**S10-S15
Figure S5-S68: Spectral copies of ^{1}H-NMR,^{13}C-NMR,DEPT,HRMS of **4a,a'-o ….....**S16-S47
Figure S69: Molcular Docking studies………………….……………………..........…S48-S54
Table S3………………………………………………………………………………………S55
Table S4: Solubility studies of **4a**, **4d**, **4j** (**CS-85**), **4k** and CBD **1** *(in vitro)*…………..….S56
Table S5………………………………………………………………………………….…S57
Figure S70……………………………………………………………………………………..S58
Figure S71……………………………………………………………………………………S58
Figure S72……………………………………………………………………………………S59
Figure S73 …………...……………………………………………………………………..S59
Figure S74…………...……………………………………………………………………...S60
Figure S75…………...……………………………………………………………………...S60
Table S6.………...………………………………………………………………................S61
Figure S76…………...……………………………………………………………………...S62
Figure S77…………...……………………………………………………………………...S62
References………………………………………………………………………..……....S63

General information and materials

Chemistry

All reactions were carried out at room temperature under a nitrogen atmosphere and monitored using pre-coated silica gel TLC plates (Merck, 60 F254, 20 × 20 cm). TLC plates were visualized under UV light at 254 nm or by charring with visualizing agents such as anisaldehyde, ninhydrin, or Dragendorff's reagent. A Büchi rotavapor was used for concentrating organic solvents. Compounds were purified using column chromatography with silica gel (100–200 mesh). The ^{1}H NMR and ^{13}C NMR and DEPT NMR spectra were recorded on a Bruker 400 MHz NMR spectrometer. Chemical shift data for protons are reported in parts per million (ppm) relative to tetramethylsilane (TMS) and referenced to the residual proton signal of the NMR solvent (CDCl3: δ 7.26 or other solvents as indicated). NMR spectra were processed using MestReNova software, with coupling constants (J) used in Hz. High-resolution mass spectra were obtained using a Q-TOF-LCMS (Waters) spectrometer, and/or LCMS analysis was performed on a Shimadzu triple quadrupole mass spectrometer with electrospray ionization. HPLC analysis was conducted on a Shimadzu UFLC system equipped with a quaternary pump, autosampler, column compartment, and diode array detector. Chromatographic separation utilized a Purospher STAR RP-18 endcapped column (250 × 4.6 mm, 5 µm).

Experimental details

Isolation of phytocannabinoids

The grounded plant material (aerial part, 15 kg) was mixed with ethanol in a ratio of 1:8 (w/v) in a closed extraction vessel and stirred for a period of 8h at room temperature. The ethanolic extract was filtered. The residue was extracted two more times, filtered and the combined ethanolic extract was dried under vacuum using a rota-evaporator at 70 °C to give 2.3 kg of extract (E.V.: 15.3% w/w). The CBD **1** content in the residual plant material and ethanolic extract was found to be 0.58% and 4% respectively (Figure S3). The 2.3 kg of extract was passed through silica (100 – 200 mess size). It was eluted with ethyl acetate in hexane with varying concentrations as 1 – 5% (v/v) followed by methanol. A total of five fractions were collected and evaporated to give 20 g, 67 g, 21 g, 18 g, and finally 2 kg (methanolic fraction) as semi-purified fractionates. Further, the 67 g (second fraction) of semi-purified extract was re-loaded on a silica column (100 – 200 mesh size) and eluted with ethyl acetate and hexane (1% – 100%) to obtain four fractions. The fractions were evaporated

to dryness to give 9 g, 30 g, 7 g, and 21 g of enriched extracts. The second fraction (30 g) was identified as CBD **1** enriched, whereas the third fraction was found to have THC as a major constituent. The CBD **1** enriched extract (30 g) was further purified through column chromatography using ethyl acetate/ hexane system to give 14 g of CBD **1** with >95% purity (Figure S4). The purity of CBD **1** was estimated by HPLC (Shimadzu).

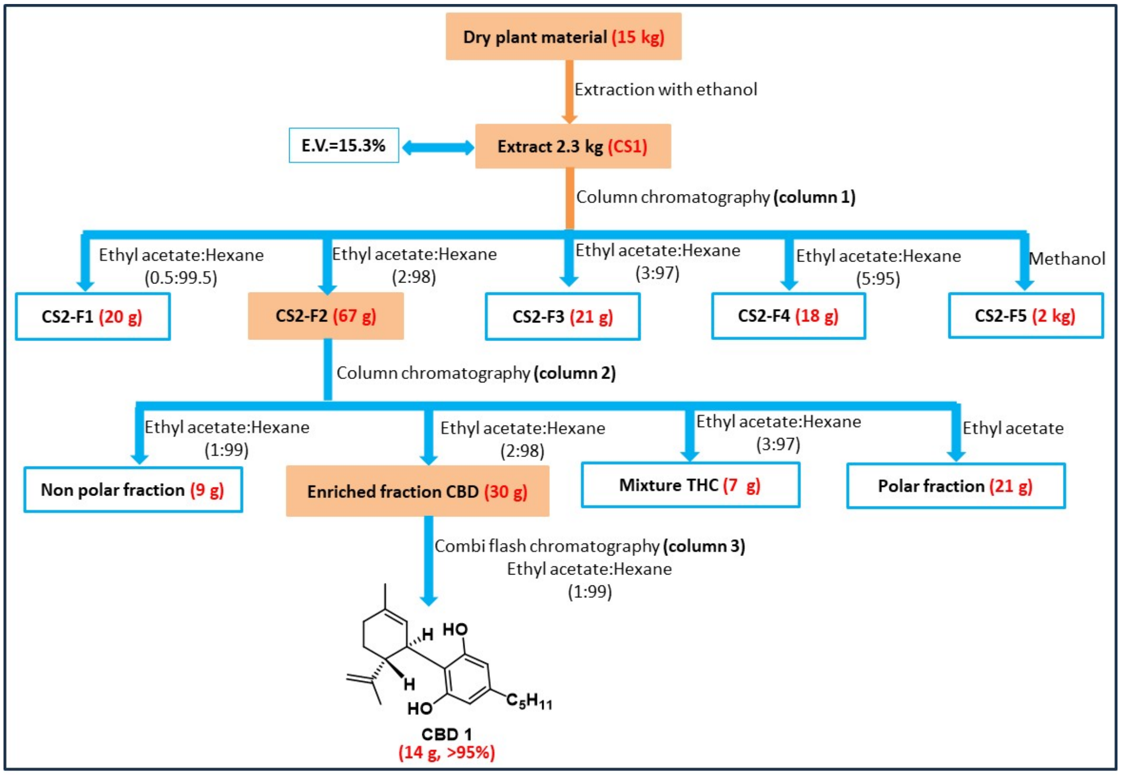

Figure S1. Isolation of cannabidiol (CBD **1**) from *Cannabis sativa*

HPLC analysis

Test Sample Preparation. A 50 mg portion of dried cannabis plant material was extracted with 2.5 ml of ethanol by placing the mixture in a shaker at 200 rpm for 15 minutes at 30 °C. The mixture was then centrifuged at 4000 rpm for 10 minutes, and the supernatant was collected. This extraction process was repeated two times more, first with 1.5 ml of ethanol and then with 1 ml of ethanol, resulting in a final concentration of 50 mg in 5 ml (equivalent to 10 mg/mL). It was estimated for the presence of CBD **1** through the HPLC system.

Standard Preparation. A stock solution of CBD **1** was prepared at a concentration of 10 mg/mL in ethanol. From this stock solution, a standard solution of 0.1 mg/ml was prepared and serially diluted to generate a range of standard concentrations. The calibration curve for CBD **1** was constructed over a concentration range of 1 μg/10 μl to 0.0625 μg/10 μl and was used for quantification purposes.

The mobile phase consisted of a buffer solution (0.1% formic acid in water) and 0.1% formic acid in acetonitrile. The analysis was performed at a flow rate of 1 ml/minute with the column maintained at 25 °C, and detection was carried out at 210 nm. A sample volume of 10 μl was injected into the HPLC system (Shimadzu), and the assay was conducted using a gradient method with a total run time of 40 minutes (Table S1).

Table S1. The gradient method for quantification purposes[1]

**Gradient method**		
**Time (min)**	**Solvent A(%)**	**Solvent B(%)**
**0**	30	70
**20**	2	98
**22**	2	98
**30**	30	70
**35**	30	70
**40**	30	70

"Reproduced [Reprinted] with permission from Ref. [Cham, P. S.; Deepika; Bhat, R.; Raina, D.; Manhas, D.; Kotwal, P.; Mindala, D. P.; Pandey, N.; Ghosh, A.; Saran, S.; Nandi, U.; Khan, I. A.; Singh, P. P. Exploring the Antibacterial Potential of Semisynthetic Phytocannabinoid: Tetrahydrocannabidiol (THCBD) as a Potential Antibacterial Agent against Sensitive and Resistant Strains of Staphylococcus aureus. ACS Infectious Diseases 2023]. Copyright [2023] [ACS Journal]."

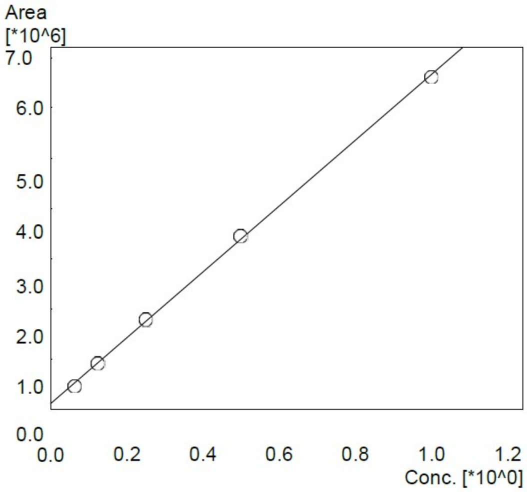

S. No.	Conc. (Ratio)	Mean Area	Area
1	0.0625	474580	474580
2	0.125	923970	923970
3	0.25	1789335	1789335
4	0.5	3454980	3454980
5	1	6619314	6619314

Name: CBD
Function: f(x)=6.53798e+006*x+118969
Rr1=0.9997957 Rr2=0.9995914
RSS=1.015530e+010

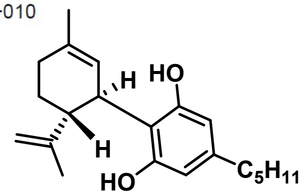

Figure S2. Calibration curve of cannabidiol (CBD **1**)[1] "Reproduced [Reprinted] with permission from Ref. [Cham, P. S.; Deepika; Bhat, R.; Raina, D.; Manhas, D.; Kotwal, P.; Mindala, D. P.; Pandey, N.; Ghosh, A.; Saran, S.; Nandi, U.; Khan, I. A.; Singh, P. P. Exploring the Antibacterial Potential of Semisynthetic Phytocannabinoid: Tetrahydrocannabidiol (THCBD) as a Potential Antibacterial Agent against Sensitive and Resistant Strains of *Staphylococcus aureus*. ACS Infectious Diseases 2023]. Copyright [2023] [ACS Journal]."

<Sample Information>

Sample Name	: Plant material
Sample ID	: Plant material
Data Filename	: Plant material
Method Filename	: gradient 70-98.lcm
Batch Filename	: E2
Vial #	: 1-2
Injection Volume	: 10 uL
Date Acquired	: 26-02-2021 23:57:14
Date Processed	: 08-08-2023 15:01:16

Sample Type	: Unknown(QA/QC)
Level	: 1
Acquired by	: System Administrator
Processed by	: System Administrator

<Chromatogram>

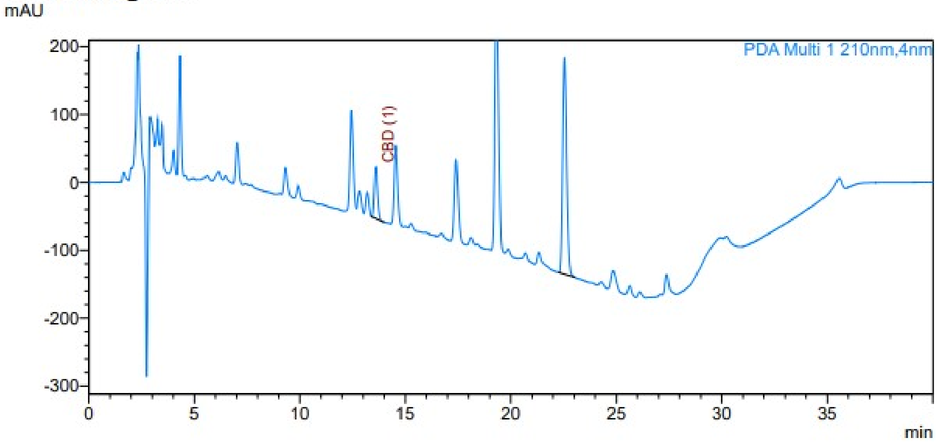

Data File Name	Sample Name	Sample ID	Ret. Time	Area	Height	Conc.
CBD **1**, 1	CBD **1**, 1	CBD **1**, 1	13.716	493043	50096	0.057
CBD **1**, 2	CBD **1**, 2	CBD **1**, 2	13.697	949159	96845	0.125
CBD **1**, 3	CBD **1**, 3	CBD **1**, 3	13.667	1811138	183695	0.254
CBD **1**, 4	CBD **1**, 4	CBD **1**, 4	13.683	3485775	350739	0.504
CBD **1**, 5	CBD **1**, 5	CBD **1**, 5	13.710	6798726	671547	0.998
PLANT M 1	PLANT M 1	PLANT M 1	13.596	832572	76296	0.608
PLANT M 2	PLANT M 2	PLANT M 2	13.578	857287	78427	0.612
PLANT M 3	PLANT M 3	PLANT M 3	13.591	863278	79086	0.512
Average			13.655	2011372	198341	0.577
%RSD			0.419	107.302	108.326	71.011
Maximum			13.716	6798726	671547	0.998
Minimum			13.578	493043	50096	0.057
SD			0.057	2158238	214855	0.322

Figure S3. HPLC chromatogram of plant material[2] “Reproduced [Reprinted] with permission from Ref. [Cham, P. S.; Deepika; Bhat, R.; Raina, D.; Manhas, D.; Kotwal, P.; Mindala, D. P.; Pandey, N.; Ghosh, A.; Saran, S.; Nandi, U.; Khan, I. A.; Singh, P. P. Exploring the Antibacterial Potential of Semisynthetic Phytocannabinoid: Tetrahydrocannabidiol (THCBD) as a Potential Antibacterial Agent against Sensitive and Resistant Strains of *Staphylococcus aureus*. ACS Infectious Diseases 2023]. Copyright [2023] [ACS Journal].”

<Sample Information>

Sample Name	: CBD 1
Sample ID	: CBD 1
Data Filename	: CBD 1
Method Filename	: gradient method.lcm
Batch Filename	: CBD 1
Vial #	: 1-8
Injection Volume	: 10 uL
Date Acquired	: 20-08-2021 04:04:31
Date Processed	: 22-11-2024 15:00:59

Sample Type	: Unknown
Acquired by	: System Administrator
Processed by	: System Administrator

<Chromatogram>

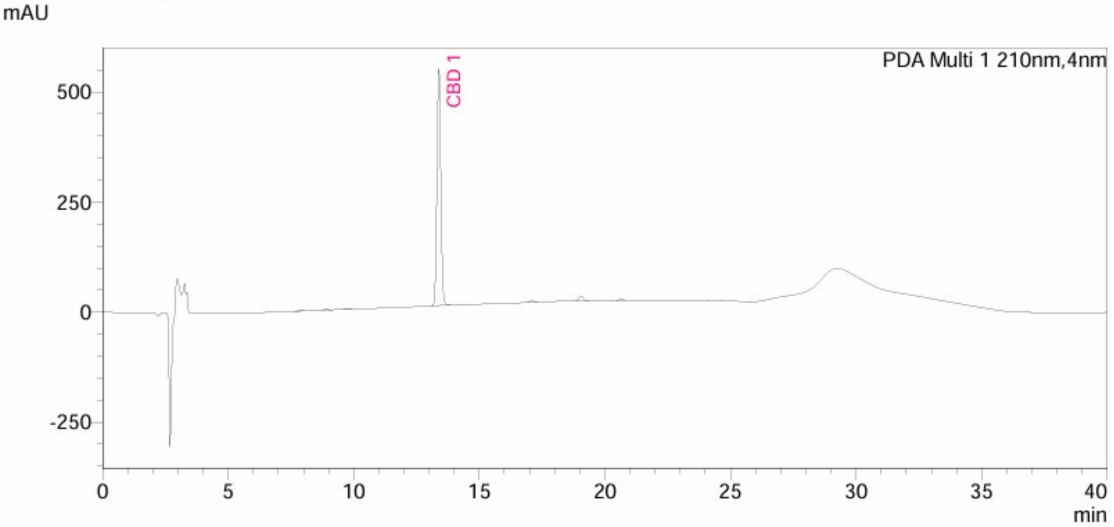

<Peak Table>

PDA Ch1 210nm

Peak#	Ret. Time	Area	Height	Area%
1	7.812	13122	1439	0.215
2	8.906	34381	3670	0.563
3	9.750	17576	1776	0.288
4	13.386	5847363	537452	95.789
5	17.109	45980	4234	0.753
6	19.047	116114	10443	1.902
7	20.647	29874	2823	0.489
Total		6104412	561837	100.000

Figure S4. HPLC chromatogram of cannabidiol (CBD **1**)

General procedure for the synthesis of novel CBD analogs (4a-o and 4a′-o′) of CBD 1.

To a solution of cannabidiol (**1**, 120 mg, 0.382 mmol) in methanol was added formaldehyde solution (**3**, 37%, 83 – 151 μl) followed by secondary amine {**2**, 0.458-1.146 mmol, (1.2-3)eq.}. The reaction mixture was stirred at room temperature (rt) for 15 hours. The reaction mixture was monitored by TLC and observed for consumption of reactant. After the completion of the reaction, the reaction mixture was extracted with ethyl acetate (2 × 200 ml volume) and water. The organic layer was collected and concentrated in vacuo on rota evaporator. The compounds were purified through column chromatography and eluted with ethyl acetate and hexane or methanol and dichloroform to get the 2'-{heterocyclyl (aryl/alkyl) methyl}-cannabidiol (Table S2).

Table S2. Information of the synthesis of CBD analogs

S. No.	Secondary amines, 2: mg, mmol (eq.)	37% HCHO, 3: μL(eq.)	4, Compounds (% yield)
1.	**2a** (39.8, 0.458, 1.2eq.)	83 μL (2.2 eq.)	**4a** (Mono: 110 mg, 83.9%)
2.	**2a** (99.7, 1.146, 3 eq.)	151 μL(4 eq.)	**4a & 4a′{(**Mono: 101 mg, 64.3%) (Di: 45 mg, 28.6%)}
3.	**2b** (38.9, 0.458, 1.2 eq.)	83 μL(2.2 eq.)	**4b** (Mono: 102 mg, 64.9%)
4.	**2c** (58.2, 0.458, 1.2 eq.)	83 μL(2.2eq.)	**4c** (Mono: 50 mg, 28.9%)
5.	**2d** (46.2, 0.0.458, 1.2eq.)	83 μL(2.2 eq.)	**4d** (Mono: 98 mg, 60.1%)
6.	**2e** (73.8, 0.458, 1.2 eq.)	83 μL(2.2 eq.)	**4e** (Mono: 140 mg, 75.2%)
7.	**2f** (70.1, 0.458, 1.2 eq.)	83 μL(2.2 eq.)	**4f** (Mono: 145 mg, 79.2%)
8.	**2g** (80.1, 0.458, 1.2 eq.)	83 μL(2.2 eq.)	**4g** (Mono: 140 mg, 73.2%)
9.	**2h** (45.8, 0.458, 1.2 eq.)	83 μL(2.2eq.)	**4h** (Mono: 140 mg, 86.4%)
10.	**2i** (52.2, 0.458, 1.2 eq.)	83 μL(2.2 eq.)	**4i** (Mono: 150 mg, 89.2%)
11.	**2j** (59.5, 0.458, 1.2 eq.)	83 μL(2.2eq.)	**4j** (Mono: 90 mg, 51.7%)
12.	**2k** (58.6, 0.458, 1.2 eq.)	83 μL(2.2eq.)	**4k** (Mono: 130 mg, 75%)
13.	**2l** (74.1, 0.458, 1.2 eq.)	83 μL(2.2eq.)	**4l** (Mono: 130 mg, 69.8%)
14.	**2m** (57.7, 0.458, 1.2 eq.)	83 μL(2.2 eq.)	**4m** (Mono: 130 mg, 75.5%)
15.	**2n** (89.7, 0.458, 1.2 eq.)	83 μL(2.2eq.)	**4n** (Mono: 101 mg, 50.7%)
16.	**2o** (75.2, 0.458, 1.2 eq.)	83 μL(2.2 eq.)	**4o** (Mono: 105 mg, 56.1%)
17.	**2p** (45.8, 0.458, 1.2 eq.)	83 μL(2.2 eq.)	**4p** (Mono: 168 mg, 61%)

Spectral data of cannabidiol derivatives

2'-(morpholin-1-ylmethyl)-cannabidiol (4a). ^{1}H NMR (400 MHz, $CDCl_3$) δ 11.21 (bs, 1H,

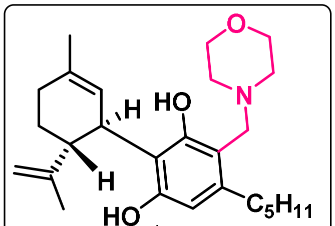

4a

OH), 6.16 (s, 1H), 5.96 (s, 1H), 5.61 (s, 1H), 4.34 (d, J = 23.5 Hz, 2H), 4.02 (d, J = 8.4 Hz, 1H), 3.79 – 3.53 (m, 6H), 2.86 – 2.36 (m, 7H), 2.23 – 2.04 (m, 2H), 1.80 – 1.74 (s, 5H), 1.70 (s, 3H), 1.47 – 1.42 (m, 2H), 1.33 – 1.27 (m, 4H), 0.90 – 0.86 (m, 3H); ^{13}C {^{1}HNMR (101 MHz, $CDCl_3$) δ 157.2, 155.6, 148.5, 140.7, 139.8, 125.1, 115.1, 111.0, 110.3, 109.0, 67.2, 56.9, 52.8, 47.3, 36.0, 33.8, 32.1, 31.3, 30.8, 28.4, 24.2, 23.0, 19.5, 14.5; $[\alpha]_D^{20}$ = −142 (c = 1.0, MeOH); HRMS (ESI-TOF) m/z: calcd for $C_{26}H_{40}NO_3$ 414.3008 $[M+H]^+$, found 414.2999.

2',4'-{bis-(morpholin-1-ylmethyl)}-cannabidiol (4a'). ^{1}H NMR (400 MHz, $CDCl_3$) δ 10.9

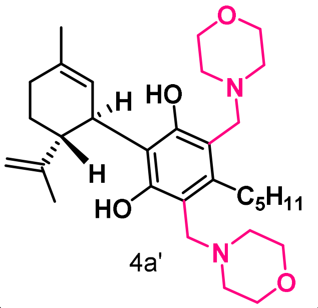

4a'

(bs, 2H, OH), 5.35 (s, 1H), 4.34 (d, J = 26 Hz, 2H), 4.02 (d, J = 8.4 Hz, 1H), 3.71 (s, 12H), 3.19 – 3.12 (m, 1H), 2.48 – 2.27 (m, 10H), 2.23 – 1.98 (m, 2H), 1.78 – 1.74 (m, 2H), 1.70 (s, 3H), 1.56 (s, 3H), 1.37 – 1.31 (m, 6H), 0.92 (t, J = 6.8 Hz, 3H); ^{13}C {^{1}H} NMR (101 MHz, $CDCl_3$) δ 157.2, 150.9, 137.8, 131.9, 126.6, 117.0, 109.2, 67.1, 57.4, 52.9, 44.9, 36.9, 32.4, 31.2, 31.1, 30.1, 29.3, 24.0, 22.9, 19.3, 14.5; $[\alpha]_D^{20}$ = −96.6 (c = 1.0, MeOH); HRMS (ESI-TOF) m/z: calcd for $C_{31}H_{49}N_2O_4$ 513.3692 $[M+H]^+$, found 513.3689.

2'-(piperidin-1-ylmethyl)-cannabidiol (4b). ^{1}H NMR (400 MHz, $CDCl_3$) δ 6.13 (s, 1H),

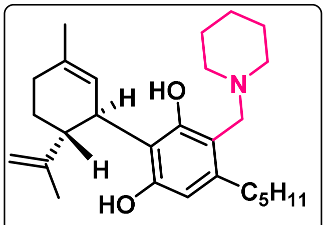

4b

5.62 (s, 1H), 4.38 (d, J = 43.2 Hz, 2H), 4.03 (d, J = 8.4 Hz, 1H), 3.56 (dd, J = 51.2, 13.6 Hz, 2H), 3.05 – 2.54 (m, 2H), 2.48 – 2.38 (m, 3H), 2.27 – 2.20 (m, 4H), 1.81 – 1.77 (m, 5H), 1.71 (s, 3H), 1.68 – 1.41 (m, 8H), 1.33 – 1.28 (m, 4H), 0.88 (t, J = 6.8 Hz, 3H); ^{13}C {^{1}H} NMR (101 MHz, $CDCl_3$) δ 157.8, 155.3, 148.4, 140.3, 139.6, 125.4, 115.0, 111.2, 111.0, 108.5, 57.4, 53.9, 47.3, 36.1, 33.8, 32.1, 31.2, 30.8, 28.5, 26.3, 24.6, 24.1, 23.0, 19.5, 14.5; $[\alpha]_D^{20}$ = −118 (c = 1.0, MeOH); HRMS (ESI-TOF) m/z: calcd for $C_{27}H_{42}NO_2$ 412.3216 $[M+H]^+$, found 412.3216.

2'-{(4-isopropylpiperidin-1-yl)methyl}-cannabidiol (4c). ^{1}H NMR (400 MHz, $CDCl_3$) δ 6.13 (s, 1H), 5.93 (bs, 1H, OH), 5.62 (s, 1H), 4.39 (d, J = 41.2 Hz, 2H), 4.02 (d, J = 9.6 Hz, 1H), 3.56 (dd, J = 54.4, 13.6 Hz, 2H), 3.03 – 2.86 (m, 2H), 2.47 – 2.35 (m, 3H), 2.25 – 1.97 (m, 4H), 1.81 – 1.77 (m, 5H), 1.70 (s, 3H), 1.67 – 1.59 (m, 2H), 1.48 – 1.39 (m, 3H), 1.33 – 1.18 (m, 6H), 1.08 -1.02 (m, 1H), 0.90 – 0.86 (m, 9H); ^{13}C {^1H} NMR (101 MHz, $CDCl_3$) δ 157.8, 155.3, 148.4, 140.3, 139.6, 125.4, 115.0, 111.3, 111.0, 108.5, 57.0, 54.4, 52.6, 47.3, 42.7, 36.2, 33.8, 32.7, 32.2, 31.2, 30.9, 29.8, 29.6, 28.5, 24.2, 23.0, 20.3, 20.2, 19.5, 14.5; $[\alpha]_D^{20}$ = −84.6 (c = 1.0, MeOH); HRMS (ESI-TOF) m/z: calcd for $C_{30}H_{48}NO_2$ 454.3685 $[M+H]^+$, found 454.3680.

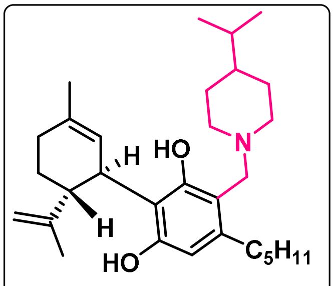

4c

2'-{(4-hydroxypiperidin-1-yl)methyl}-cannabidiol (4d). ^{1}H NMR (400 MHz, $CDCl_3$) δ 6.13 (s, 1H), 5.94 (bs, 1H, OH), 5.60 (s, 1H), 4.37 (d, J = 43.2 Hz, 2H), 4.01 (s, 1H), 3.75 – 3.49 (m, 3H), 2.96 – 2.76 (m, 2H), 2.45 – 2.36 (m, 3H), 2.26 – 1.98 (m, 4H), 1.93 – 1.83 (m, 2H), 1.80 – 1.74 (m, 5H), 1.70 (s, 3H), 1.65 – 1.54 (m, 2H), 1.47 – 1.39 (m, 2H), 1.31 – 1.25 (m, 4H), 0.87 (t, J = 6.8 Hz, 3H); ^{13}C {^1H} NMR (101 MHz, $CDCl_3$) δ 157.5, 155.3, 148.4, 140.3, 139.7, 125.2, 115.1, 111.0, 108.7, 108.3, 56.6, 49.9, 47.2, 36.1, 34.8, 33.8, 32.1, 31.2, 30.8, 28.4, 24.1, 22.9, 19.5, 14.5; $[\alpha]_D^{20}$ = −96 (c = 1.0, MeOH); HRMS (ESI-TOF) m/z: calcd for $C_{27}H_{42}NO_3$ 428.3165 $[M+H]^+$, found 428.3160.

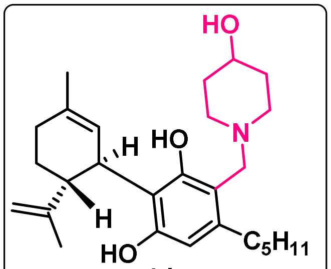

4d

2'-{(4-phenylpiperidin-1-yl)methyl}z-cannabidiol (4e). ^{1}H NMR (400 MHz, $CDCl_3$) δ 7.40 – 7.27 (m, 5H), 6.22 (s, 1H), 6.01 (bs, 1H, OH), 5.70 (s, 1H), 5.19 (bs, 1H, OH), 4.45 (d, J = 35.6 Hz, 2H), 3.13 (s, 1H), 3.70 (dd, J = 51.6, 13.6 Hz, 2H), 3.21 – 3.04 (m, 2H), 2.67 – 2.60 (m, 1H), 2.55 – 2.44 (m, 3H), 2.33 – 2.08 (m, 4H), 1.99 – 1.91 (m, 2H), 1.88 – 1.84 (m, 5H), 1.79 (s, 3H), 1.74 – 1.66 (m, 2H), 1.55 – 1.49 (m, 2H), 1.39 – 1.32 (m, 4H), 0.96 (t, J = 6.8 Hz, 3H); ^{13}C {^1H} NMR (101 MHz, $CDCl_3$) δ 157.7, 155.4, 148.4, 146.1, 140.4, 139.7, 128.9, 127.2, 126.8, 125.3, 115.1, 111.1, 108.7, 57.0, 54.4, 52.6, 47.3, 42.8, 40.1, 36.1, 33.9, 33.7, 32.2, 31.2, 30.8, 28.4, 24.2, 23.0, 19.6, 14.5; $[\alpha]_D^{20}$ = −46.3 (c = 1.0, MeOH); HRMS (ESI-TOF) m/z: calcd for $C_{33}H_{46}NO_2$ 488.3529 $[M+H]^+$, found 488.3527.

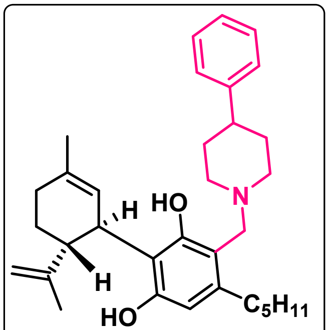

4e

2'-[{4-(trifluoromethyl)piperidin-1-yl}methyl]-cannabidiol (4f). ^{1}H NMR (400 MHz, CDCl$_3$) δ 11.19 (bs, 1H, OH), 6.16 (s, 1H), 5.97 (bs, 1H, OH), 5.61 (s, 1H), 4.38 (d, J = 40 Hz, 2H), 4.02 (s, 1H), 3.60 (dd, J = 13.6, 56.8, 2H), 3.12 – 2.95 (m, 2H), 2.46 – 2.35 (m, 3H), 2.25 – 2.05 (m, 4H), 1.96 -1.81 (m, 4H), 1.80 – 1.75 (m, 5H), 1.70 (s, 3H), 1.60 – 1.52 (m, 1H), 1.46 – 1.41 (m, 2H), 1.32 – 1.26 (m, 4H), 0.88 (t, J = 6.8 Hz, 3H); ^{13}C {^{1}H} NMR (101 MHz, CDCl$_3$) δ 157.4, 155.5, 148.5, 140.4, 139.8, 131.8, 129.0, 126.2, 125.1, 115.2, 111.0, 110.6, 108.9, 56.7, 52.5, 50.8, 40.6 (q, J = 27.2) , 36.1, 33.8, 32.1, 31.2, 30.8, 28.4, 25.2, 24.9, 24.1, 23.0, 19.5, 14.5; $[\alpha]_D^{20}$ = −75 (c = 1.0, MeOH); HRMS (ESI-TOF) m/z: calcd for $C_{28}H_{41}F_3NO_2$ 480.3089 [M+H]$^+$, found 480.3082.

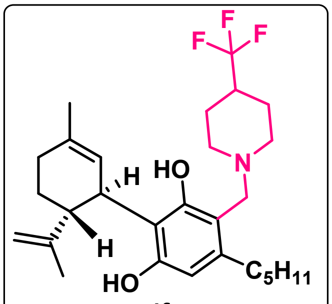

4f

2'-{(4-benzylpiperidin-1-yl)methyl}-cannabidiol (4g). ^{1}H NMR (400 MHz, CDCl$_3$) δ 7.30 – 7.13 (m, 5H), 6.13 (s, 1H), 5.93 (bs, 1H, OH), 5.62 (s, 1H), 4.39 (d, J = 42.4 Hz, 2H), 4.02 (s, 1H), 3.56 (dd, J = 13.6, 54 Hz, 2H), 2.97 – 2.83 (m, 2H), 2.60 – 2.53 (m, 2H), 2.46 – 2.34 (m, 3H), 2.25 – 2.04 (m, 4H), 1.82 – 1.78 (m, 6H), 1.68 – 1.58 (m, 7H), 1.47 – 1.40 (m, 2H), 1.32 – 1.26 (m, 4H), 0.87 (t, J = 6.8 Hz, 3H); ^{13}C {^{1}H} NMR (101 MHz, CDCl$_3$) δ 157.7, 155.3, 148.4, 140.8, 140.3, 139.6, 129.5, 128.7, 126.3, 125.3, 115.0, 111.1, 111.0, 108.6, 57.0, 52.3, 47.3, 43.4, 38.2, 36.1, 33.8, 32.5, 32.1, 31.2, 30.8, 30.1, 28.5, 24.2, 23.0, 19.6, 14.5; $[\alpha]_D^{20}$ = −82.6 (c = 1.0, MeOH); HRMS (ESI-TOF) m/z: calcd for $C_{34}H_{48}NO_2$ 502.3685 [M+H]$^+$, found 502.3673.

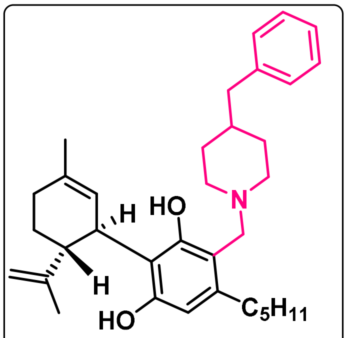

4g

2'-{(4-methylpiperazin-1-yl)methyl}-cannabidiol (4h). ^{1}H NMR (400 MHz, CDCl$_3$) δ 11.34 (bs, 1H, OH), 6.14 (s, 1H), 5.97 (bs, 1H, OH), 5.60 (s, 1H), 4.35 (d, J = 32 Hz, 2H), 4.0 (s, 1H), 3.60 (dd, J= 13.6, 53.2 Hz, 2H), 3.36 – 2.52 (m, 6H), 2.44 – 2.38 (m, 3H), 2.30 (s, 3H), 2.24 – 2.03 (m, 4H), 1.79 – 1.77 (m, 5H), 1.70 (s, 3H), 1.46 – 1.40 (m, 2H), 1.31 – 1.25 (m, 4H), 0.86 (t, J = 6.8 Hz, 3H); ^{13}C {^{1}H} NMR (101 MHz, CDCl$_3$) δ 157.4, 155.4, 148.4, 140.5, 139.7, 125.2, 115.1, 111.0, 110.7, 108.8, 56.6, 55.4, 52.6, 47.2, 46.3, 36.1, 33.8, 32.1, 31.2, 30.8, 28.4, 24.2, 23.0, 19.5, 14.5; $[\alpha]_D^{20}$ = −85.6 (c = 1.0, MeOH); HRMS (ESI-TOF) m/z: calcd for $C_{27}H_{43}N_2O_2$ 427.3325 [M+H]$^+$, found 427.3324.

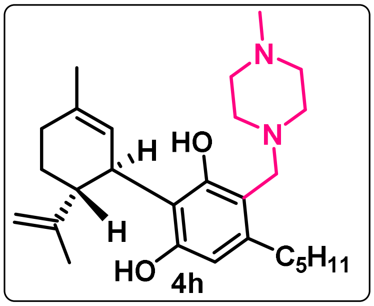

4h

2'-{(4-ethylpiperazin-1-yl)methyl}-cannabidiol (4i). ^{1}H NMR (400 MHz, $CDCl_3$) δ 6.14 (s, 1H), 5.95 (bs, 1H, OH), 5.60 (s, 1H), 4.36 (d, J = 31.2 Hz, 2H), 4.01 (d, J = 8.4 Hz, 1H), 3.60 (dd, J = 52.4, 13.6 Hz, 2H), 3.39 – 2.83 (m, 4H), 2.47 – 2.35 (m, 7H), 2.23 – 1.99 (m, 4H), 1.80 – 1.76 (m, 5H), 1.69 (s, 3H), 1.47 – 1.40 (m, 2H), 1.31 – 1.24 (m, 4H), 1.09 (t, J = 7.2 Hz, 3H), 0.86 (t, J = 6.8 Hz, 3H); ^{13}C {^{1}H} NMR (101 MHz, $CDCl_3$) δ 157.4, 155.4, 148.4, 140.5, 139.7, 125.2, 115.0, 111.0, 110.7, 108.8, 56.6, 53.0, 52.6, 49.8, 47.2, 36.1, 33.8, 32.1, 31.3, 30.8, 28.4, 24.2, 23.0, 19.5, 14.5, 12.4; $[\alpha]_D^{20}$ = −93.3 (c = 1.0, MeOH); HRMS (ESI-TOF) m/z: calcd for $C_{28}H_{45}N_2O_2$ 441.3481 $[M+H]^+$, found 441.3481.

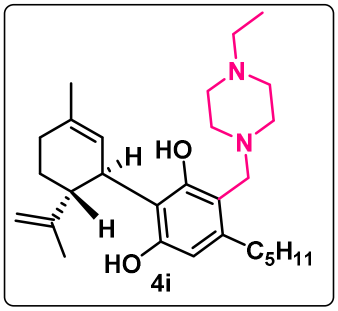

2'-[{4-(2-hydroxyethyl)piperazin-1-yl}methyl]-cannabidiol (4j). ^{1}H NMR (400 MHz, $CDCl_3$) δ 6.14 (s, 1H), 5.94 (s, 1H), 5.59 (s, 1H), 4.35 (d, J = 28 Hz, 2H), 4.00 (d, J = 10.4 Hz, 1H), 3.68 – 3.52 (m, 4H), 3.35 – 2.72 (m, 4H), 2.57 – 2.54 (m, 3H), 2.45 – 2.35 (m, 4H), 2.29 – 2.03 (m, 4H), 1.79 – 1.73 (m, 5H), 1.68 (s, 3H), 1.47 – 1.40 (m, 2H), 1.33 – 1.24 (m, 4H), 0.87 (t, J = 6.8 Hz, 3H); ^{13}C NMR (101 MHz, $CDCl_3$) δ 157.3, 155.4, 148.4, 140.5, 139.7, 125.1, 115.0, 110.9, 110.6, 108.9, 59.5, 58.2, 56.5, 53.1, 47.2, 36.0, 33.8, 32.1, 31.2, 30.8, 30.1, 28.4, 24.1, 22.9, 19.5, 14.5; $[\alpha]_D^{20}$ = −91 (c = 1.0, MeOH); HRMS (ESI-TOF) m/z: calcd for $C_{28}H_{45}N_2O_3$ 457.3430 $[M+H]^+$, found 457.3424.

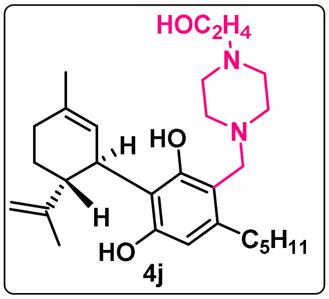

2'-[{4-(piperazin-1-yl)ethan-1-one}methyl]-cannabidiol (4k). ^{1}H NMR (400 MHz, $CDCl_3$) δ 6.16 (s, 1H), 5.98 (bs, 1H, OH), 5.60 (s, 1H), 4.36 (d, J = 34.4 Hz, 2H), 4.02 (s, 1H), 3.71 – 3.46 (m, 4H), 3.38 – 2.20 (m, 11H), 2.09 (s, 3H), 1.79 – 1.74 (m, 5H), 1.68 (s, 3H), 1.46 – 1.39 (m, 2H), 1.29 – 1.24 (m, 4H), 0.87 (t, J = 6.8 Hz, 3H); ^{13}C {^{1}H} NMR (101 MHz, $CDCl_3$) δ 169.4, 157.0, 155.7, 148.5, 140.6, 139.9, 125.0, 115.2, 111.0, 110.2, 109.2, 56.5, 52.4, 52.0, 47.2, 46.5, 41.7, 36.1, 33.8, 32.1, 31.3, 30.8, 28.4, 24.2, 22.9, 21.7, 19.5, 14.5; $[\alpha]_D^{20}$ = −68.6 (c = 1.0, MeOH); HRMS (ESI-TOF) m/z: calcd for $C_{28}H_{43}N_2O_3$ 455.3274 $[M+H]^+$, found 455.3272.

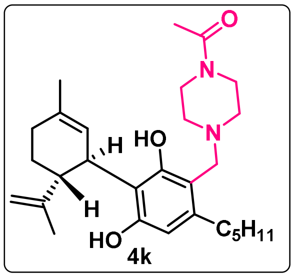

2'-{(4-phenylpiperazin-1-yl)methyl}-cannabidiol (4l). ^{1}H NMR (400 MHz, $CDCl_3$) δ 11.27 (bs, 1H, OH), 7.31 – 7.26 (m, 2H), 6.95 – 6.87 (m, 3H), 6.19 (s, 1H), 5.98 (s, 1H), 5.63 (s, 1H), 4.38 (d, J = 36 Hz, 2H), 4.03 (s, 1H), 3.68 (dd, J = 58.8, 13.2 Hz, 2H), 3.23 – 2.49 (m, 8H), 2.47 – 2.31 (m, 3H), 2.27 – 2.06 (m, 2H), 1.81 – 1.75 (m, 5H), 1.70 (s, 3H), 1.52 – 1.44 (m, 2H), 1.36 – 1.28 (m, 4H), 0.90 (t, J = 6.8 Hz, 3H); ^{13}C {^{1}H} NMR (101 MHz, $CDCl_3$) δ 157.3, 155.5, 151.4, 148.5, 140.6, 139.8, 129.6, 125.1, 120.6, 116.7, 115.2, 111.0, 110.6, 109.0, 56.6, 52.5, 49.6, 47.3, 36.1, 33.9, 32.1, 31.3, 30.8, 28.4, 24.2, 23.0, 19.5, 14.5; $[\alpha]_D^{20}$ = −87 (c = 1.0, MeOH); HRMS (ESI-TOF) m/z: calcd for $C_{32}H_{45}N_2O_2$ 489.3481 $[M+H]^+$, found 489.3481.

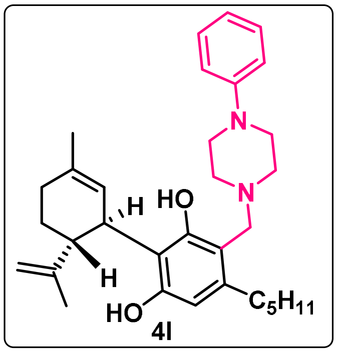

2'-{(4-cyclopropylpiperazin-1-yl)methyl}-cannabidiol (4m). ^{1}H NMR (400 MHz, $CDCl_3$) δ 6.14 (s, 1H), 5.94 (bs, 1H, OH), 5.61 (s, 1H), 4.37 (d, J = 38.8 Hz, 2H), 4.03 (d, J = 9.6 Hz, 1H), 3.59 (dd, J = 57.6, 13.6 Hz, 2H), 2.73 – 2.56 (m, 6H), 2.42 – 2.37 (m, 3H), 2.22 – 2.05 (m, 2H), 1.79 – 1.75 (m, 5H), 1.72 (s, 3H), 1.64 – 1.60 (m, 1H), 1.45 – 1.39 (m, 2H), 1.32 – 1.24 (m, 6H), 0.86 (t, J = 6.8 Hz, 3H), 0.46 – 0.39 (m, 4H); ^{13}C NMR (101 MHz, $CDCl_3$) δ 157.4, 155.4, 148.4, 140.5, 139.7, 125.2, 115.1, 111.0, 110.8, 108.8, 56.6, 53.5, 47.3, 38.7, 36.1, 33.8, 32.2, 31.3, 30.8, 30.1, 28.4, 24.2, 23.0, 19.6, 14.5, 6.3; $[\alpha]_D^{20}$ = −101.6 (c = 1.0, MeOH); HRMS (ESI-TOF) m/z: calcd for $C_{29}H_{45}N_2O_2$ 453.3481 $[M+H]^+$, found 453.3483.

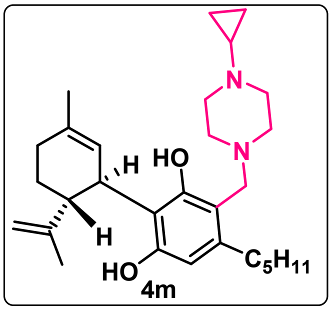

2'-[{4-(4-chlorophenyl)piperazine}methyl]-cannabidiol (4n). ^{1}H NMR (400 MHz, $CDCl_3$) δ 11.56 (bs, 1H, OH), 7.65 – 7.60 (m, 2H), 7.25 – 7.22 (m, 2H), 6.58 (s, 1H), 6.37 (bs, 1H, OH), 6.01 (s, 1H), 4.77 (d, J = 33.2 Hz, 2H), 4.43 (s, 1H), 4.07 (dd, J = 56.8, 13.6 Hz, 2H), 3.59 – 2.91 (m, 8H), 2.87 – 2.78 (m, 3H), 2.64 – 2.44 (m, 2H), 2.20 – 2.15 (m, 5H), 2.09 (s, 3H), 1.90 – 1.83 (m, 2H), 1.73 – 1.68 (m, 4H), 1.28 (t, J = 6.8 Hz, 3H); ^{13}C {^{1}H} NMR (101 MHz, $CDCl_3$) δ 157.2, 155.5, 149.9, 148.4, 140.6, 139.8, 129.4, 125.4, 125.0, 117.9, 115.2, 111.0, 110.4, 109.0, 56.5, 52.2, 49.6, 47.2, 36.0, 33.8, 32.1, 31.3, 30.8, 28.4, 24.1, 23.0, 19.5, 14.5; $[\alpha]_D^{20}$ = −98 (c = 1.0, MeOH); HRMS (ESI-TOF) m/z: calcd for $C_{32}H_{44}ClN_2O_2$ 523.3091 $[M+H]^+$, found 523.3090.

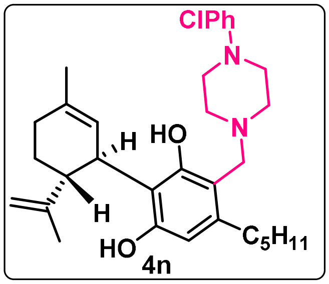

2'-[{4-(pyrimidin-2-yl)piperazin-1-yl}methyl]-cannabidiol (4o). ^{1}H NMR (400 MHz, $CDCl_3$) δ 11.31 (bs, 1H, OH), 8.31(d, J =4.8 Hz, 2H), 6.51 (t, J = 4.8 Hz 1H), 6.17 (s, 1H), 5.98 (bs, 1H, OH), 5.63 (s, 1H), 4.39 (d, J =48 Hz, 2H), 4.04 (d, J = 9.6 Hz, 1H), 3.65 (dd, J = 13.6, 68 Hz, 2H), 2.58 – 2.52 (m, 2H), 2.48 – 2.23 (m, 5H), 2.21 – 2.04 (m, 2H), 1.80 – 1.76 (m, 5H), 1.71 (s, 3H), 1.63 – 1.42 (m, 4H), 1.31 – 1.26 (m, 4H), 1.03 (t, J =7.6 Hz, 2H), 0.86 (t, J = 6.8 Hz, 3H); ^{13}C {^{1}H} NMR (101 MHz, $CDCl_3$) δ 161.0, 158.2, 157.3, 155.6, 148.4, 140.6, 139.8, 125.1, 115.2, 111.1, 110.6, 110.5, 109.0, 56.8, 52.3, 47.3, 46.5, 36.1, 33.9, 32.1, 31.2, 30.8, 28.4, 24.2, 23.0, 19.5, 14.5; $[\alpha]_D^{20}$ = −83 (c = 1.0, MeOH); HRMS (ESI-TOF) m/z: calcd for $C_{30}H_{43}N_4O_2$ 491.3386 $[M+H]^+$, found 491.3382.

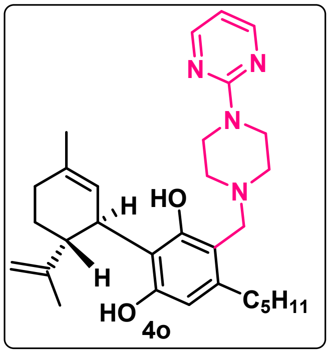

NMR and HRMS spectrum

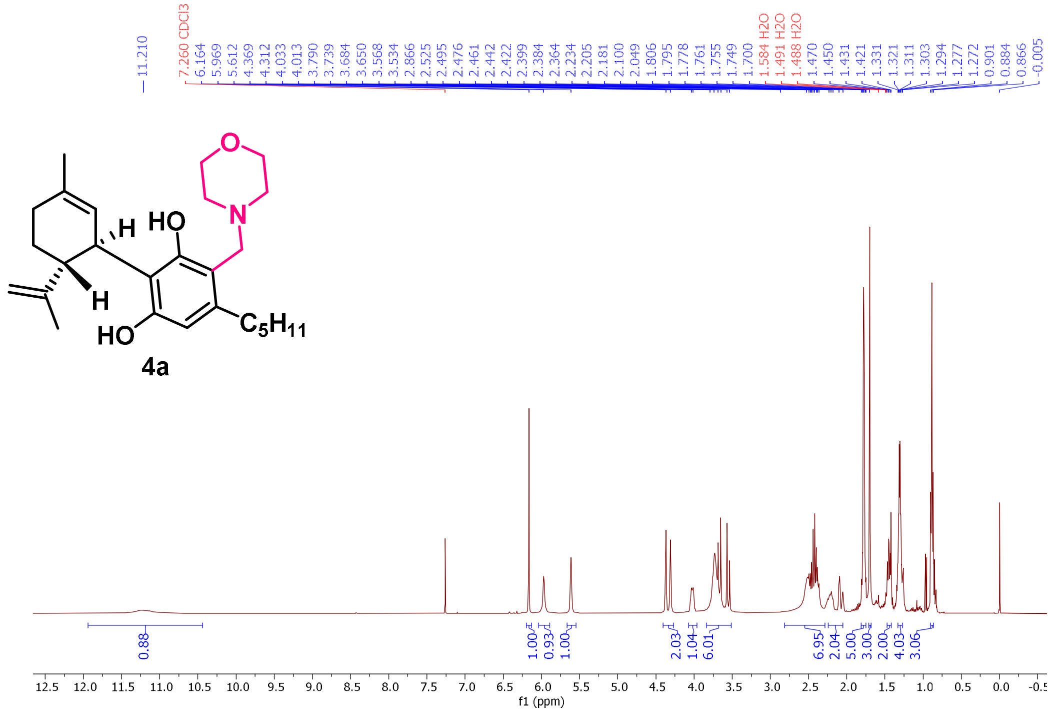

Figure S5. ^{1}H NMR spectrum of **4a**

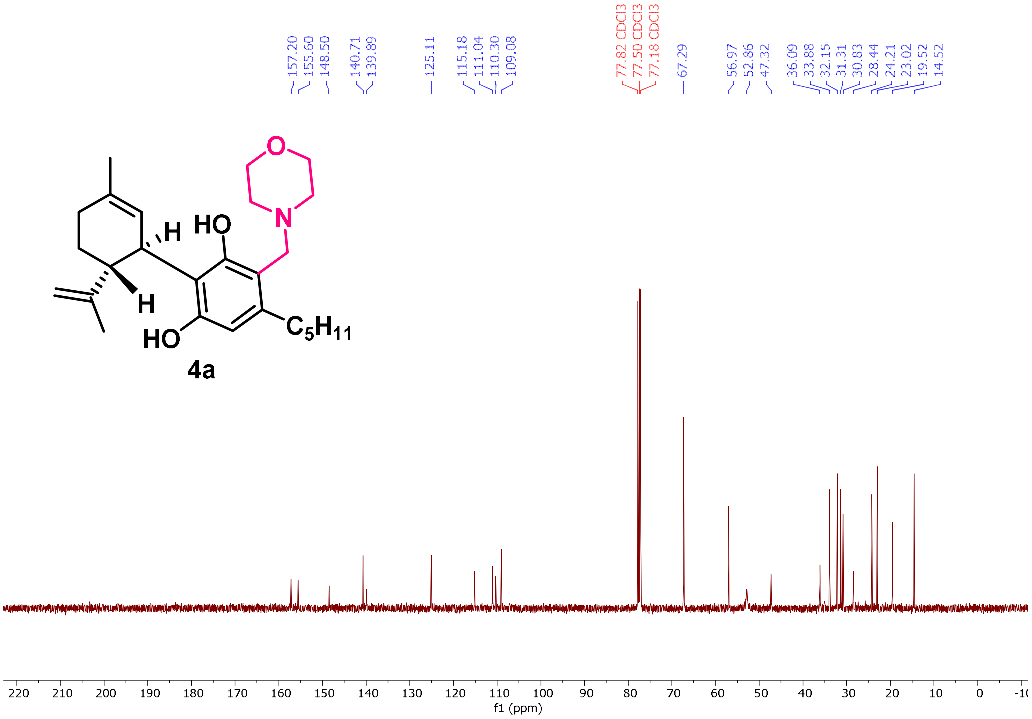

Figure S6. $^{13}C\{^{1}H\}$ NMR spectrum of **4a**

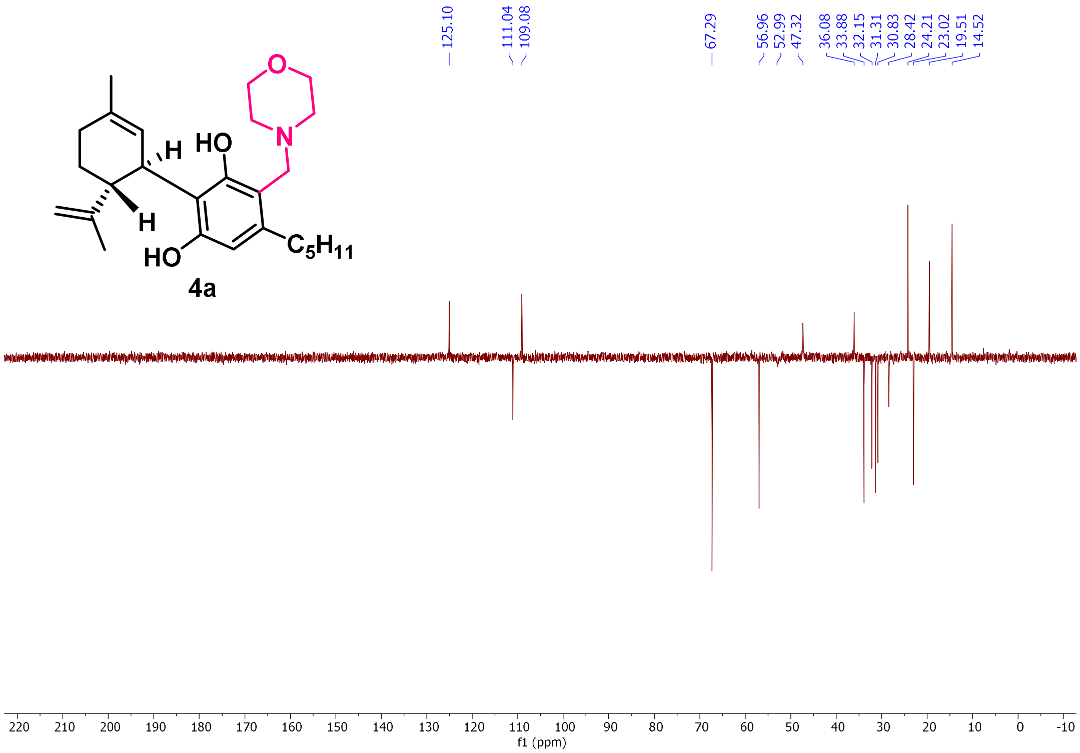

Figure S7. DEPT spectrum of **4a**

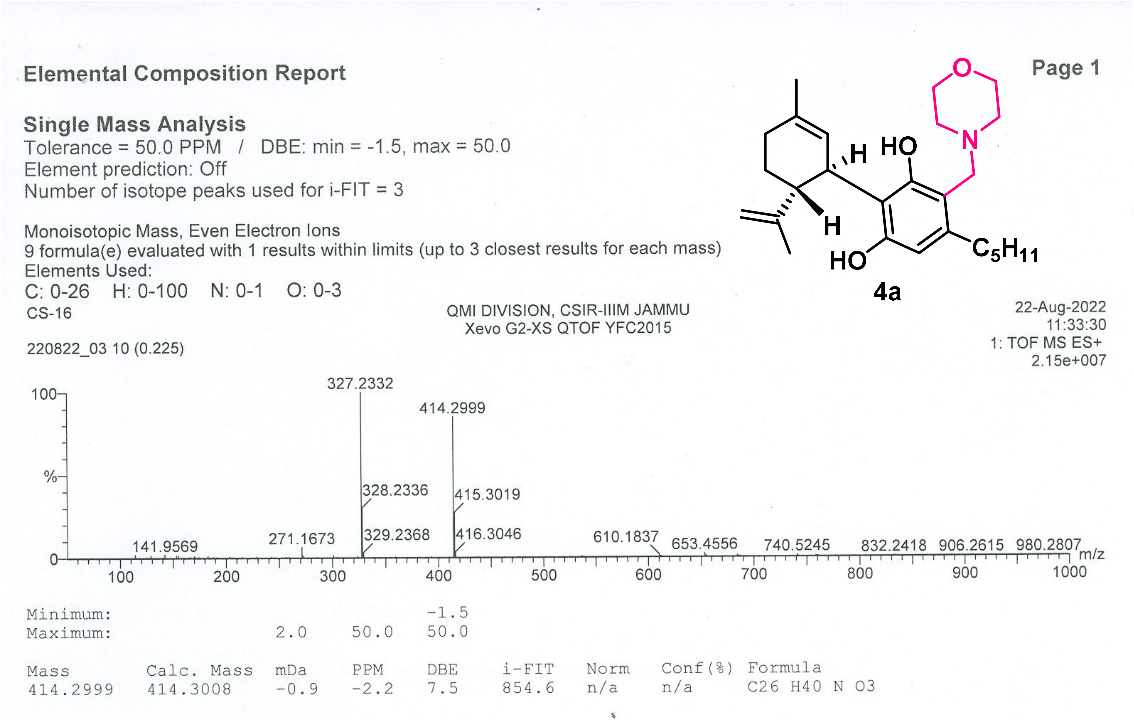

Figure S8. HRMS spectrum of **4a**

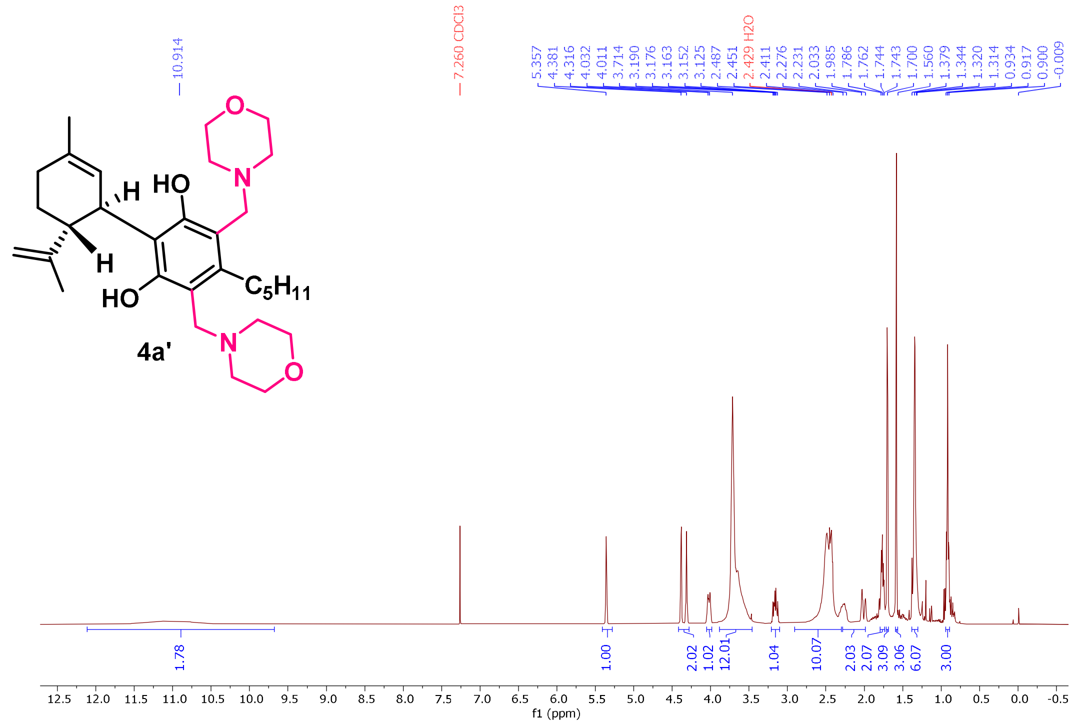

Figure S9. ^{1}H NMR spectrum of **4a'**

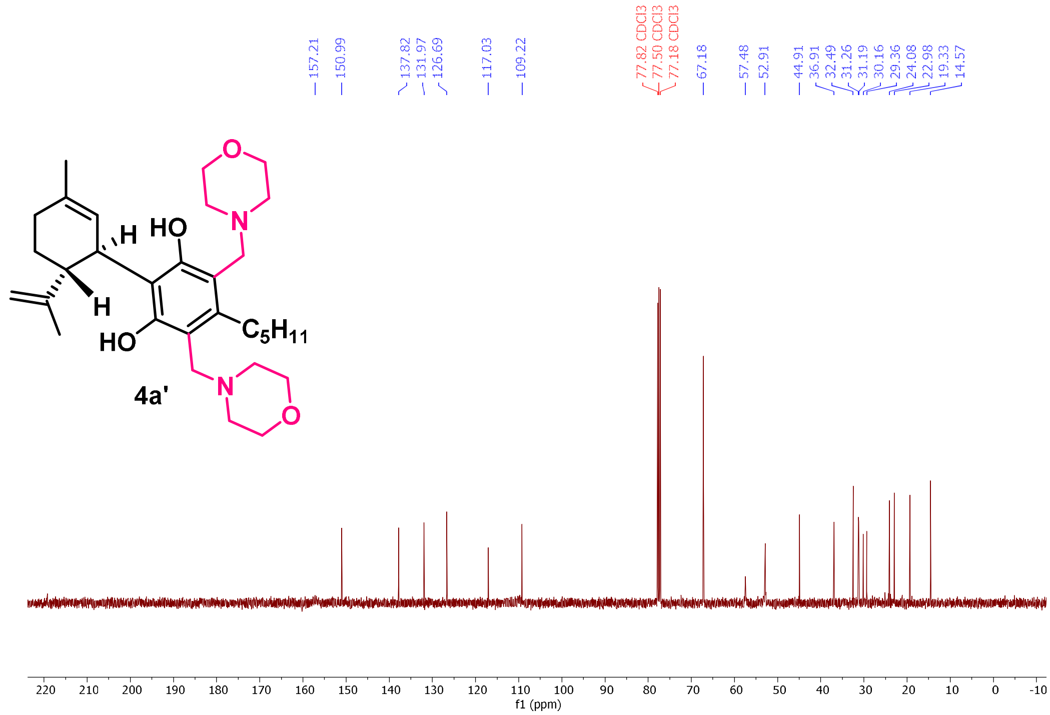

Figure S10. $^{13}C\{^{1}H\}$ NMR spectrum of **4a'**

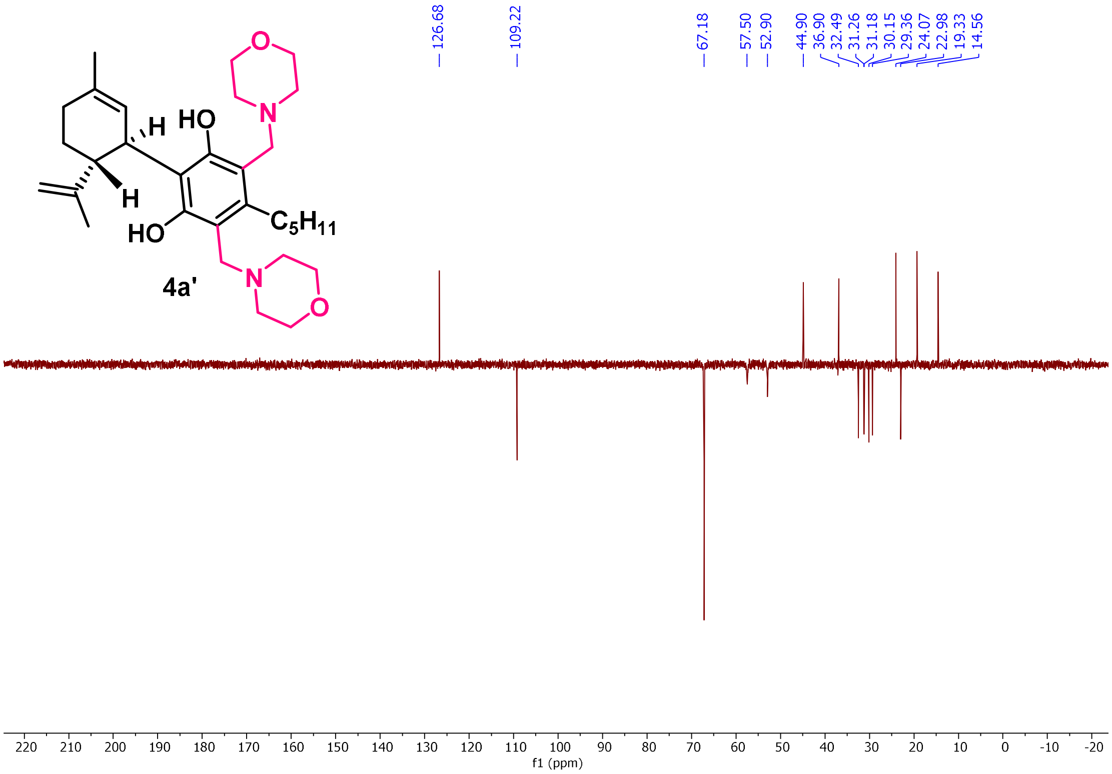

Figure S11. DEPT spectrum of **4a'**

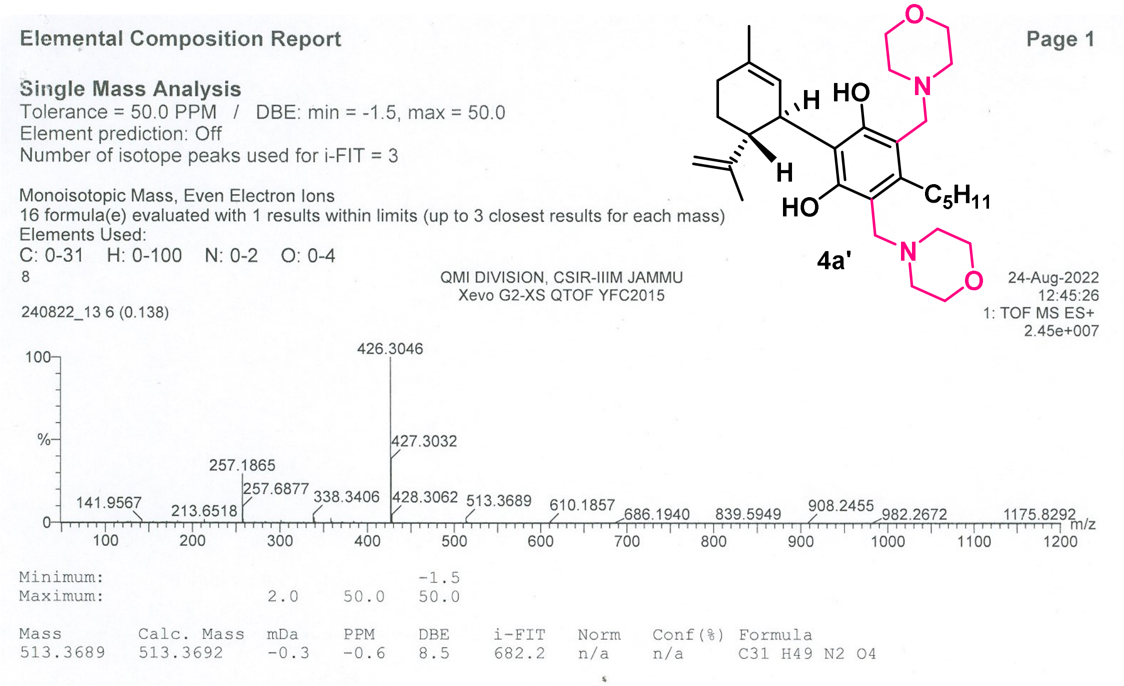

Figure S12. HRMS spectrum of **4a'**

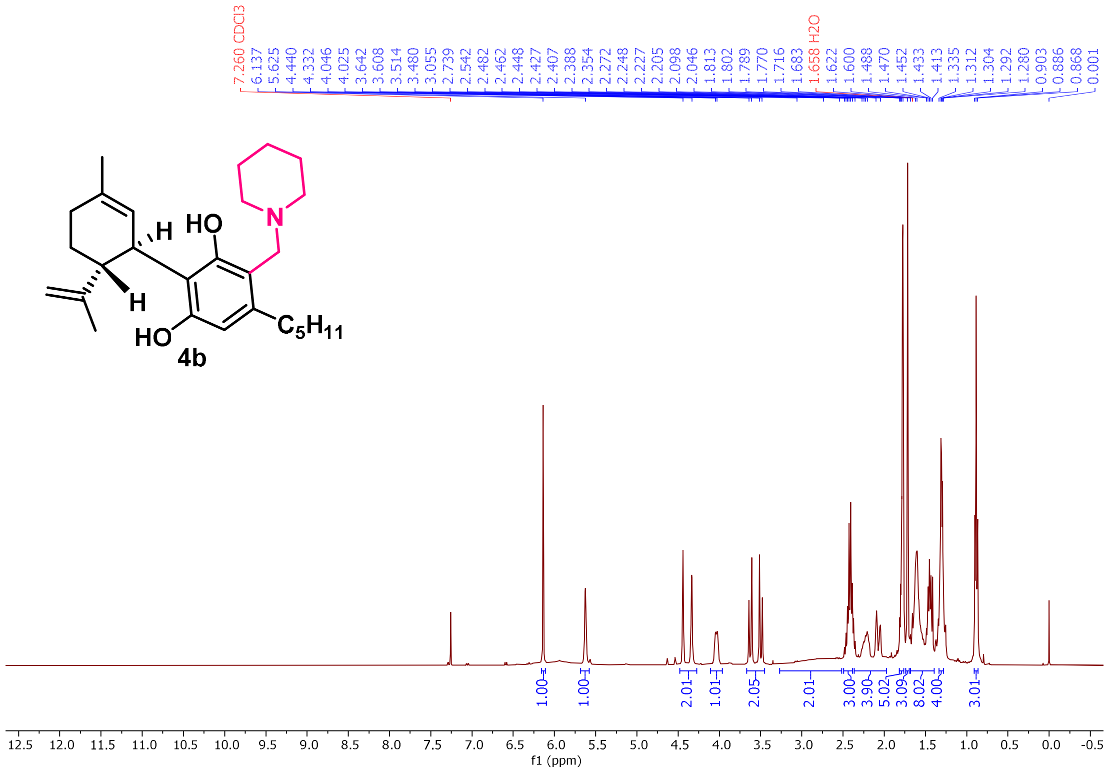

Figure S13. ^{1}H NMR spectrum of **4b**

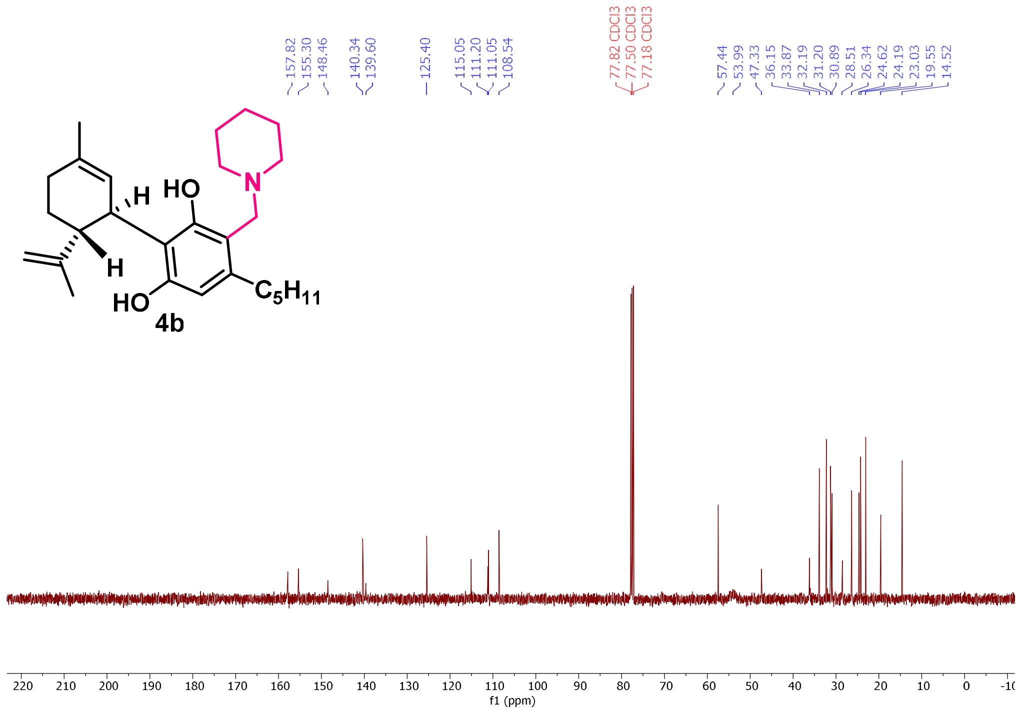

Figure S14. $^{13}C\{^{1}H\}$ NMR spectrum of **4b**

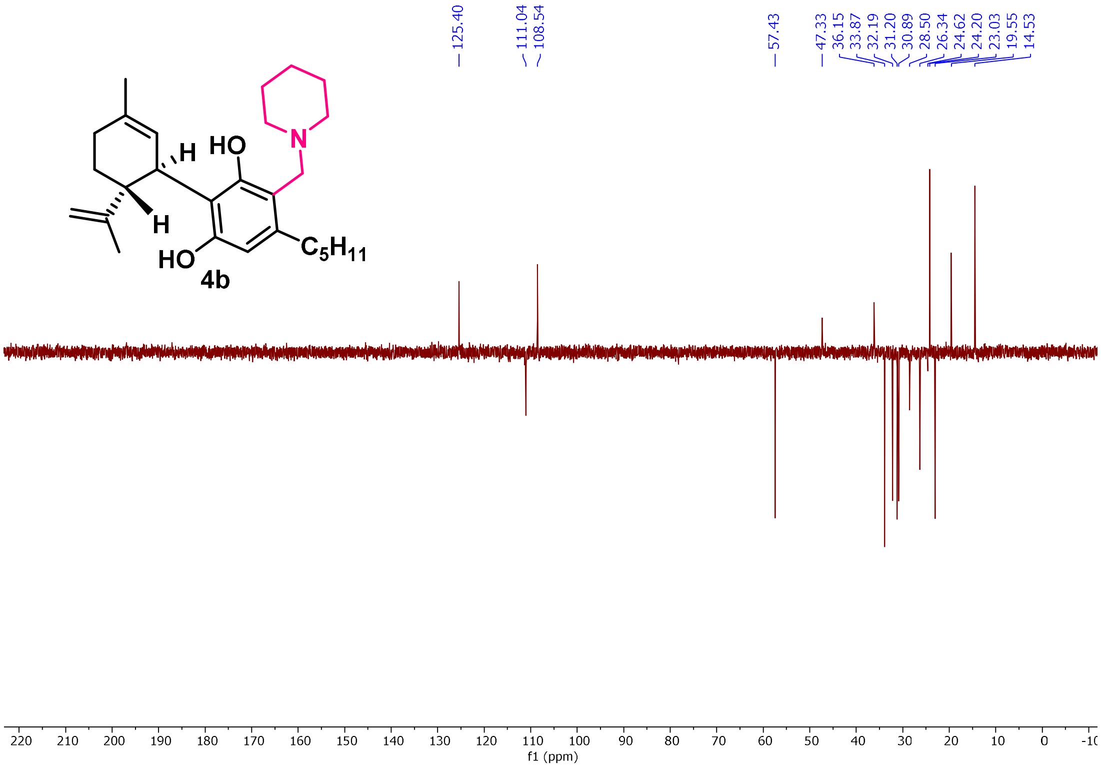

Figure S15. DEPT spectrum of **4b**

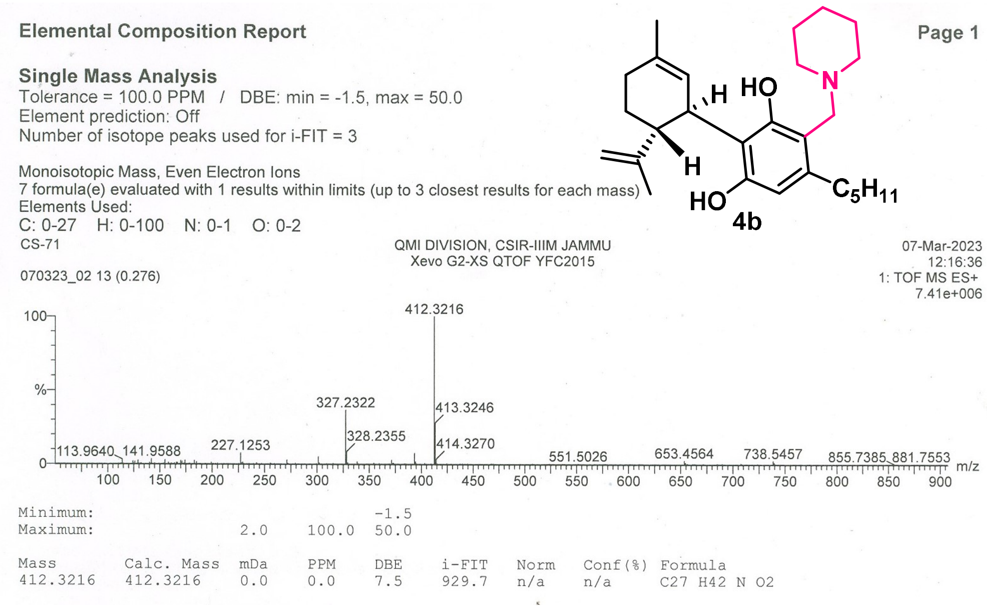

Figure S16. HRMS spectrum of **4b**

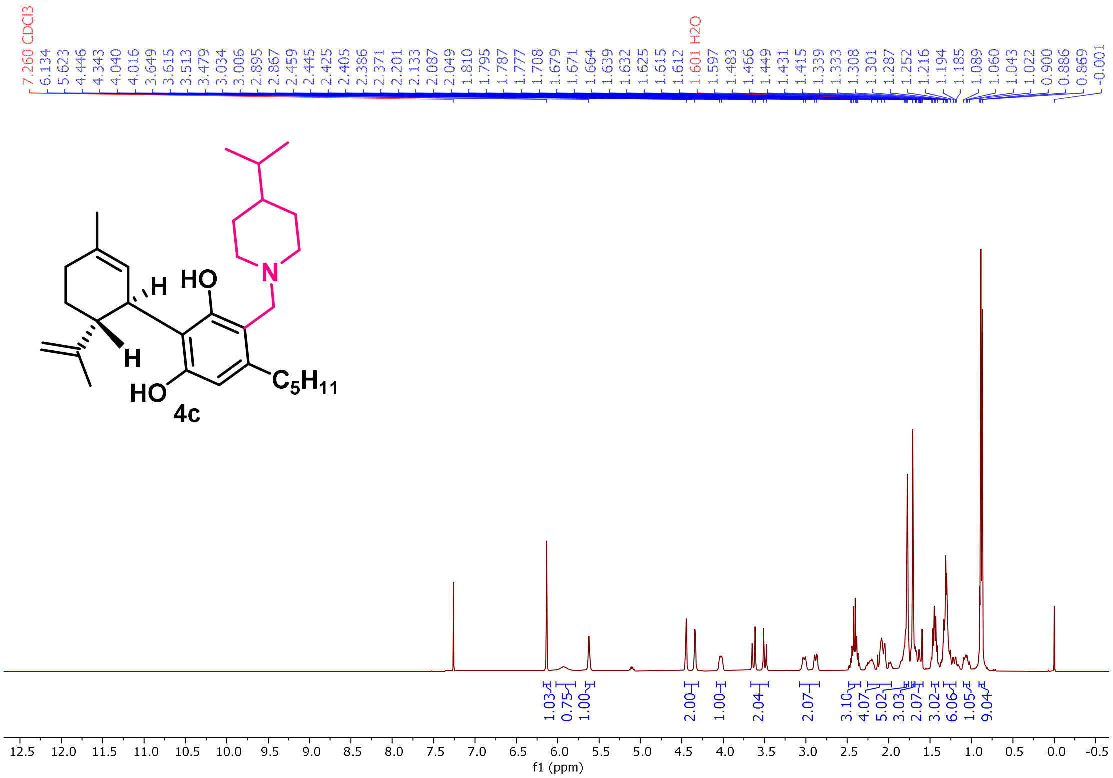

Figure S17. ^{1}H NMR spectrum of **4c**

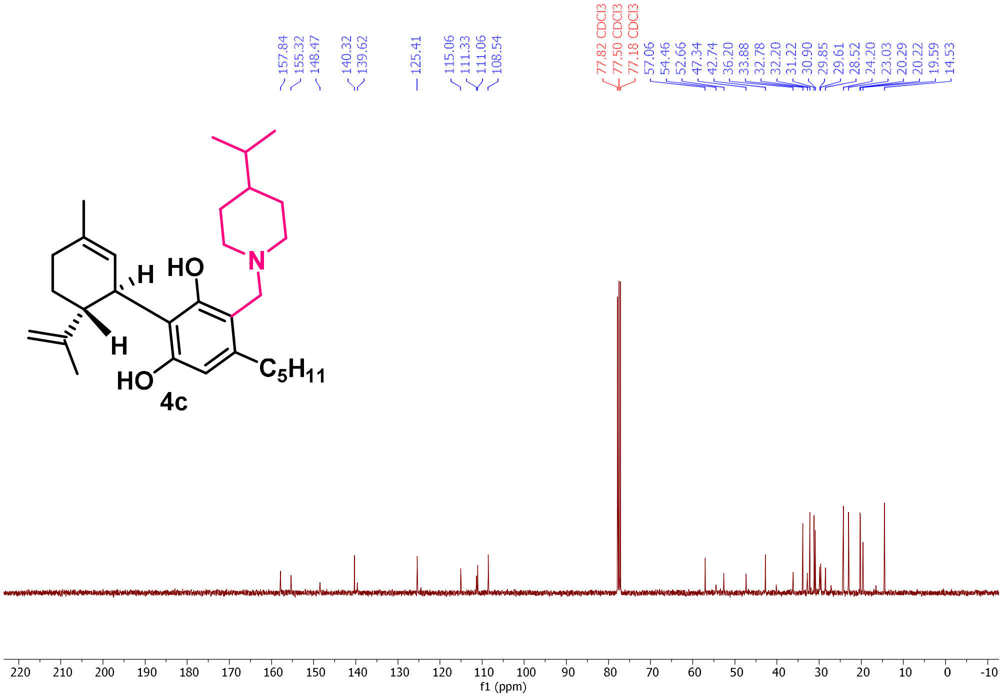

Figure S18. ^{13}C{^{1}H} NMR spectrum **of 4c**

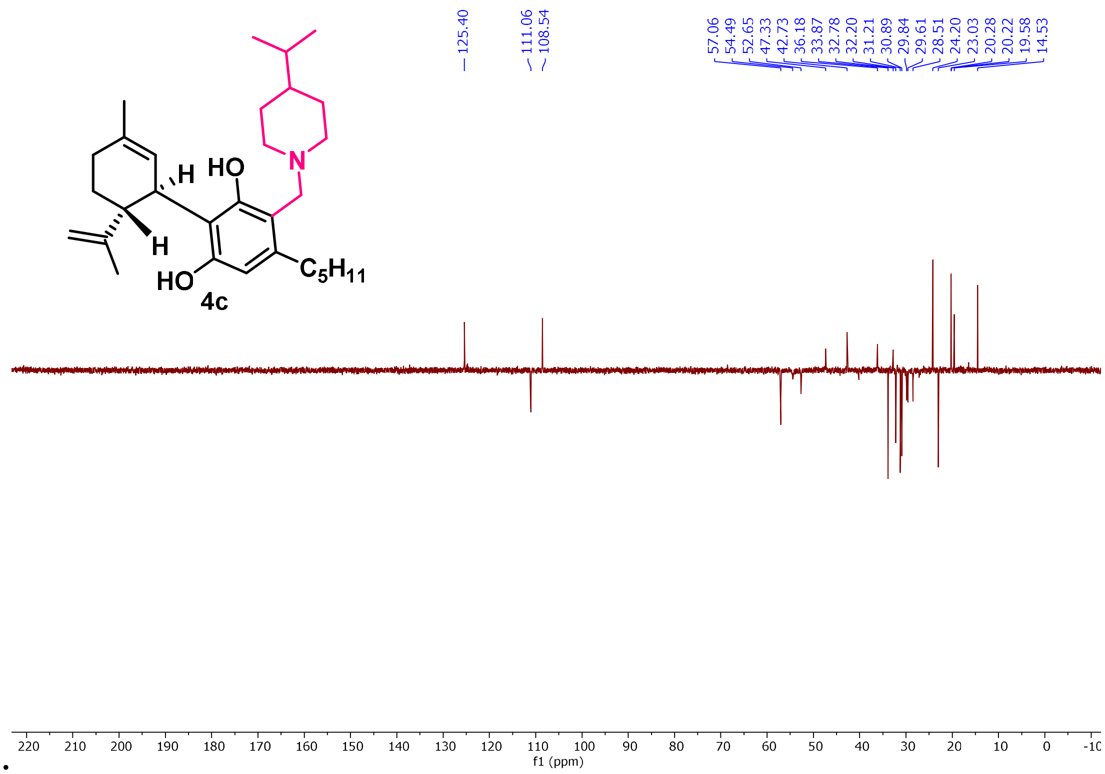

Figure S19. DEPT spectrum of **4c**

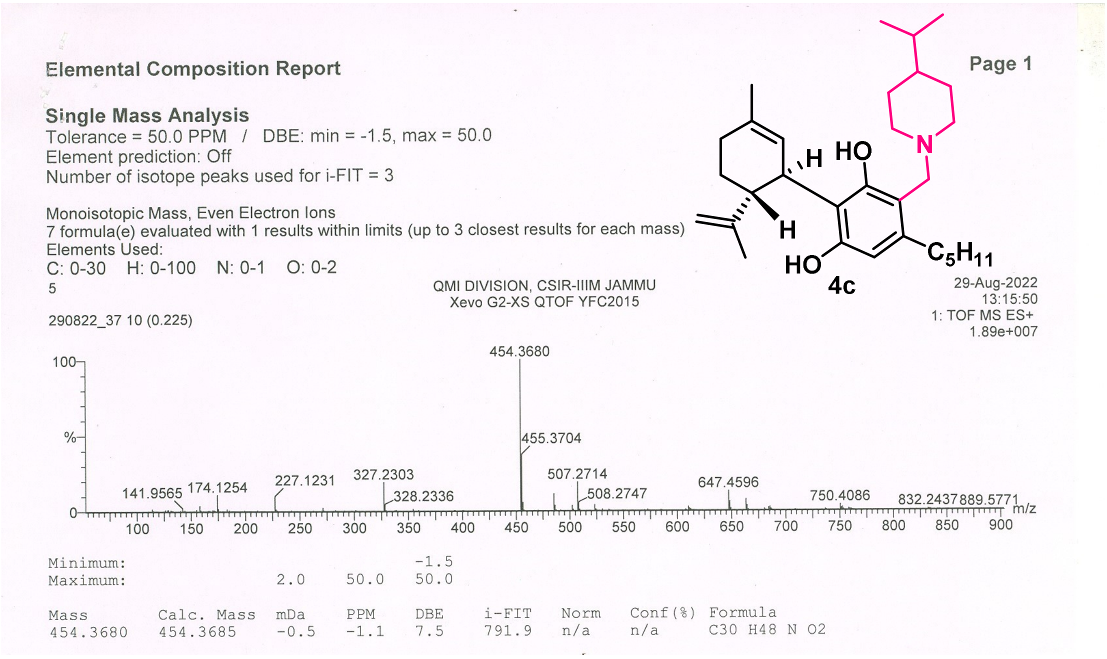

Figure S20. HRMS spectrum of **4c**

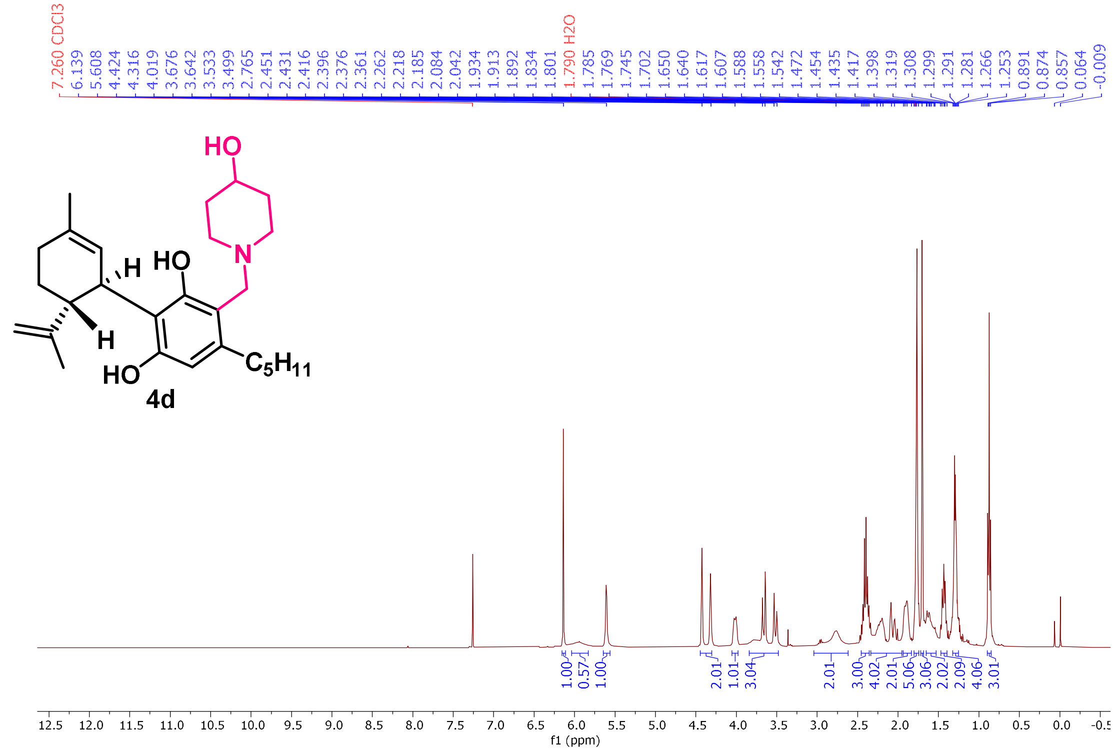

Figure S21. ^{1}H NMR spectrum of **4d**

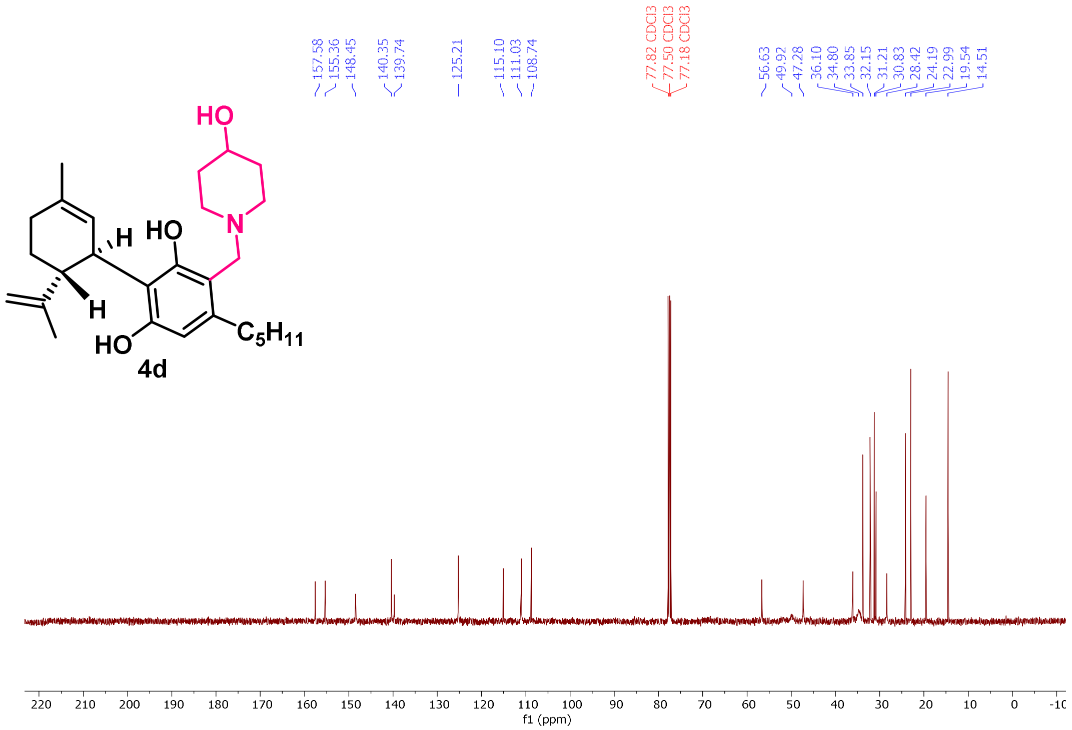

Figure S22. $^{13}C\{^1H\}$ NMR spectrum **of 4d**

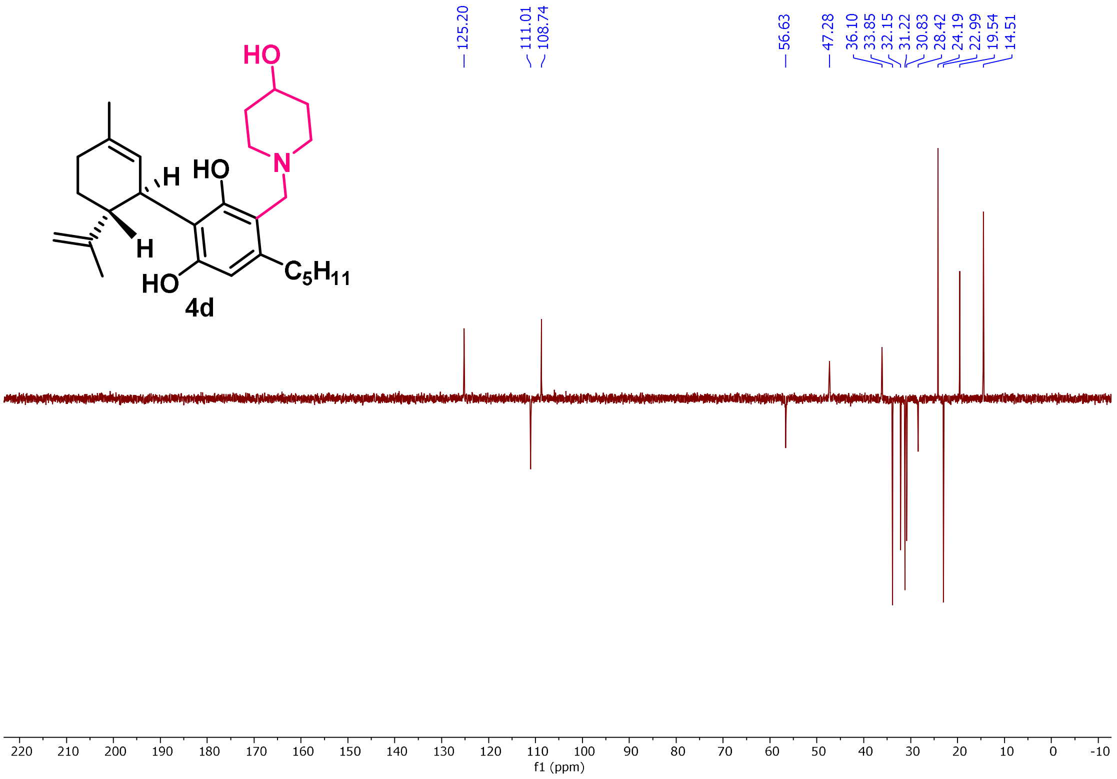

Figure S23. DEPT spectrum of **4d**

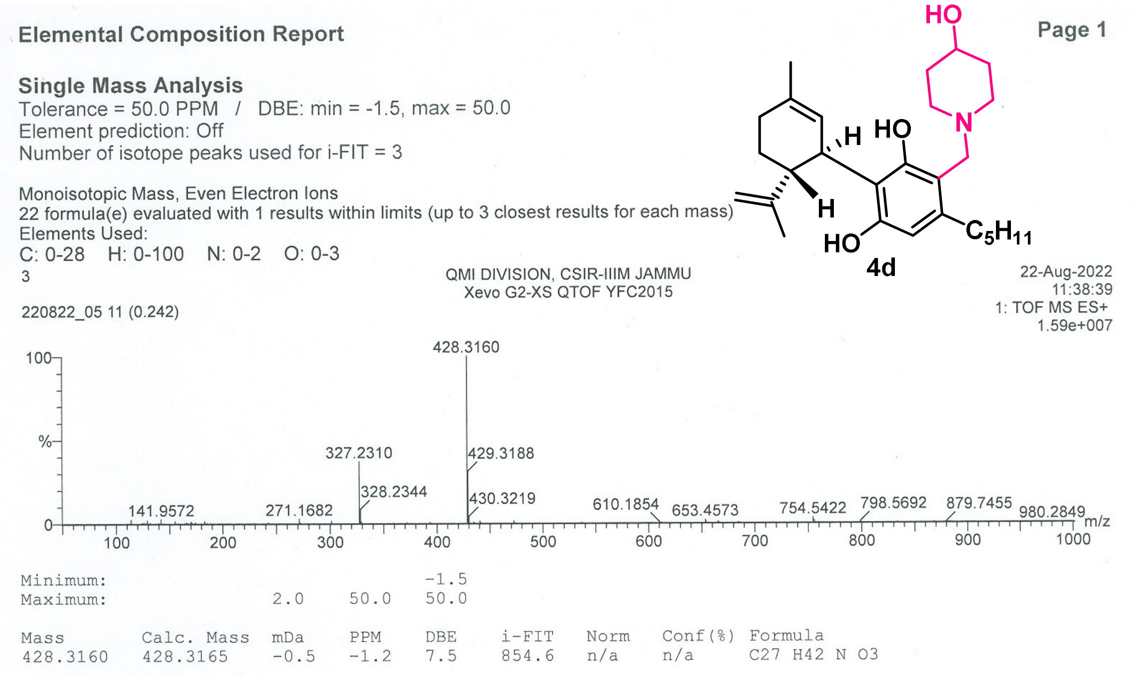

Figure S24. HRMS spectrum of **4d**

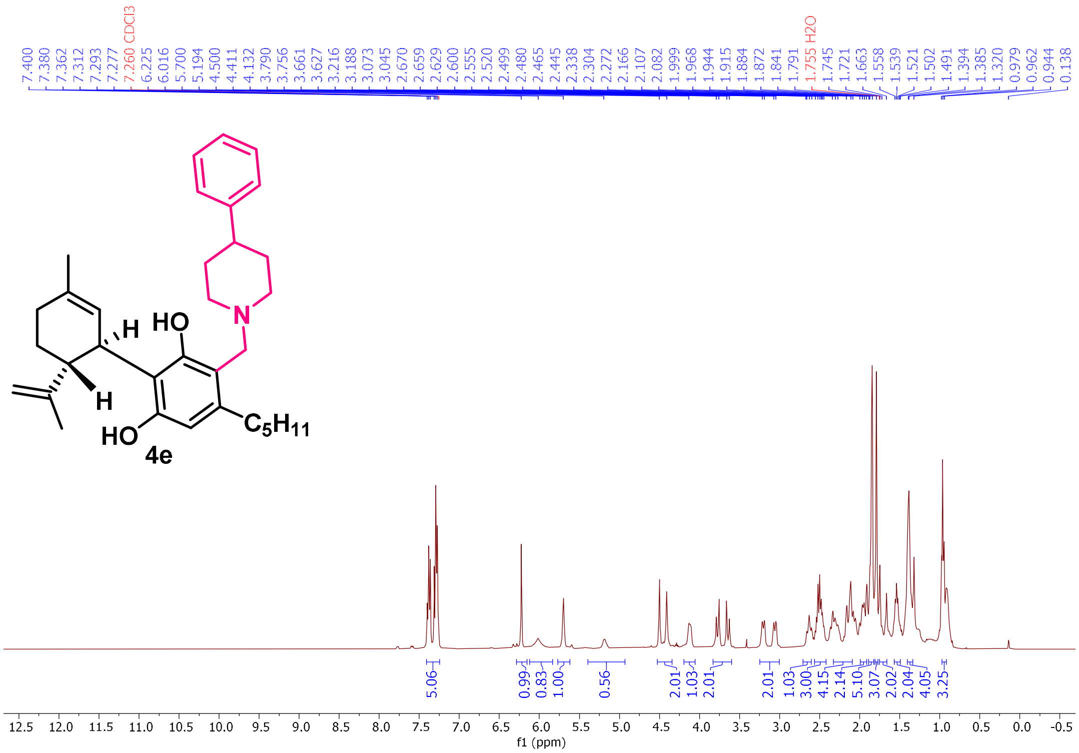

Figure S25. ^{1}H NMR spectrum of **4e**

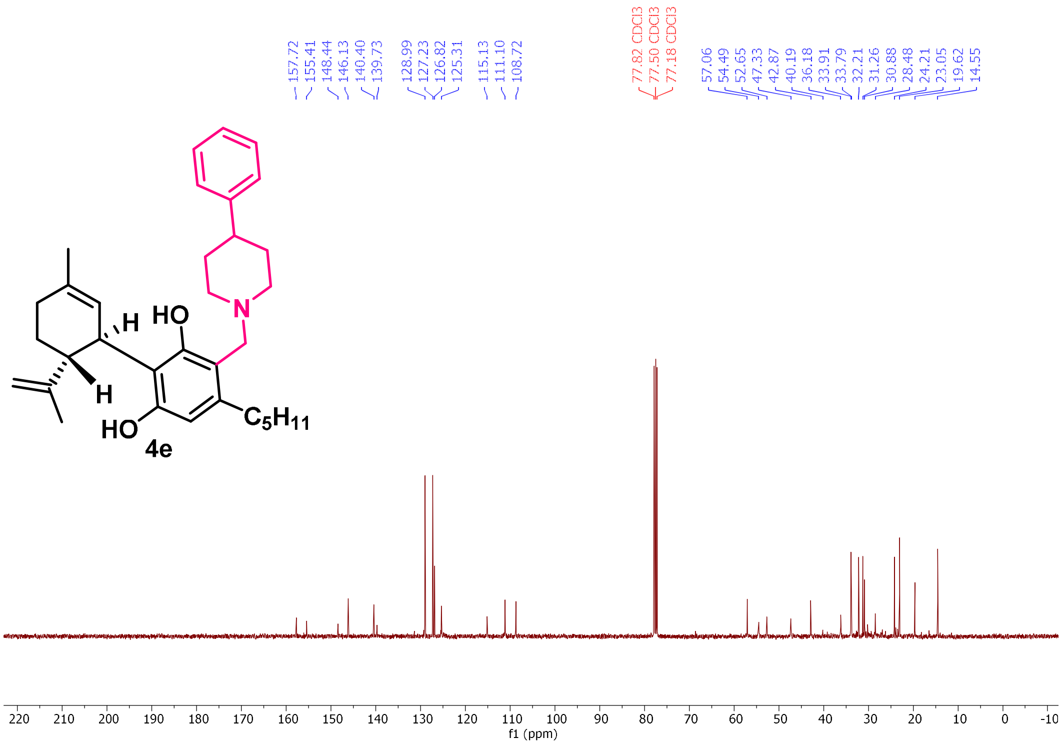

Figure S26. $^{13}C\{^{1}H\}$ NMR spectrum of **4e**

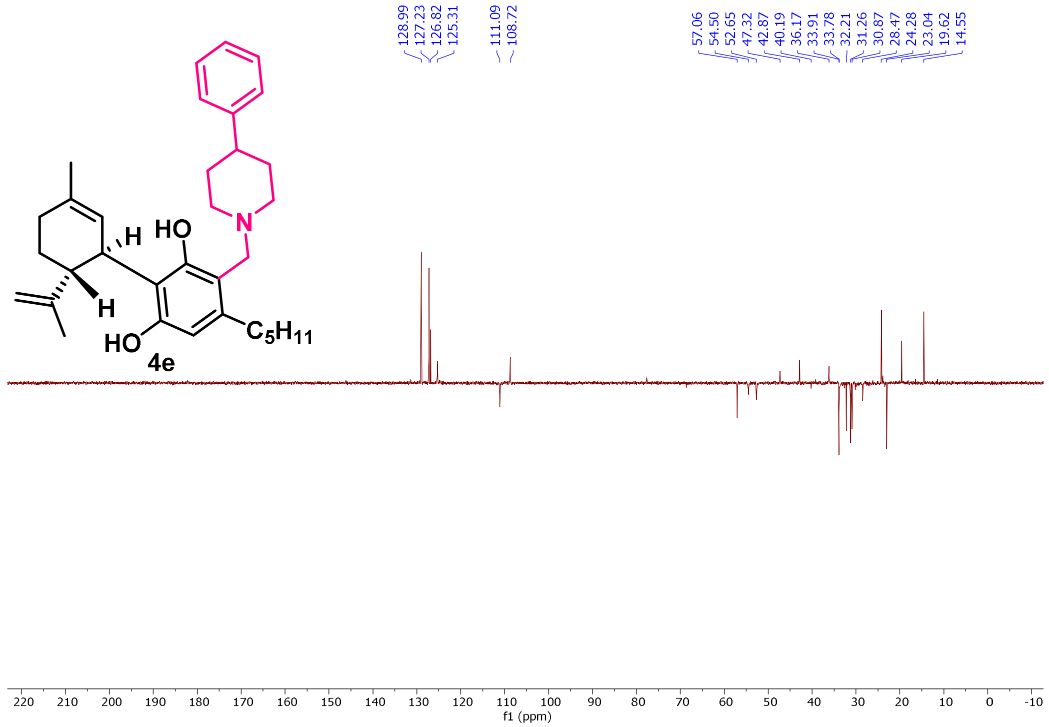

Figure S2. DEPT spectrum of **4e**

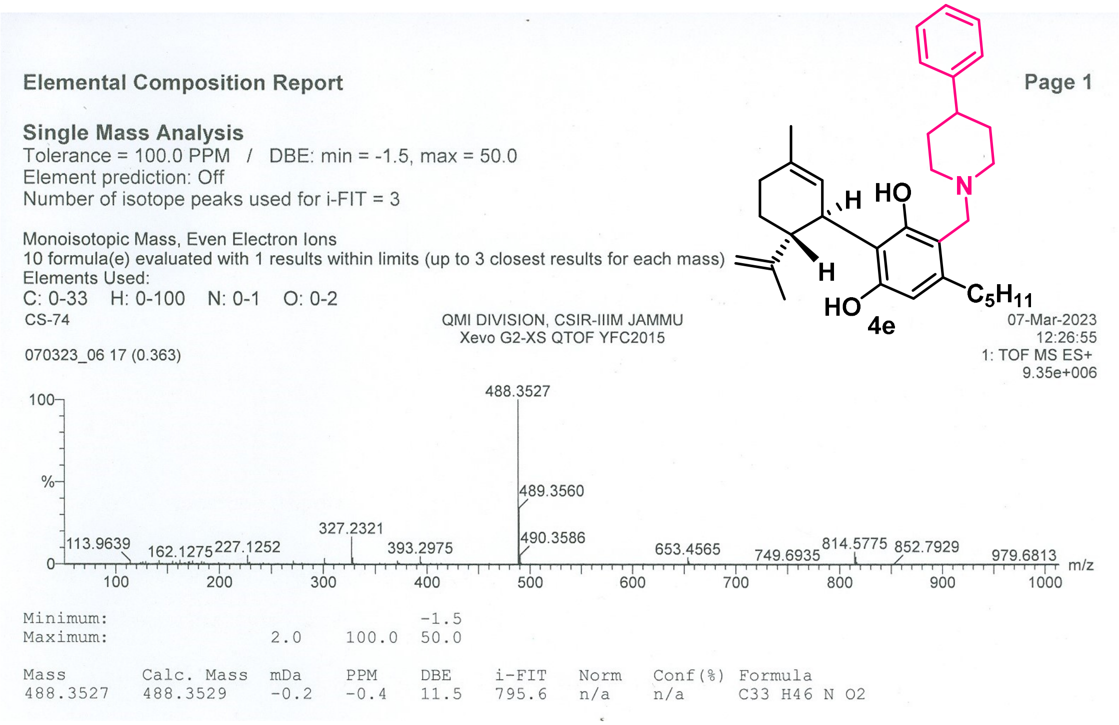

Figure S28. HRMS spectrum of **4e**

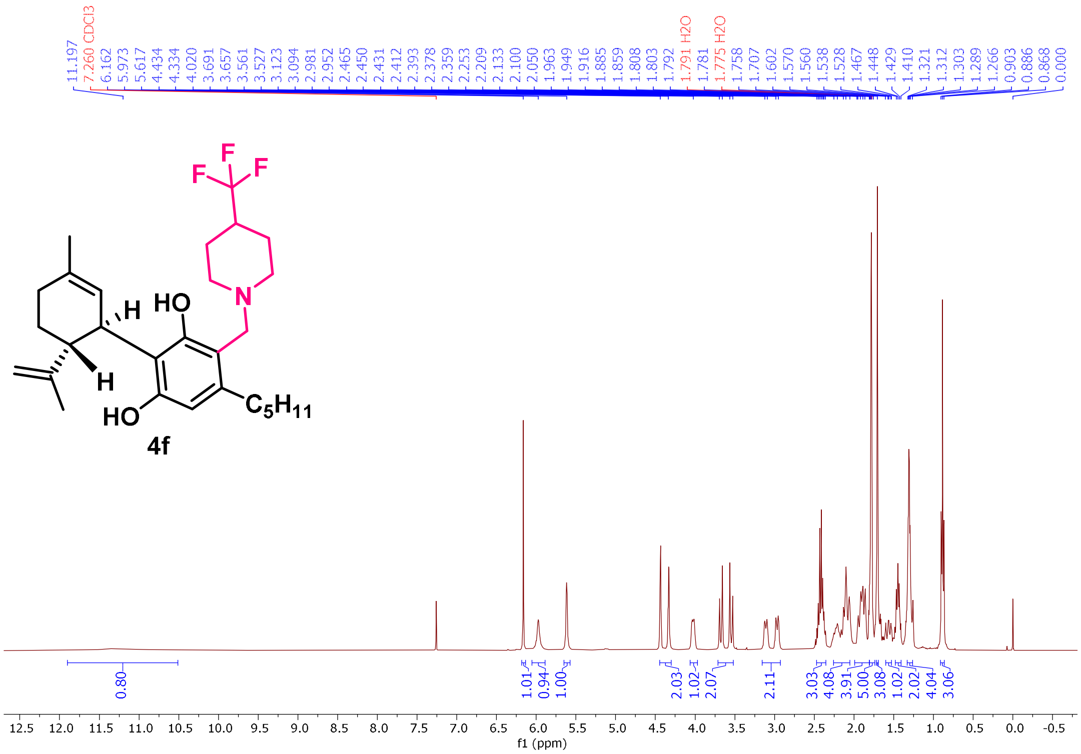

Figure S29. ^{1}H NMR spectrum of **4f**

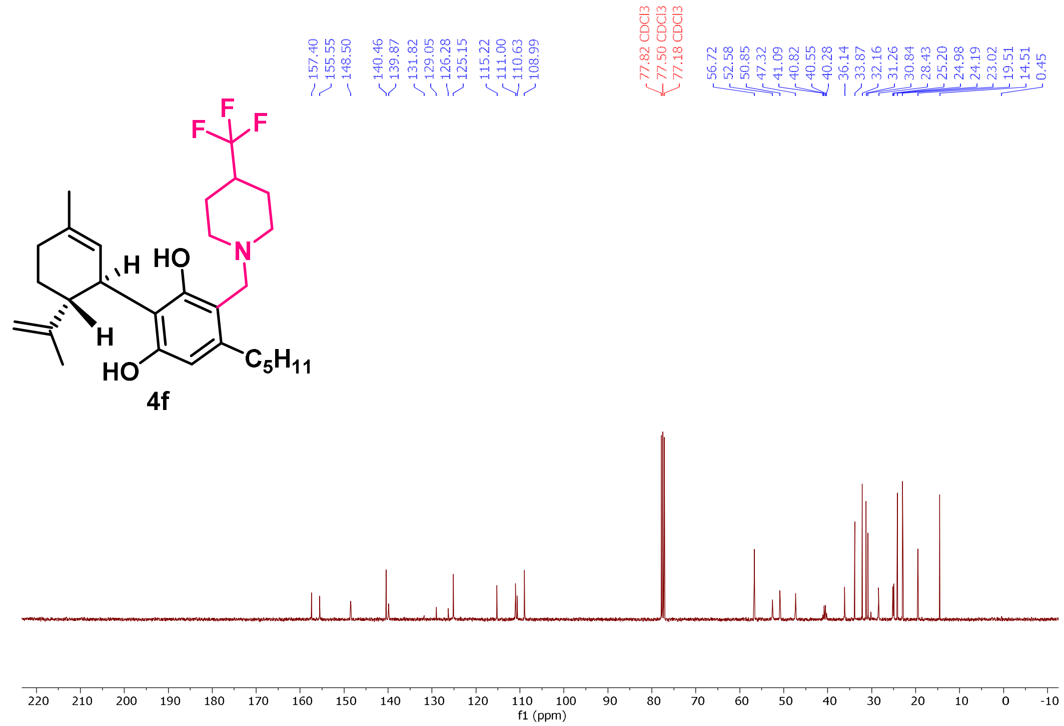

Figure S30. ^{13}C{^{1}H} NMR spectrum of **4f**

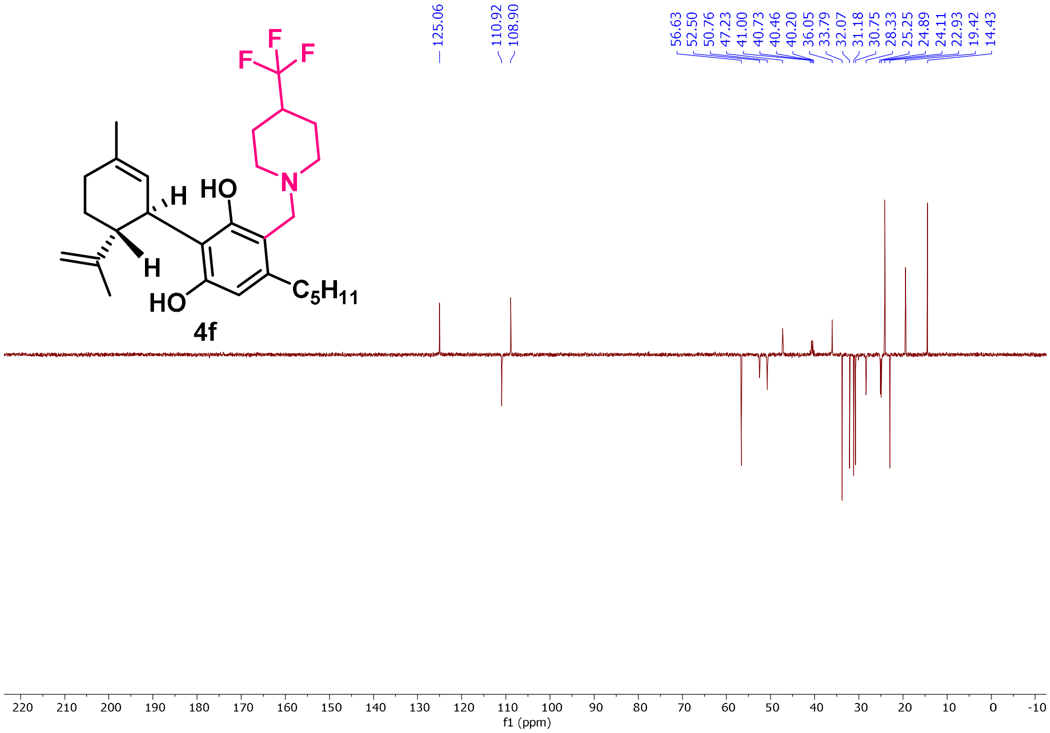

Figure S31. DEPT spectrum of **4f**

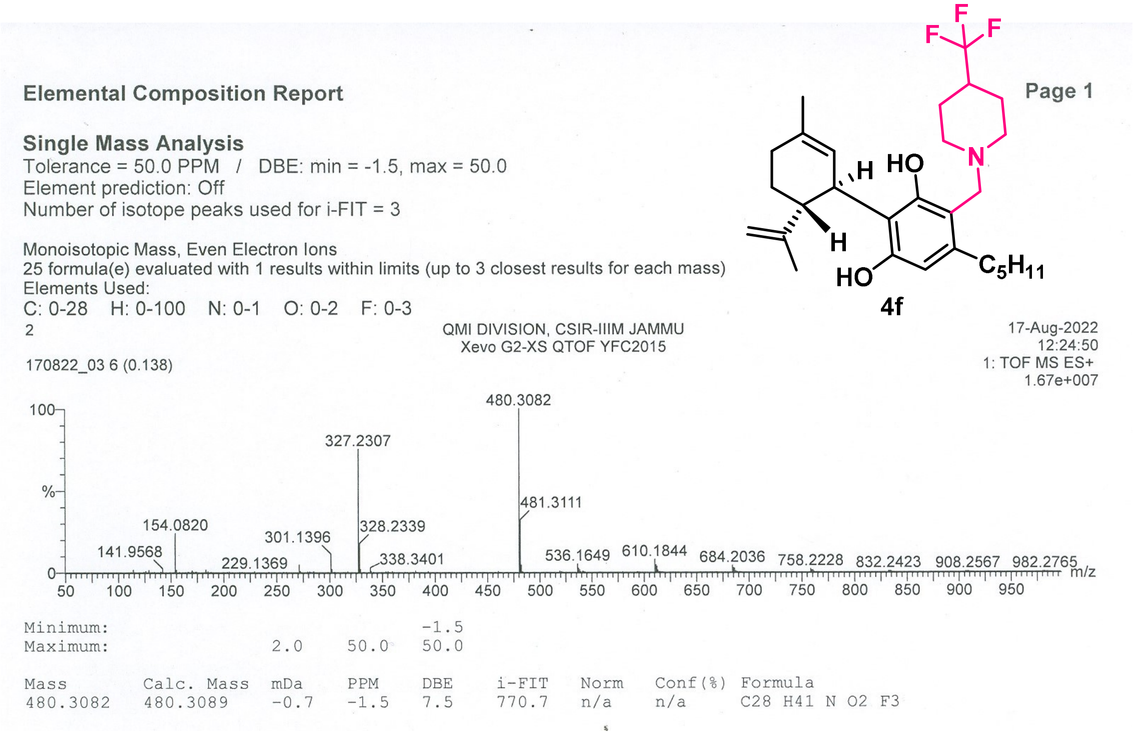

Elemental Composition Report | **Page 1**

Single Mass Analysis
Tolerance = 50.0 PPM / DBE: min = -1.5, max = 50.0
Element prediction: Off
Number of isotope peaks used for i-FIT = 3

Monoisotopic Mass, Even Electron Ions
25 formula(e) evaluated with 1 results within limits (up to 3 closest results for each mass)
Elements Used:
C: 0-28 H: 0-100 N: 0-1 O: 0-2 F: 0-3
2

QMI DIVISION, CSIR-IIIM JAMMU
Xevo G2-XS QTOF YFC2015

17-Aug-2022
12:24:50
1: TOF MS ES+
1.67e+007

170822_03 6 (0.138)

Minimum:				-1.5				
Maximum:		2.0	50.0	50.0				
Mass	Calc. Mass	mDa	PPM	DBE	i-FIT	Norm	Conf(%)	Formula
480.3082	480.3089	-0.7	-1.5	7.5	770.7	n/a	n/a	C28 H41 N O2 F3

Figure S32. HRMS spectrum of **4f**

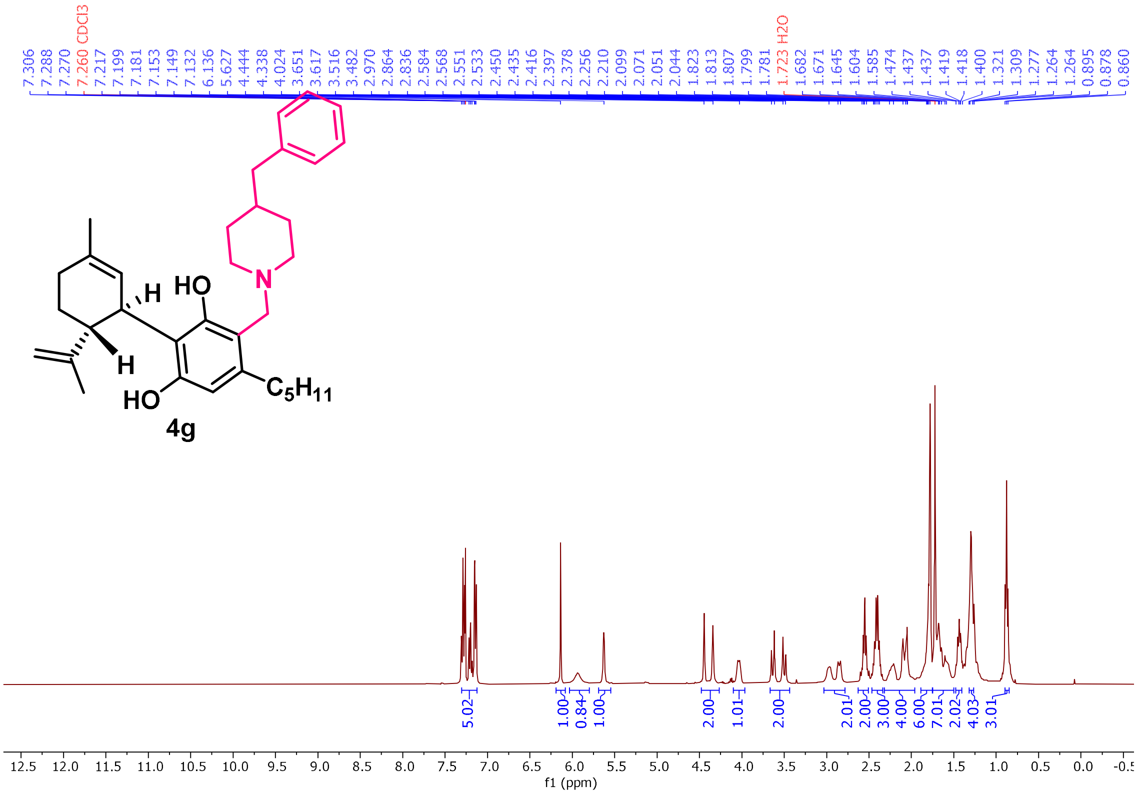

Figure S33. ^{1}H NMR spectrum of **4g**

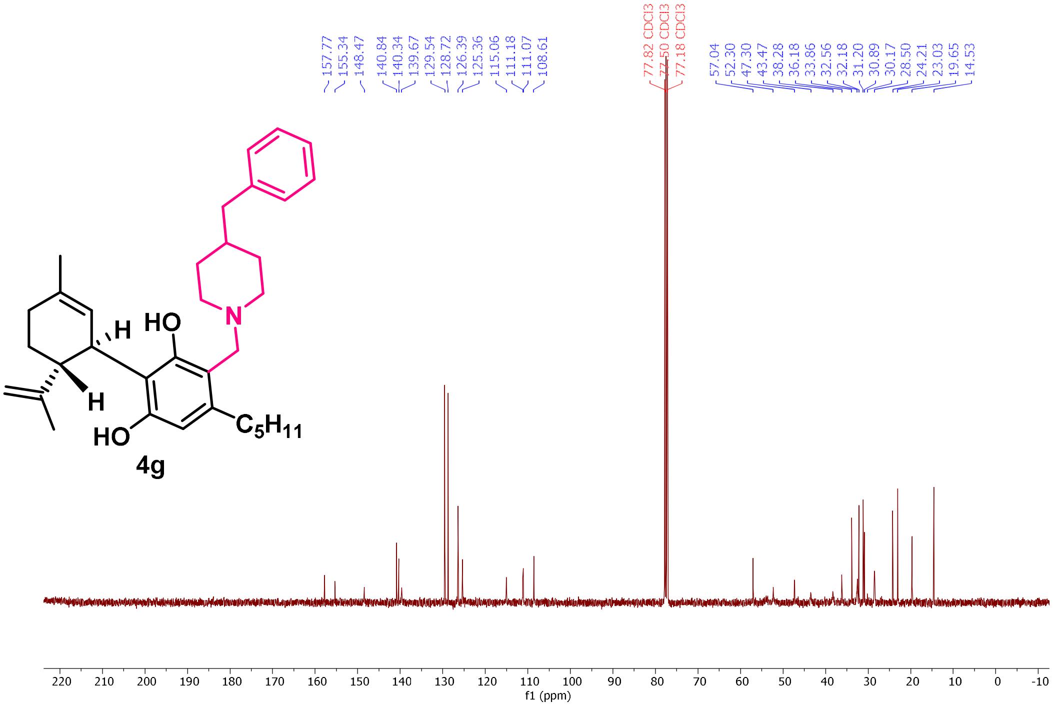

Figure S34. ^{13}C{^{1}H} NMR spectrum of **4g**

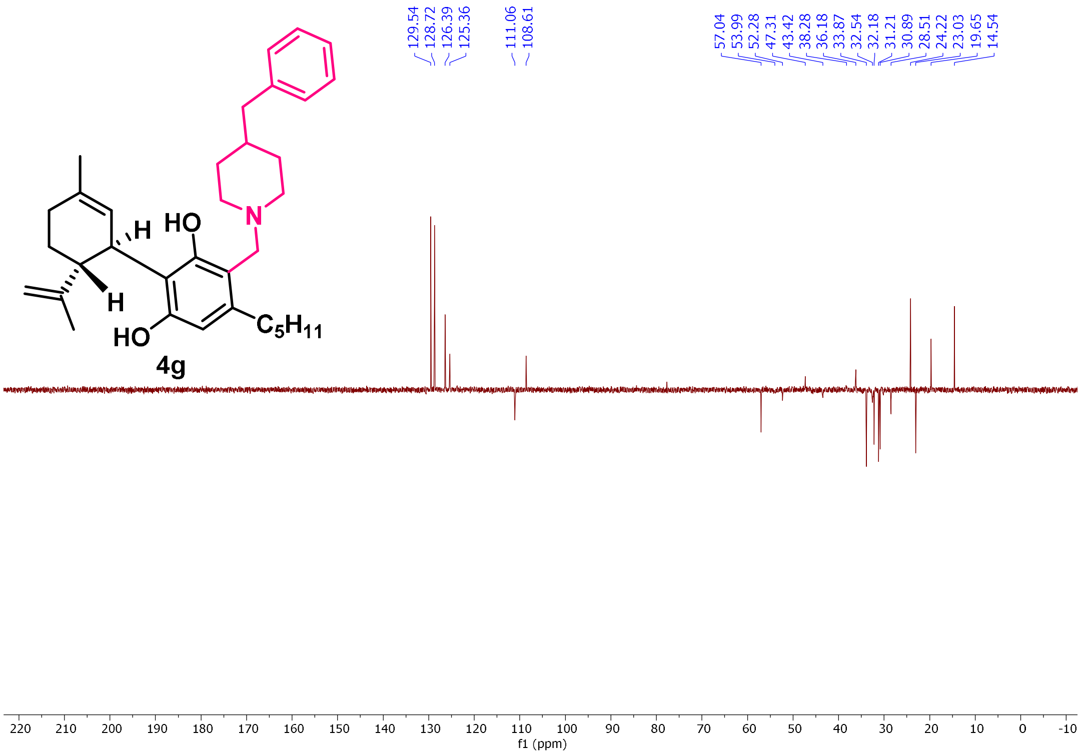

Figure S35. DEPT spectrum of **4g**

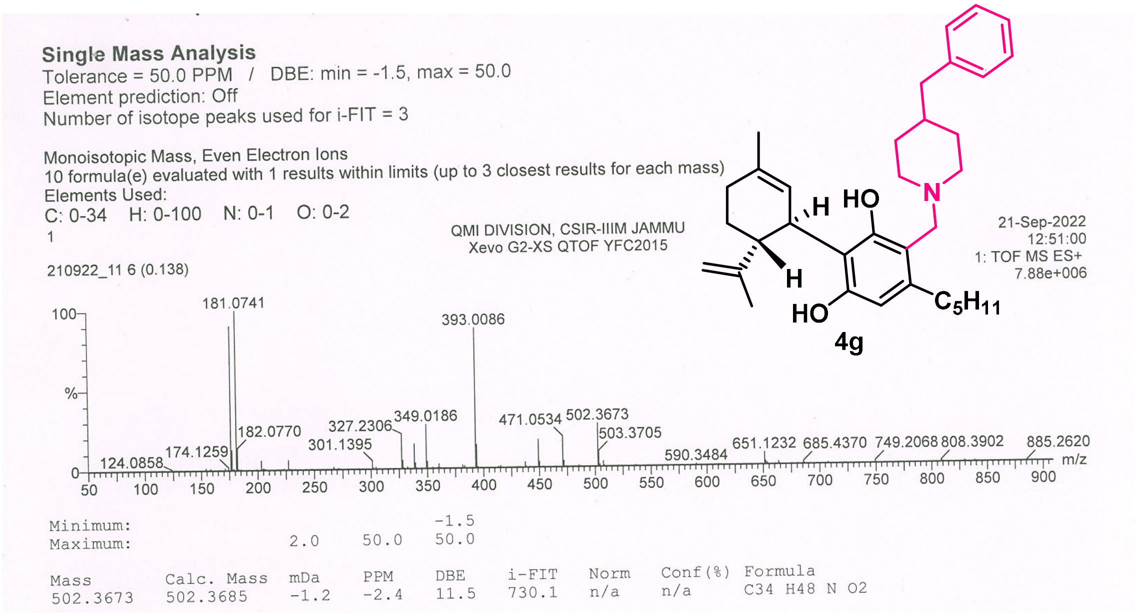

Figure S36. HRMS spectrum of **4g**

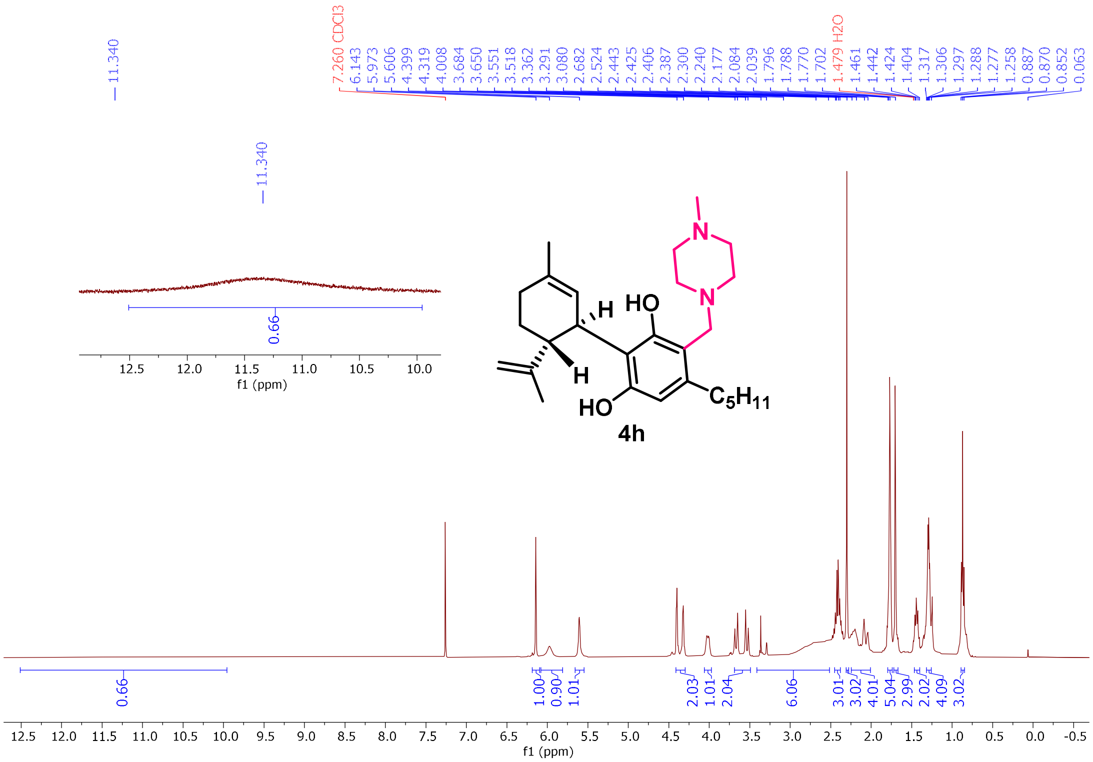

Figure S37. ^{1}H NMR spectrum of **4h**

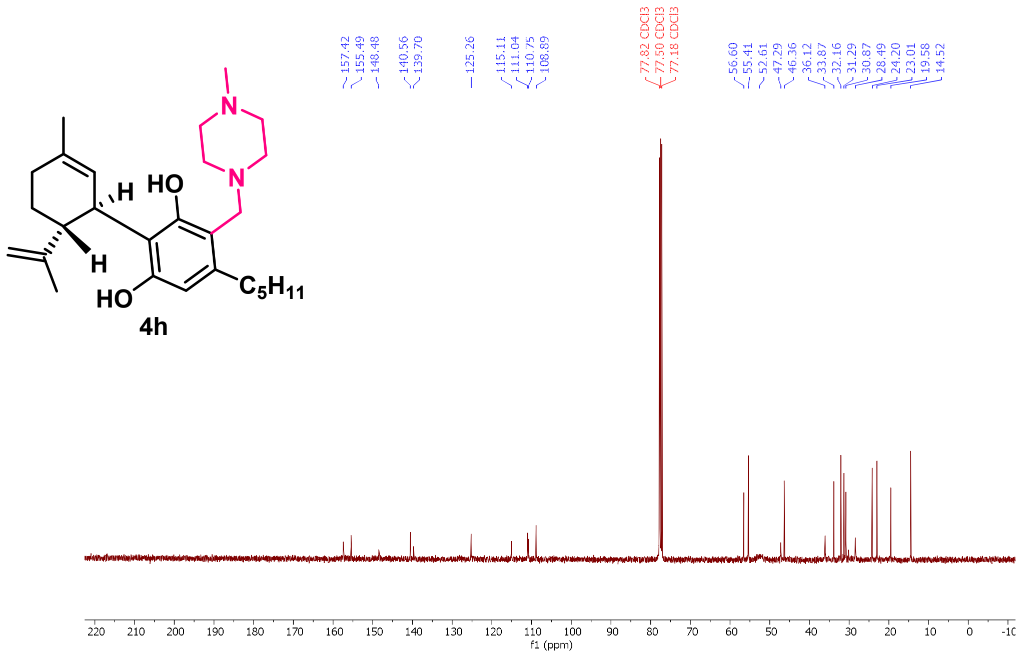

Figure S38. $^{13}C\{^{1}H\}$ NMR spectrum of **4h**

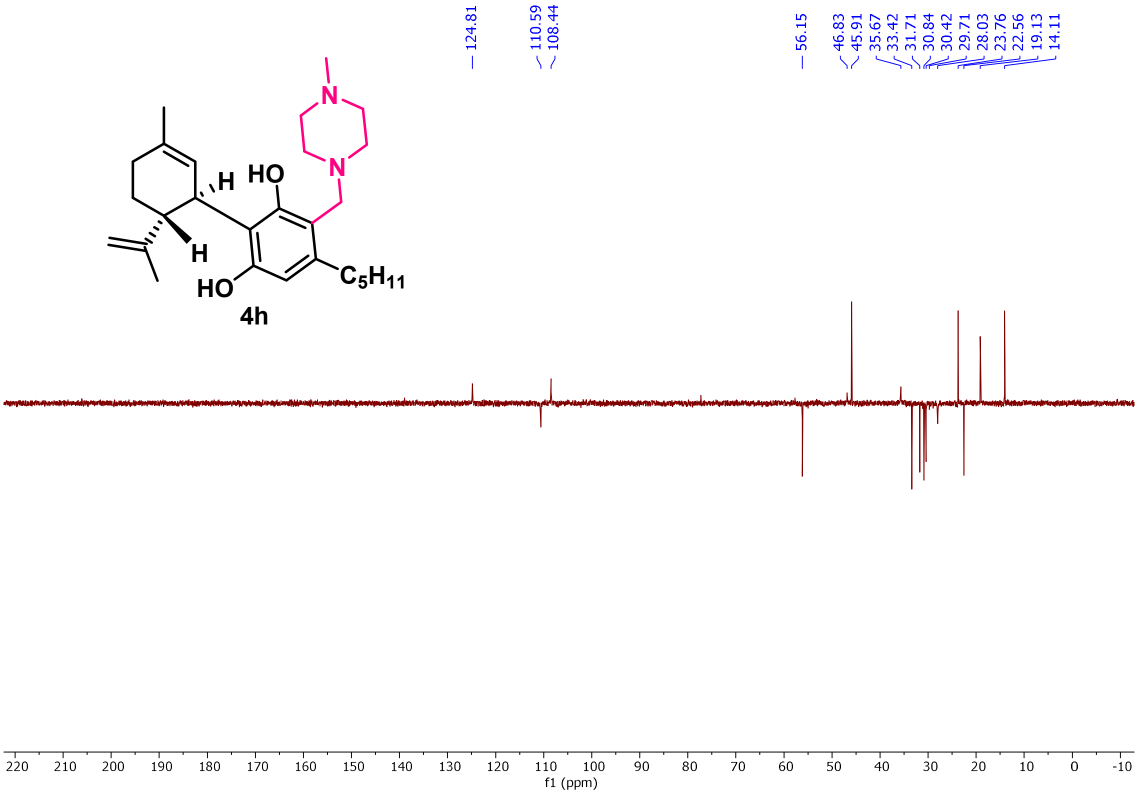

Figure S39. DEPT spectrum of **4h**

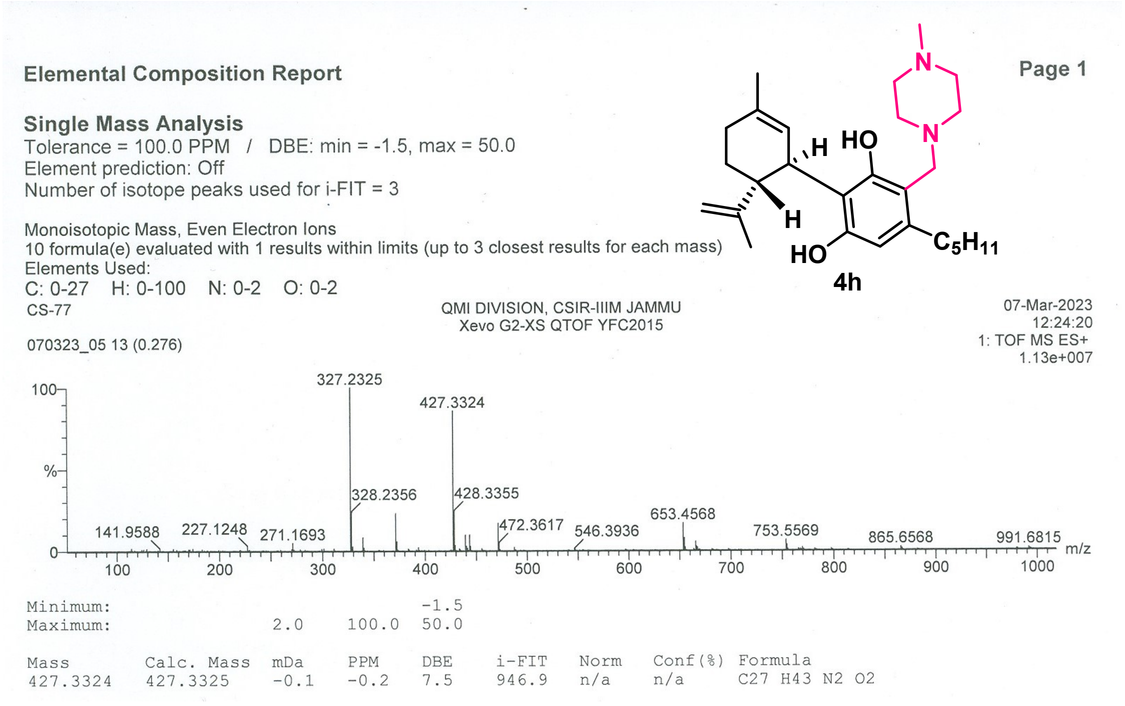

Figure S40. HRMS spectrum of **4h**

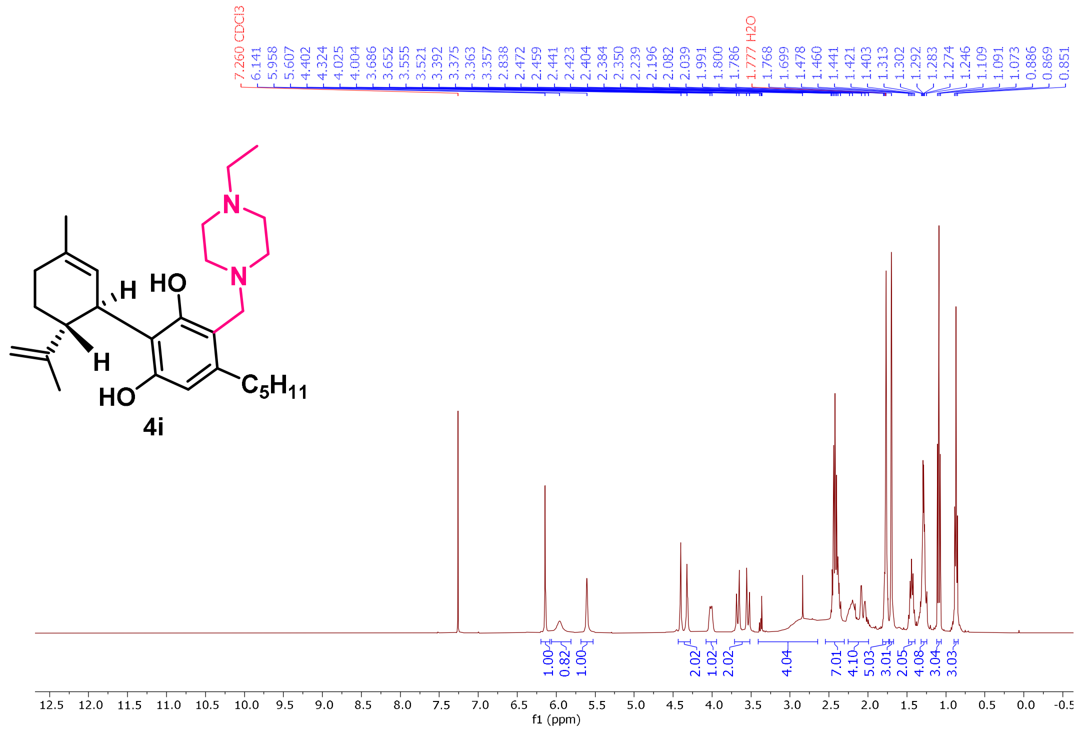

Figure S41. ^{1}H NMR spectrum of **4i**

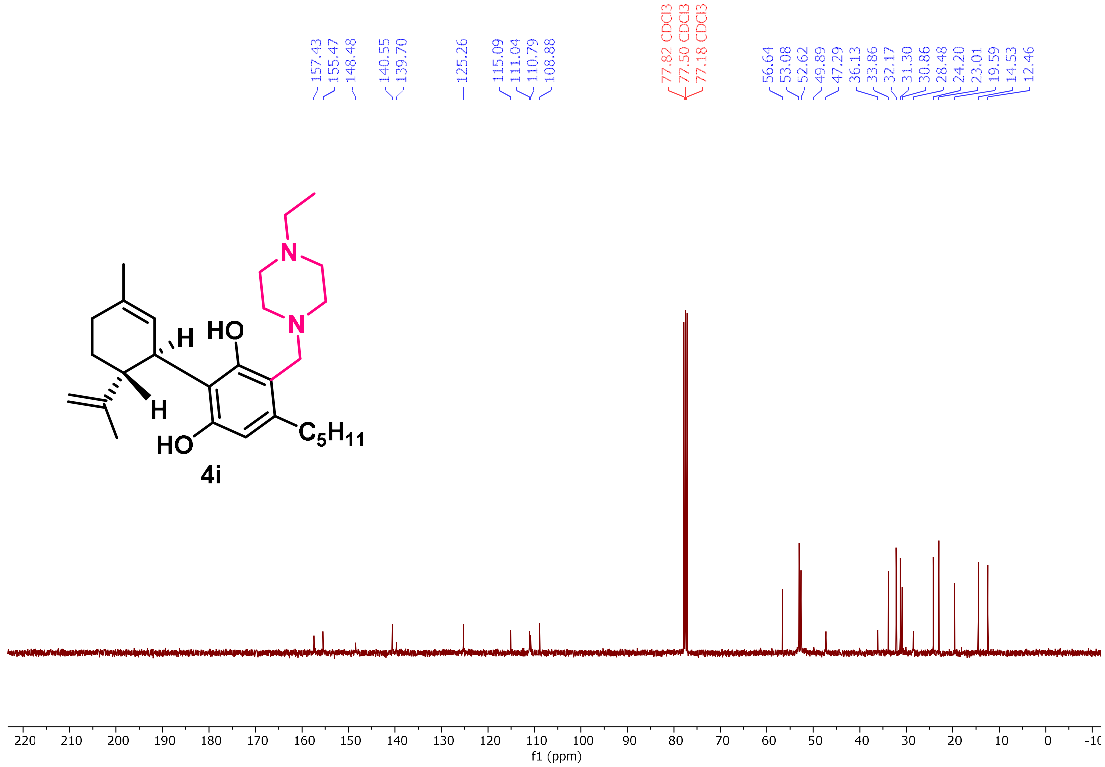

Figure S42. ^{13}C{^{1}H} NMR spectrum of **4i**

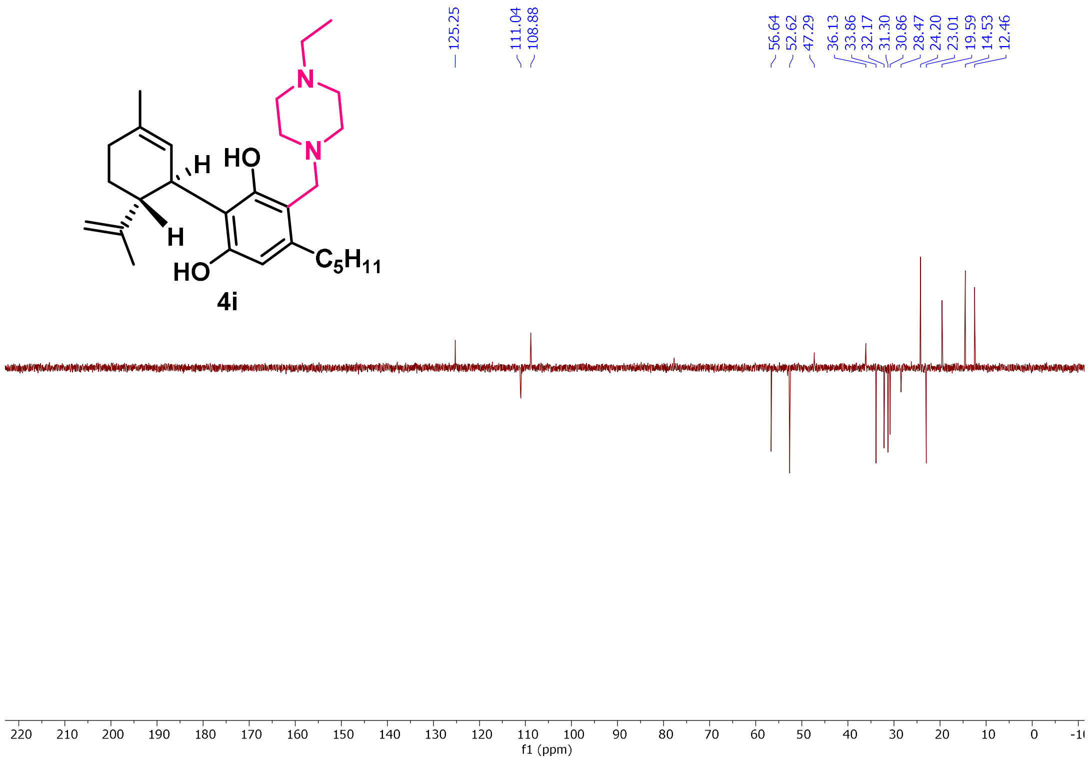

Figure S43. DEPT spectrum of **4i**

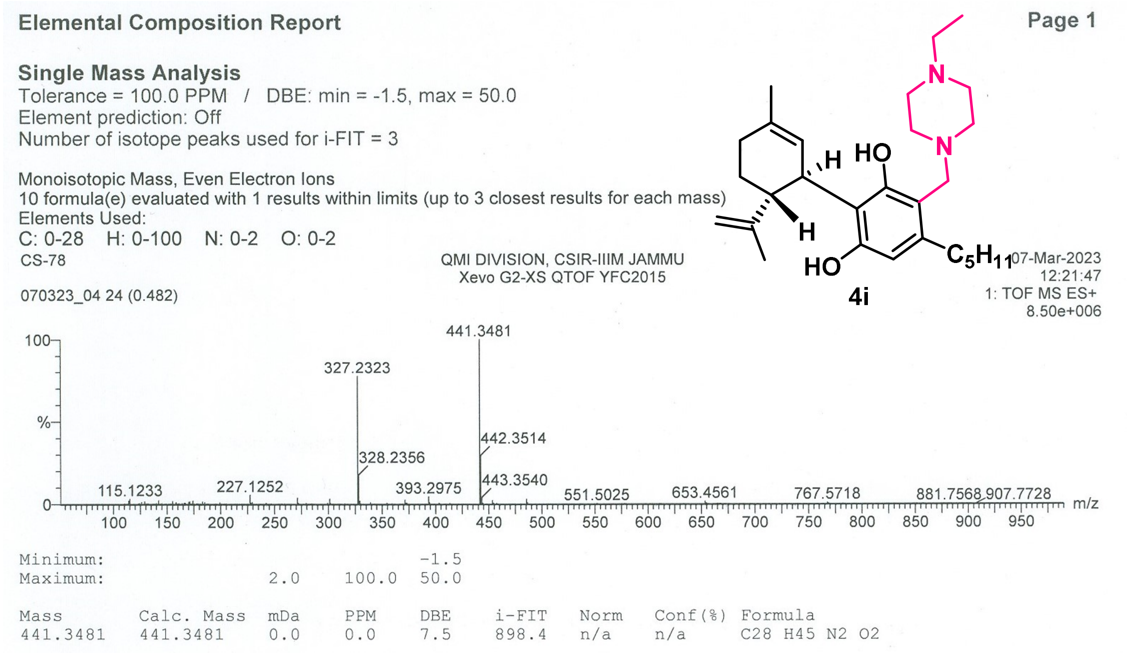

Elemental Composition Report — Page 1

Single Mass Analysis
Tolerance = 100.0 PPM / DBE: min = -1.5, max = 50.0
Element prediction: Off
Number of isotope peaks used for i-FIT = 3

Monoisotopic Mass, Even Electron Ions
10 formula(e) evaluated with 1 results within limits (up to 3 closest results for each mass)
Elements Used:
C: 0-28 H: 0-100 N: 0-2 O: 0-2

CS-78
QMI DIVISION, CSIR-IIIM JAMMU
Xevo G2-XS QTOF YFC2015
07-Mar-2023
12:21:47
070323_04 24 (0.482)
1: TOF MS ES+
8.50e+006

Minimum:				-1.5				
Maximum:		2.0	100.0	50.0				
Mass	Calc. Mass	mDa	PPM	DBE	i-FIT	Norm	Conf(%)	Formula
441.3481	441.3481	0.0	0.0	7.5	898.4	n/a	n/a	C28 H45 N2 O2

Figure S44. HRMS spectrum of **4i**

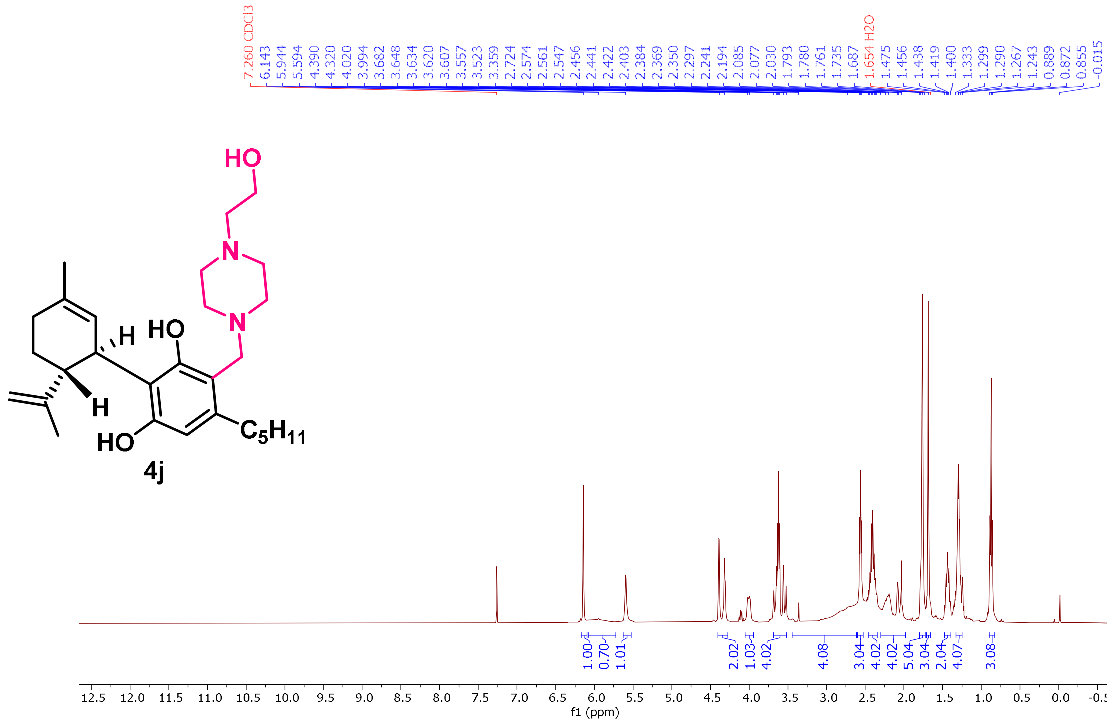

Figure S45. ^{1}H NMR spectrum of **4j**

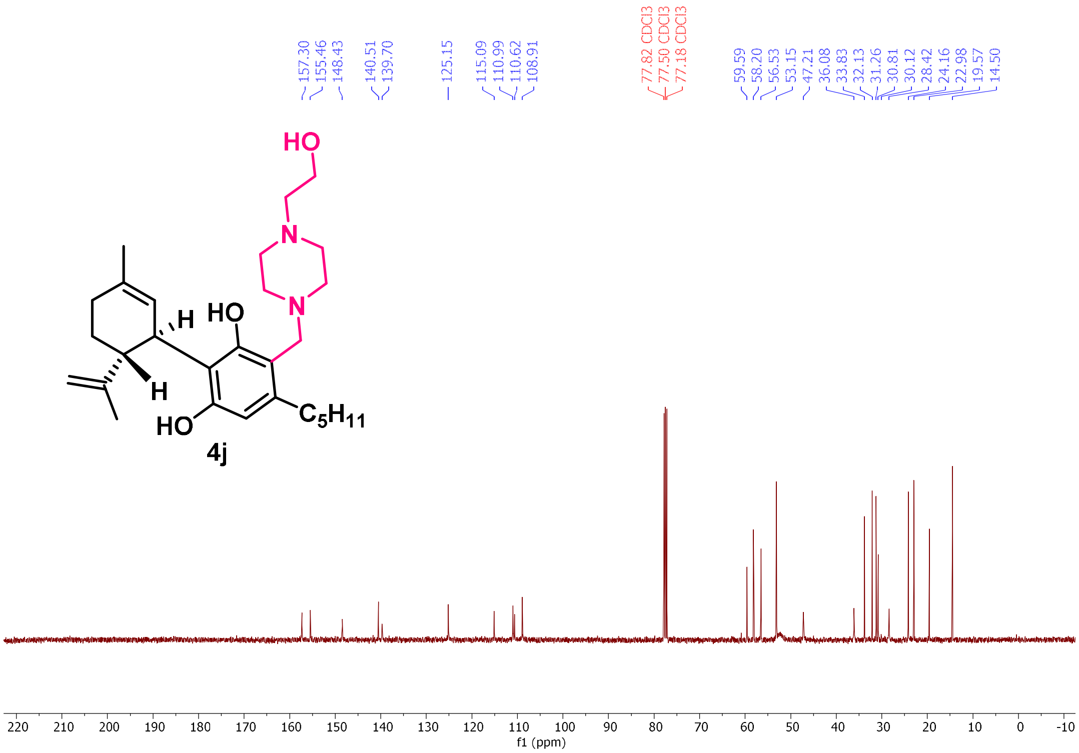

Figure S46. ^{13}C{^{1}H} NMR spectrum of **4j**

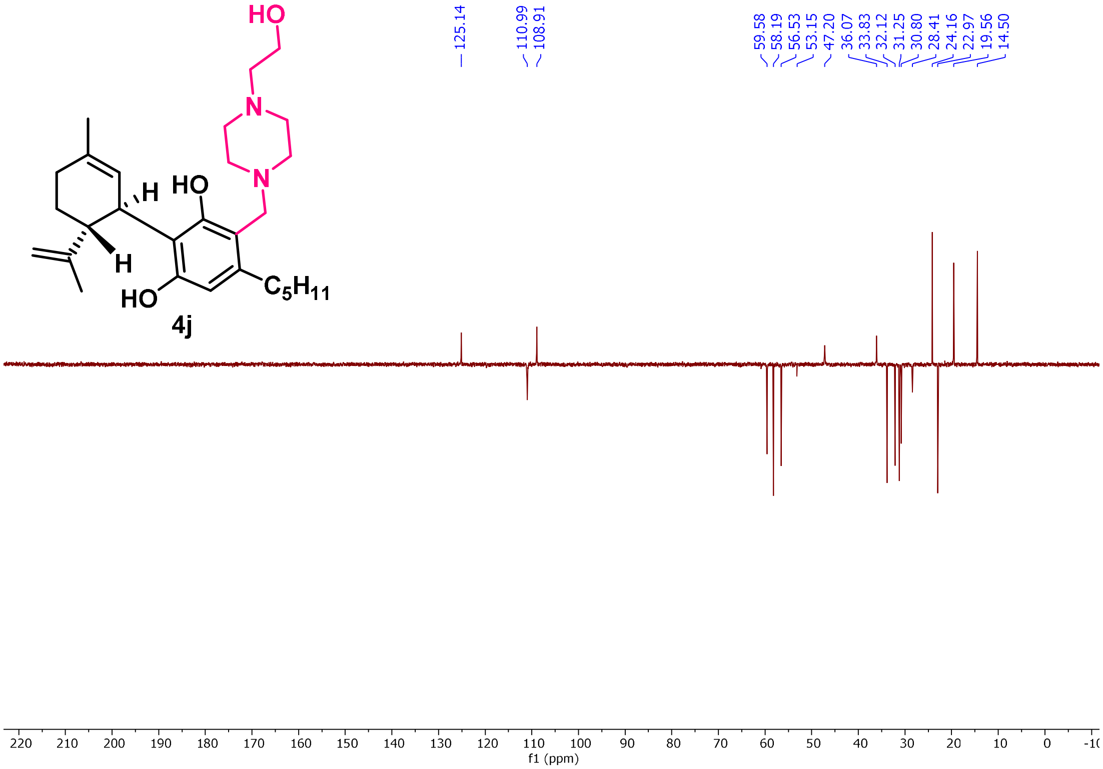

Figure S47. DEPT spectrum of **4j**

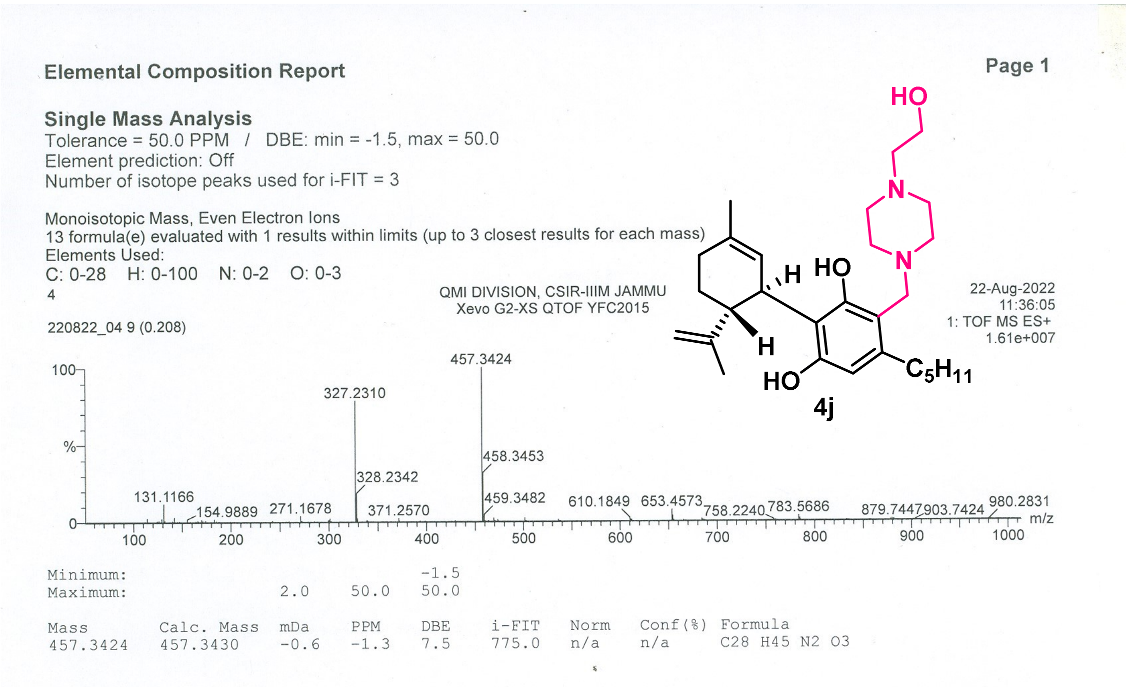

Figure S48. HRMS spectrum of **4j**

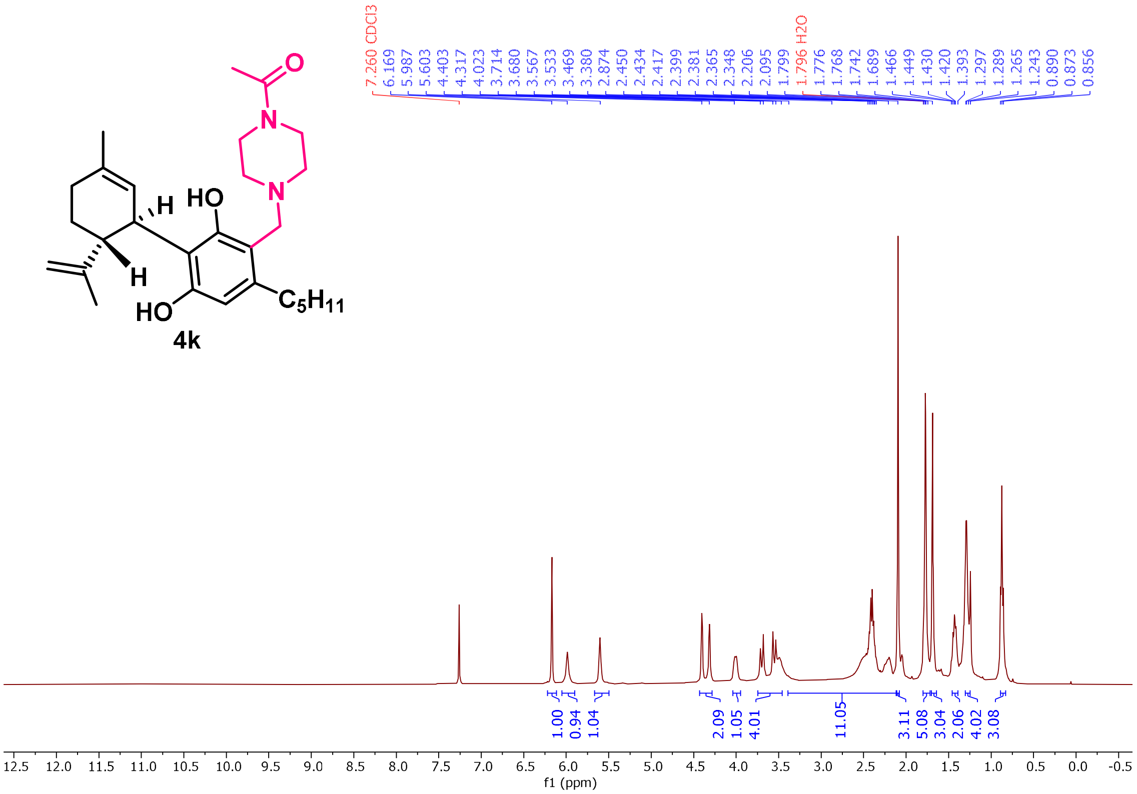

Figure S49. ^{1}H NMR spectrum of **4k**

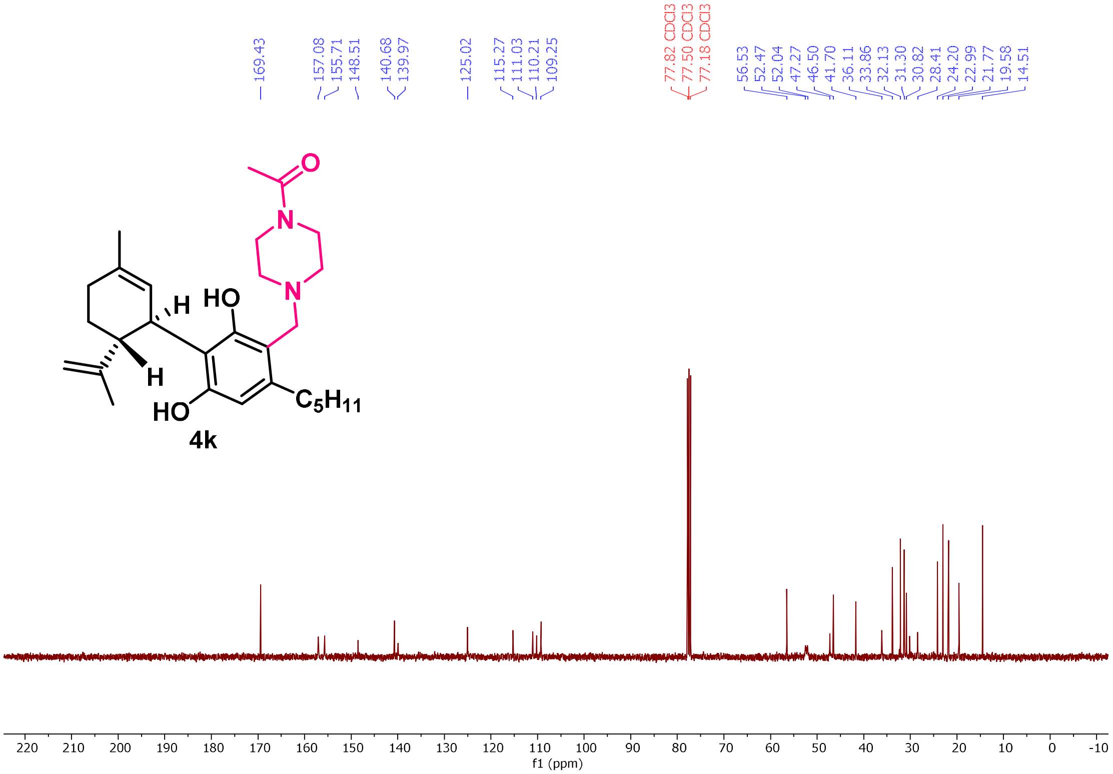

Figure S50. $^{13}C\{^1H\}$ NMR spectrum of **4k**

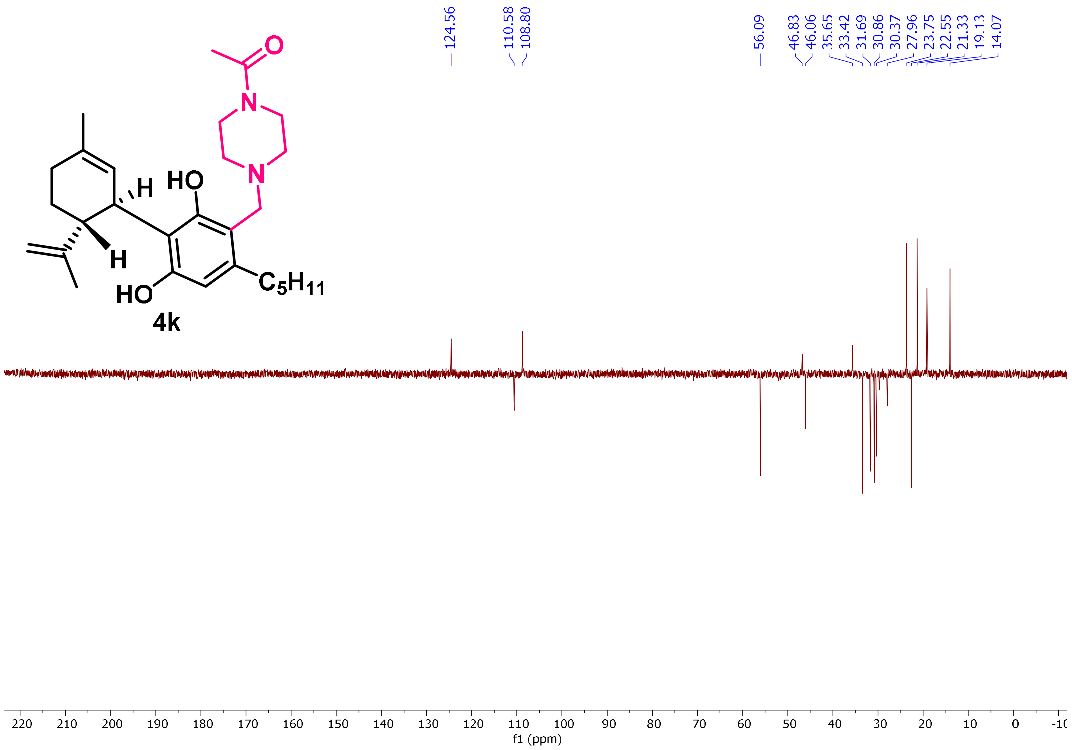

Figure S51. DEPT spectrum of **4k**

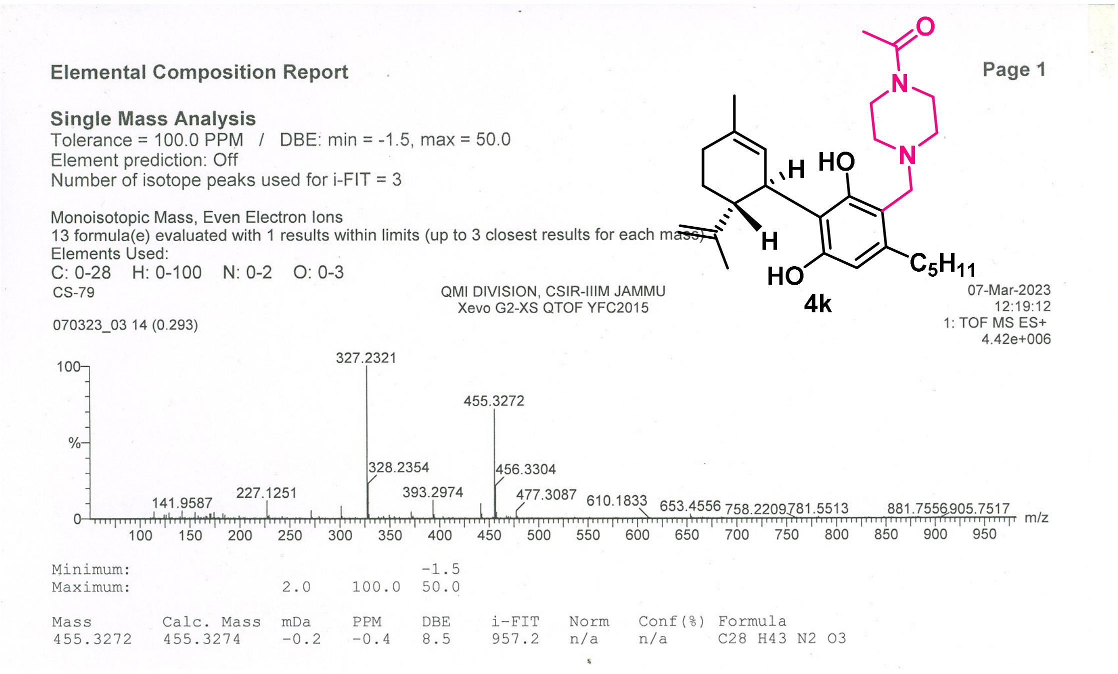

Figure S52. HRMS spectrum of **4k**

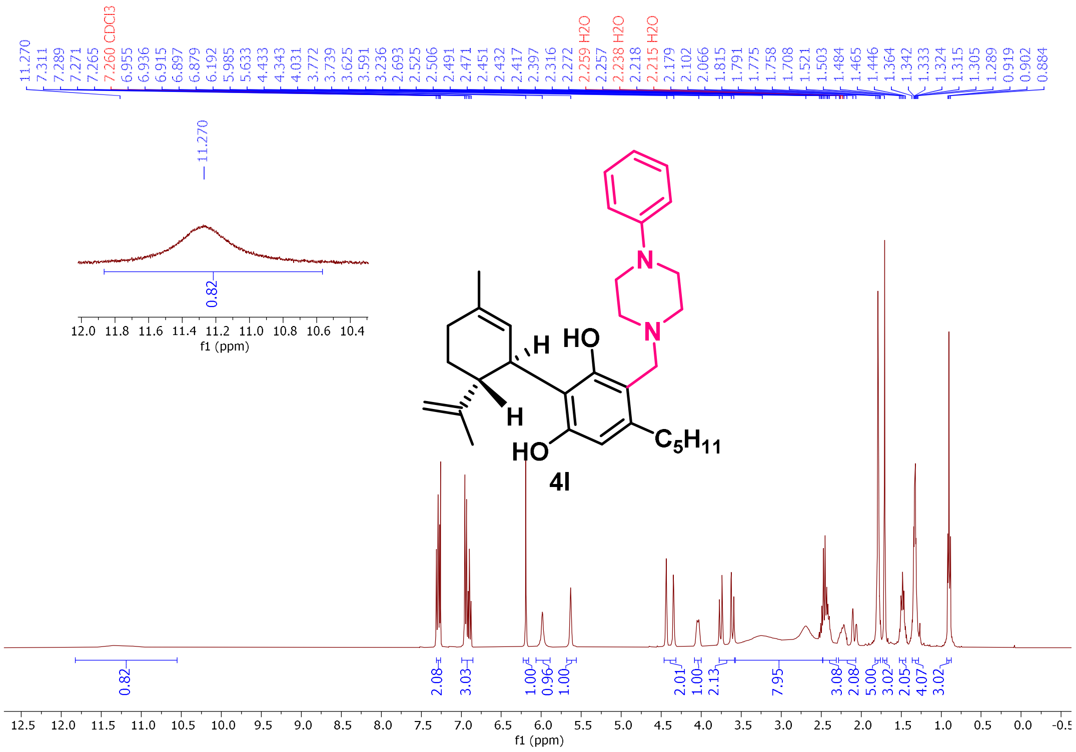

Figure S53. ^{1}H NMR spectrum of **4l**

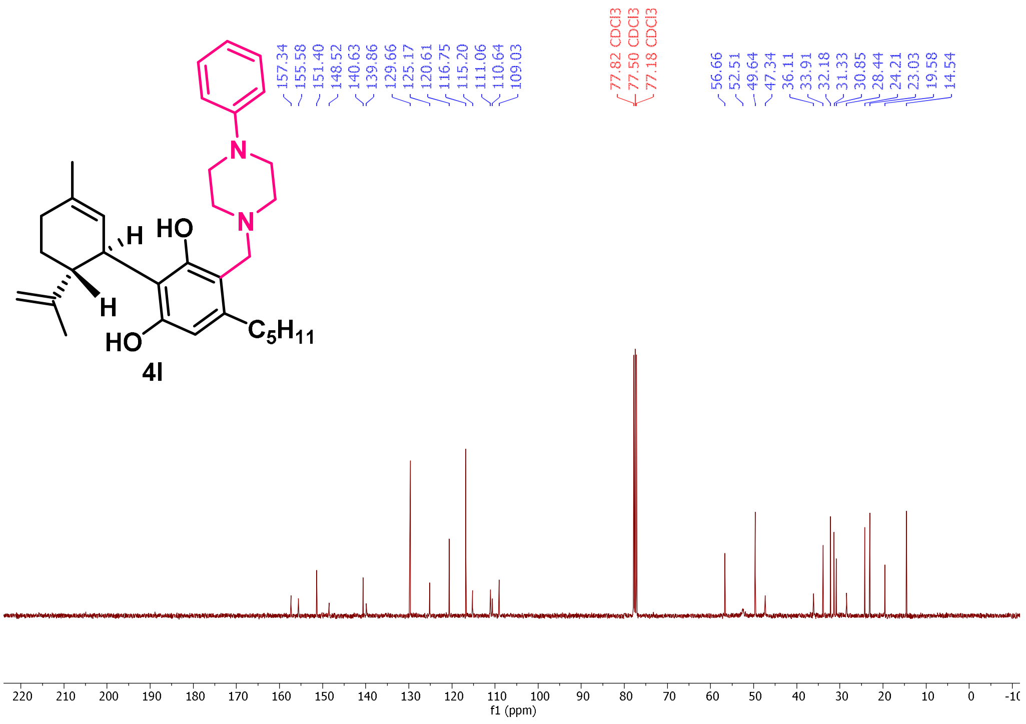

Figure S54. $^{13}C\{^1H\}$ NMR spectrum of **4l**

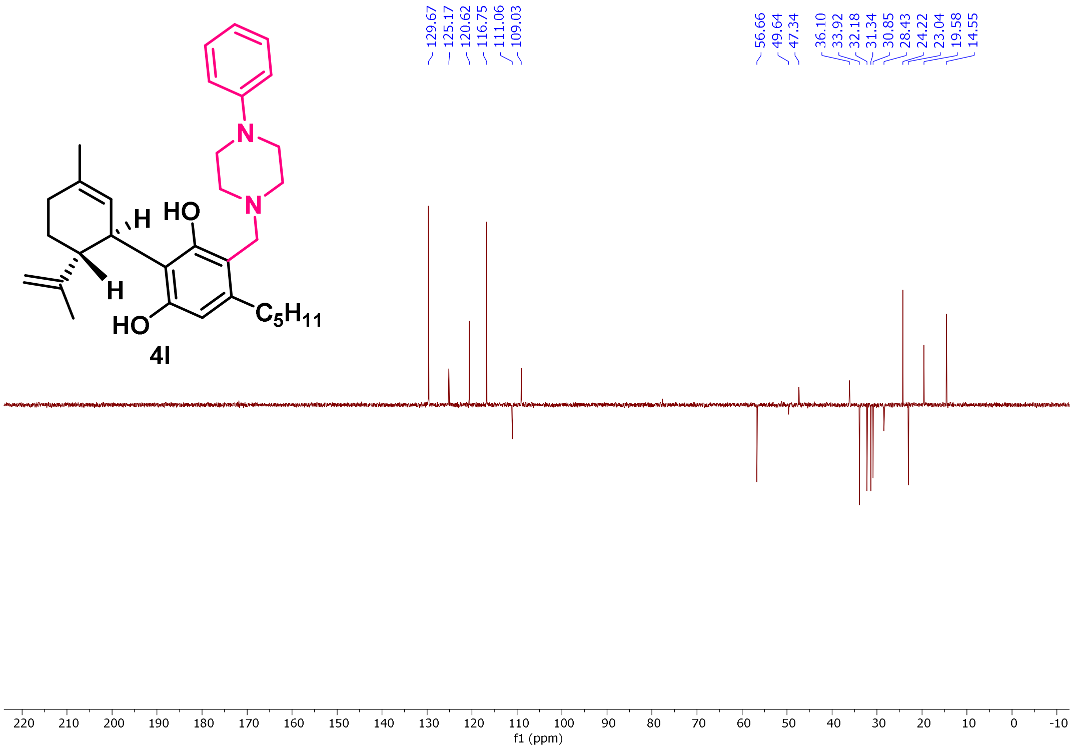

Figure S55. DEPT spectrum of **4l**

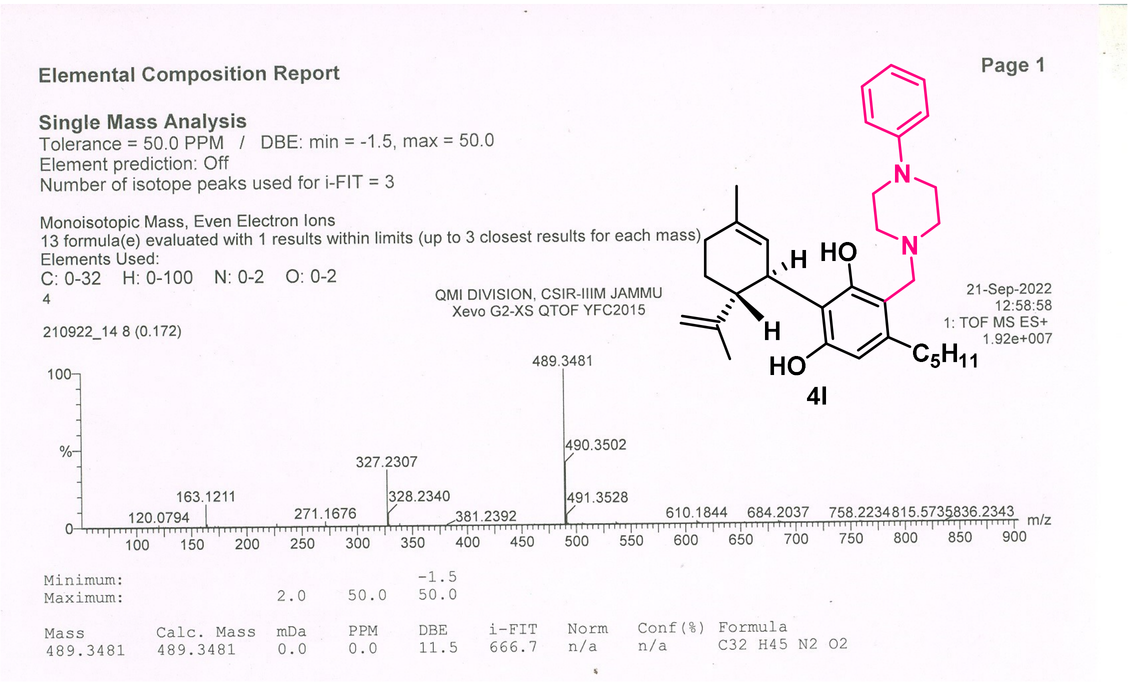

Figure S56. HRMS spectrum of **4l**

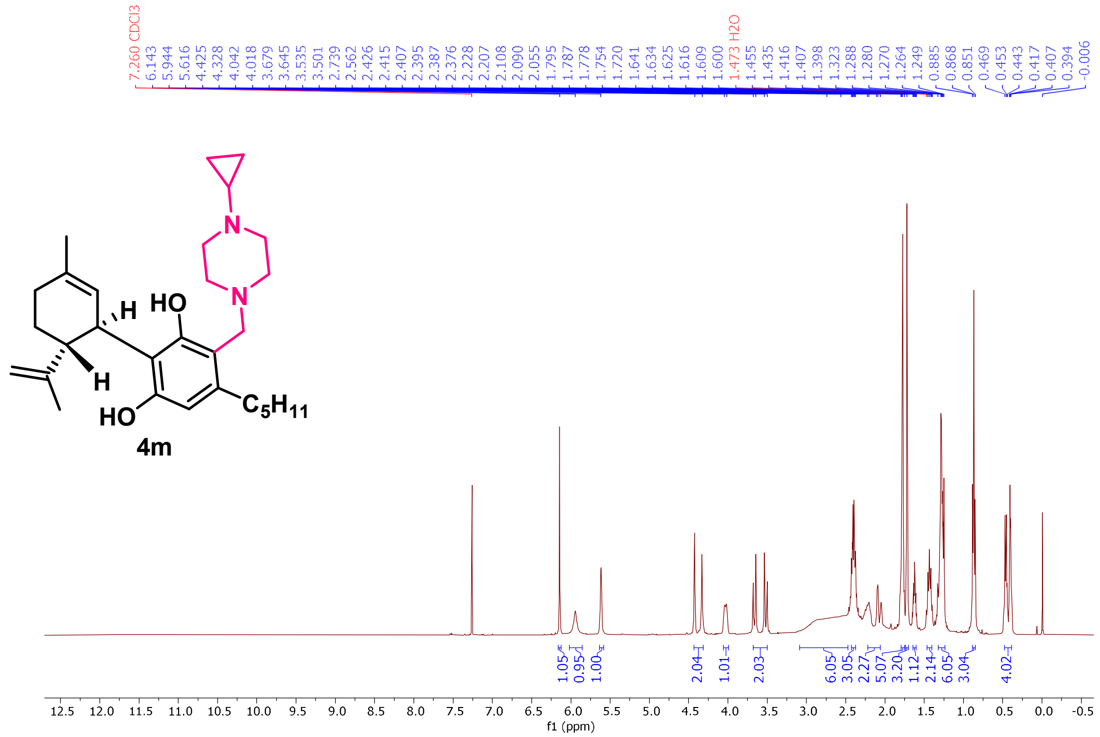

Figure S57. ^{1}H NMR spectrum of **4m**

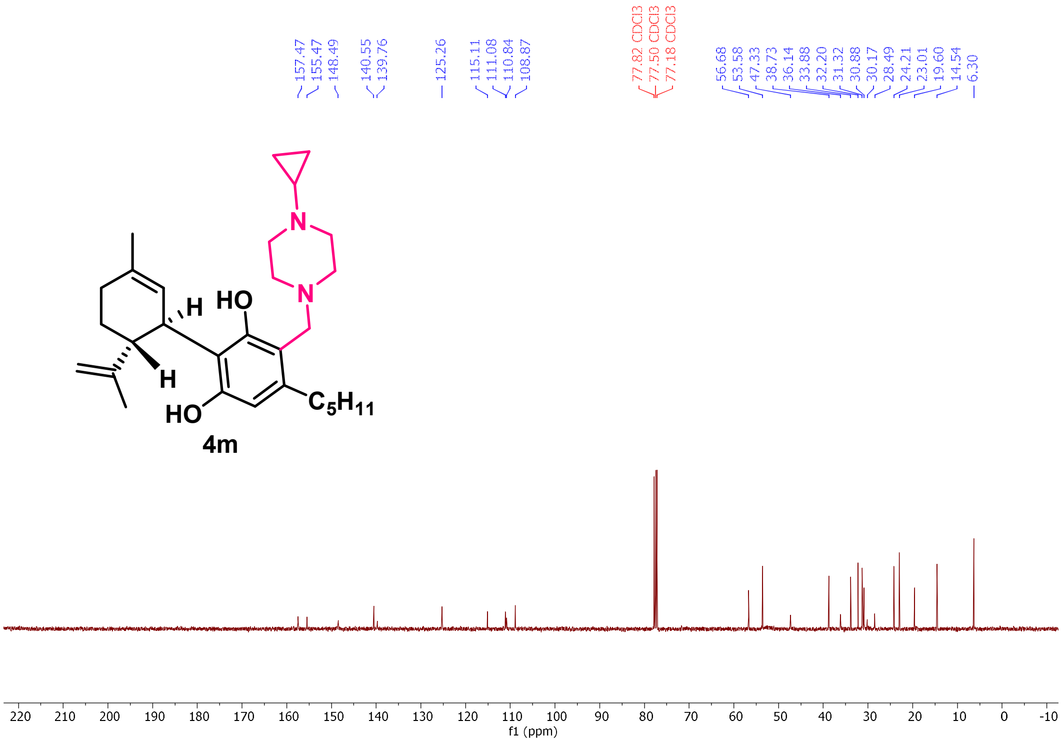

Figure S58. ^{13}C{^{1}H} NMR spectrum of **4m**

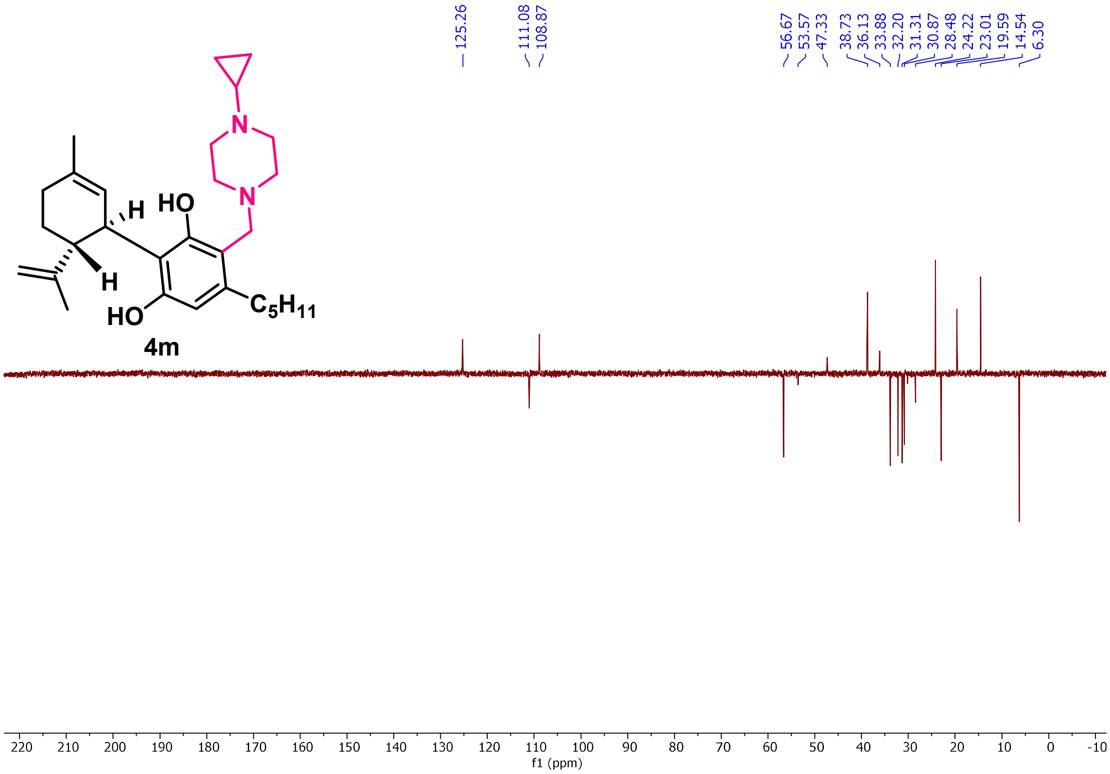

Figure S59. DEPT spectrum of **4m**

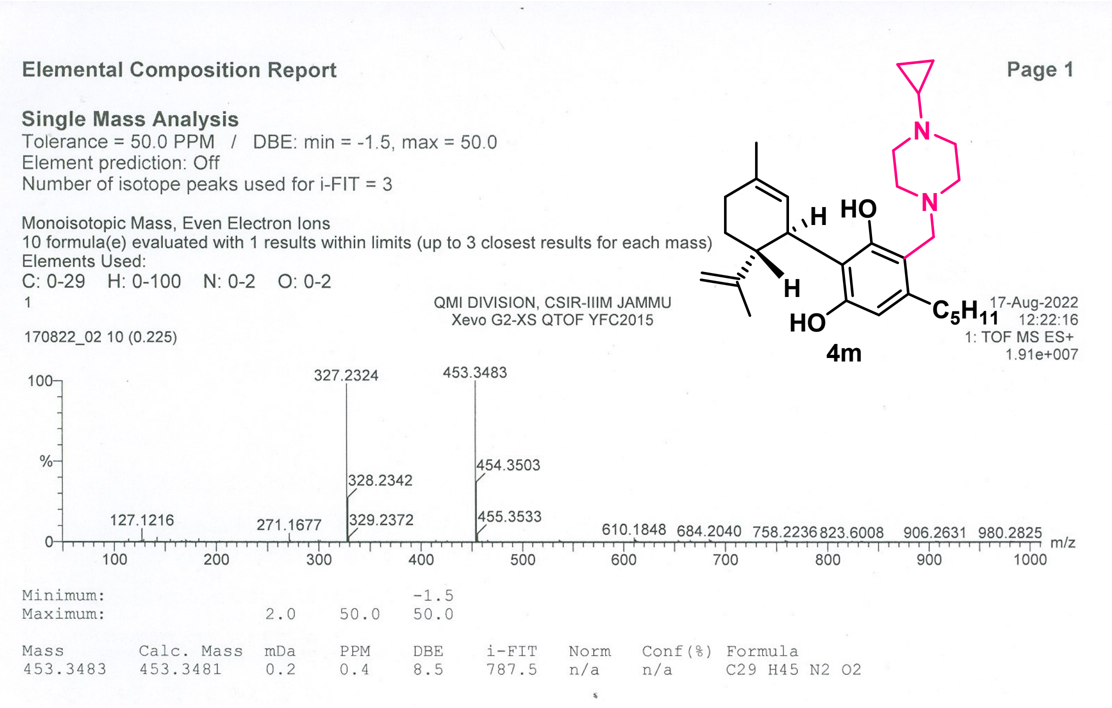

Figure S60. HRMS spectrum of **4m**

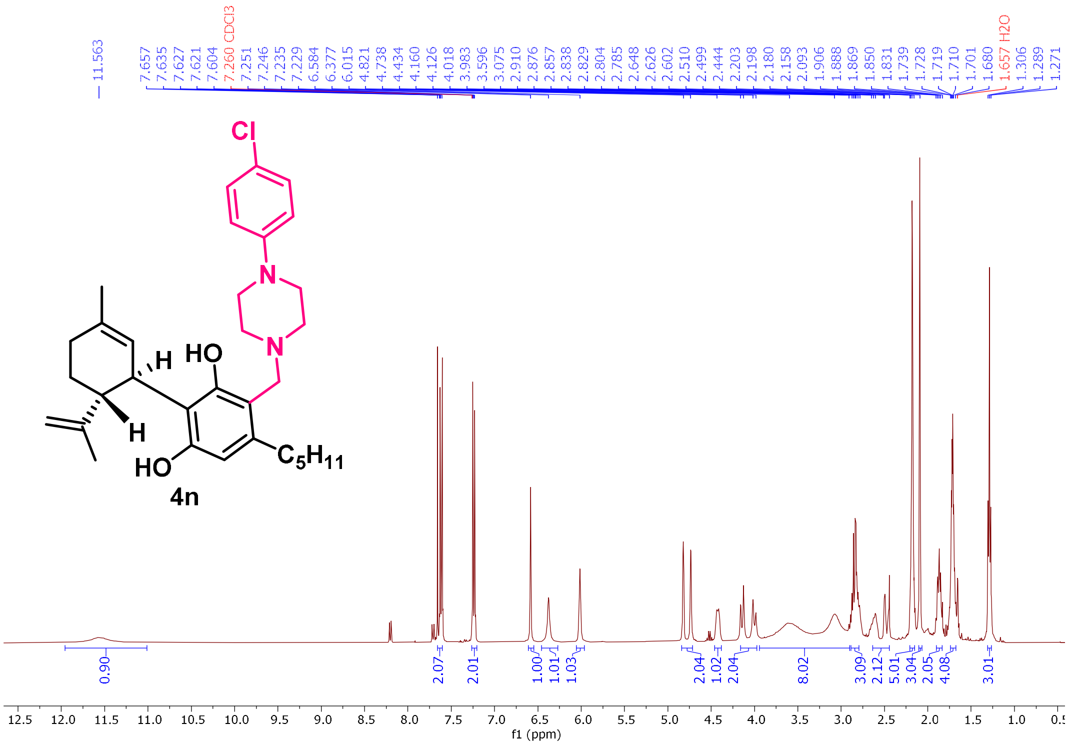

Figure S61. ^{1}H NMR spectrum of **4n**

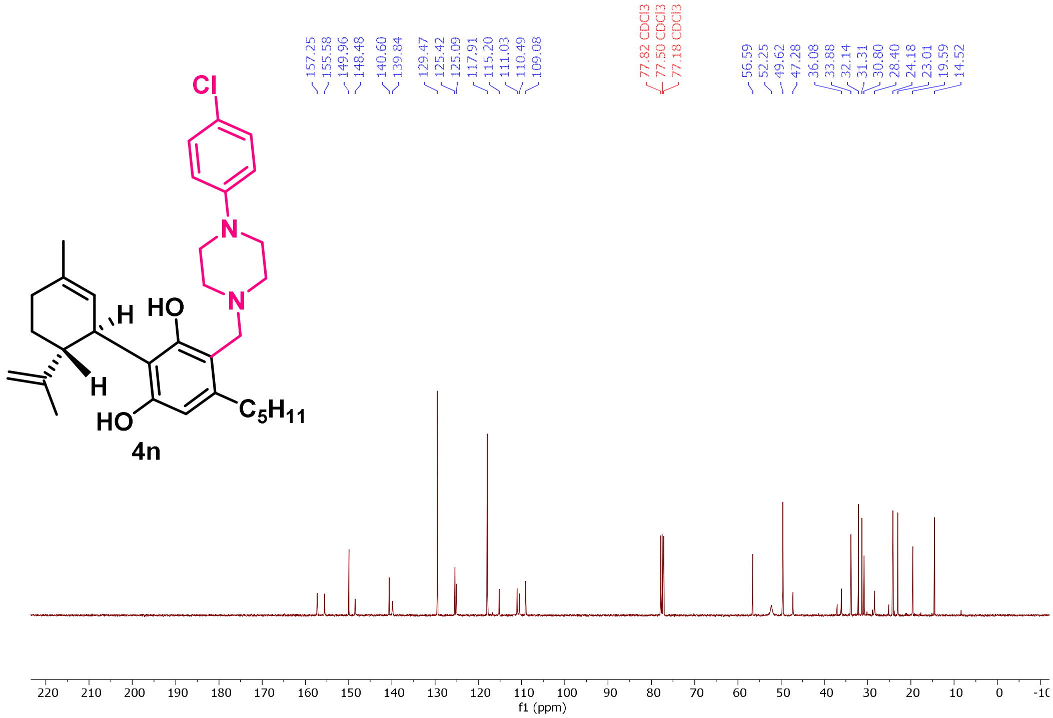

Figure S62. $^{13}C\{^{1}H\}$ NMR spectrum of **4n**

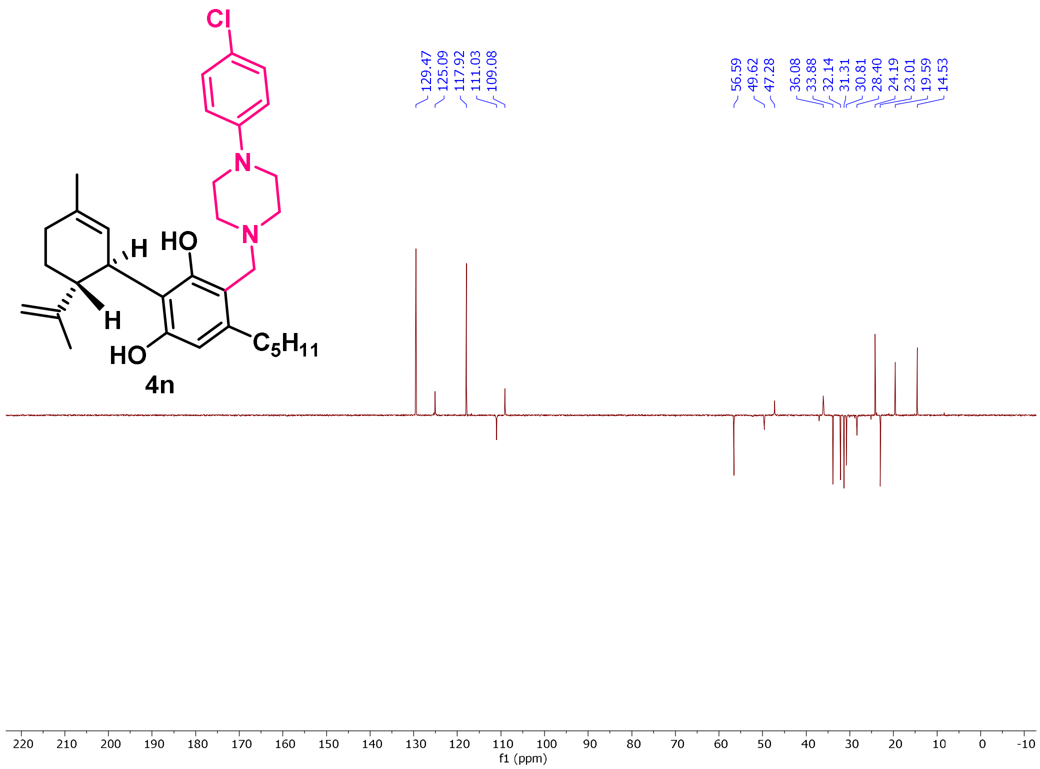

Figure S63. DEPT spectrum of **4n**

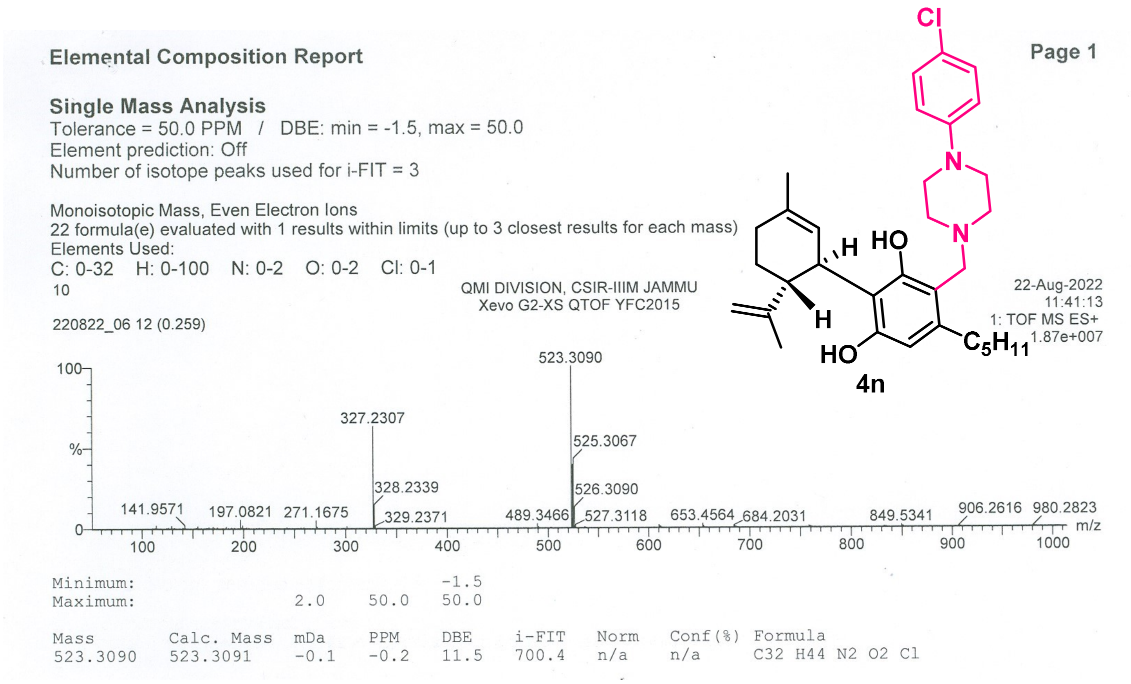

Figure S64. HRMS spectrum of **4n**

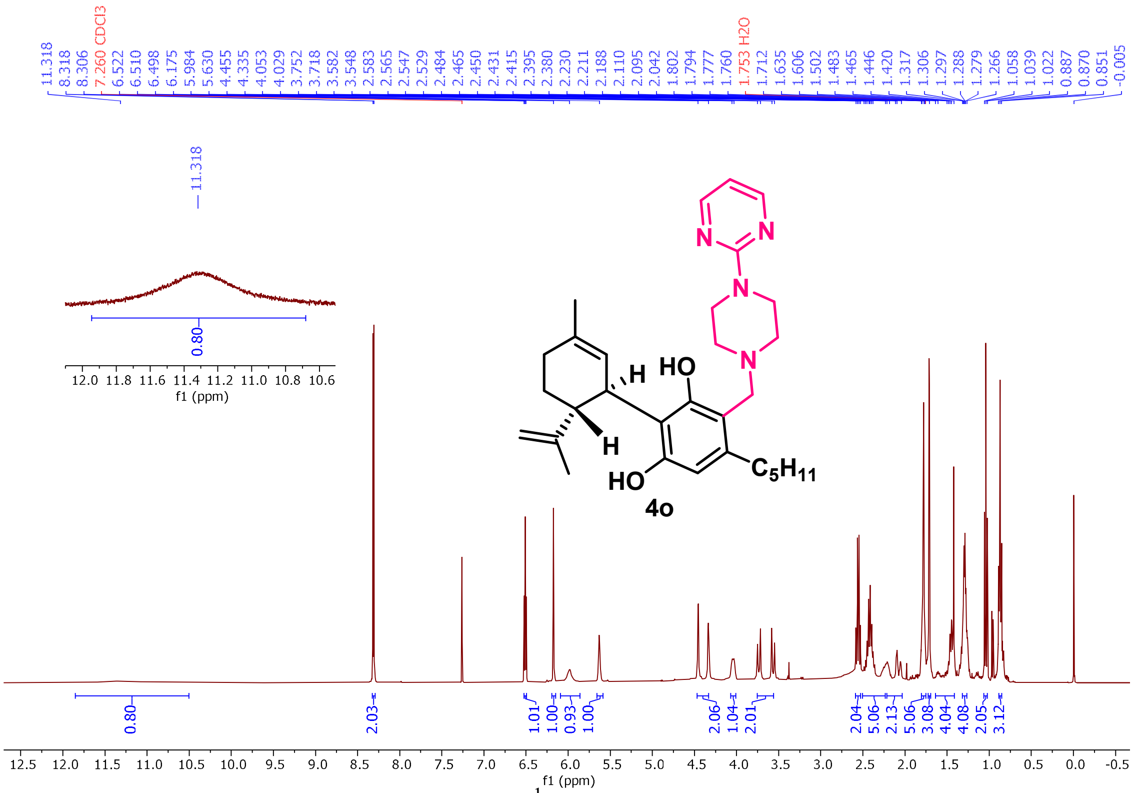

Figure S65. ^{1}H NMR spectrum of **4o**

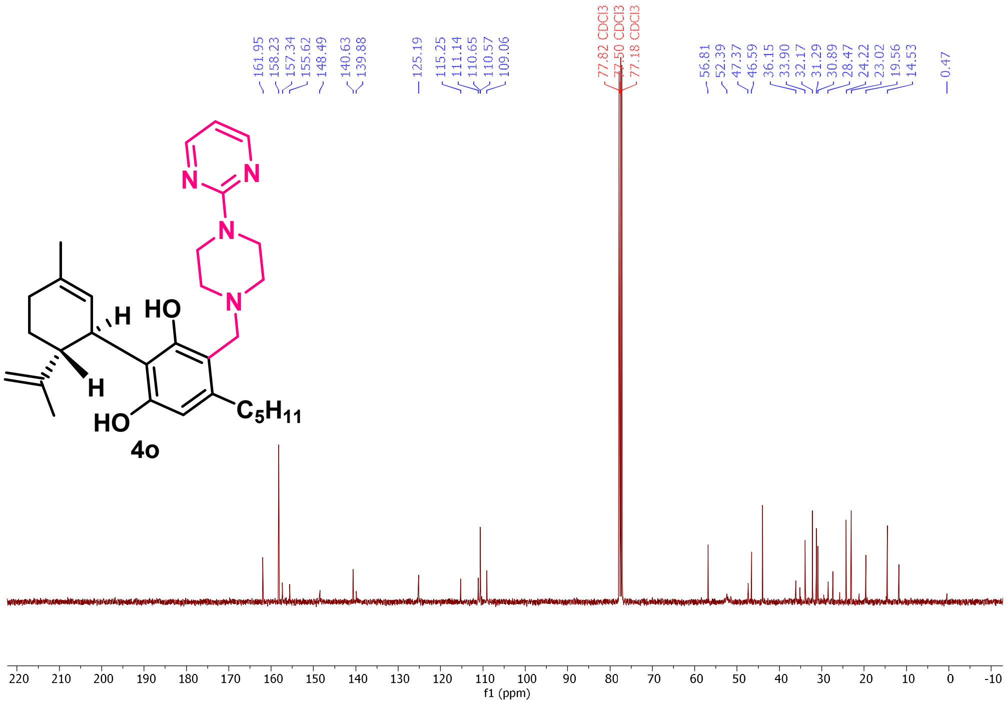

Figure S66. ^{13}C{^{1}H} NMR spectrum of **4o**

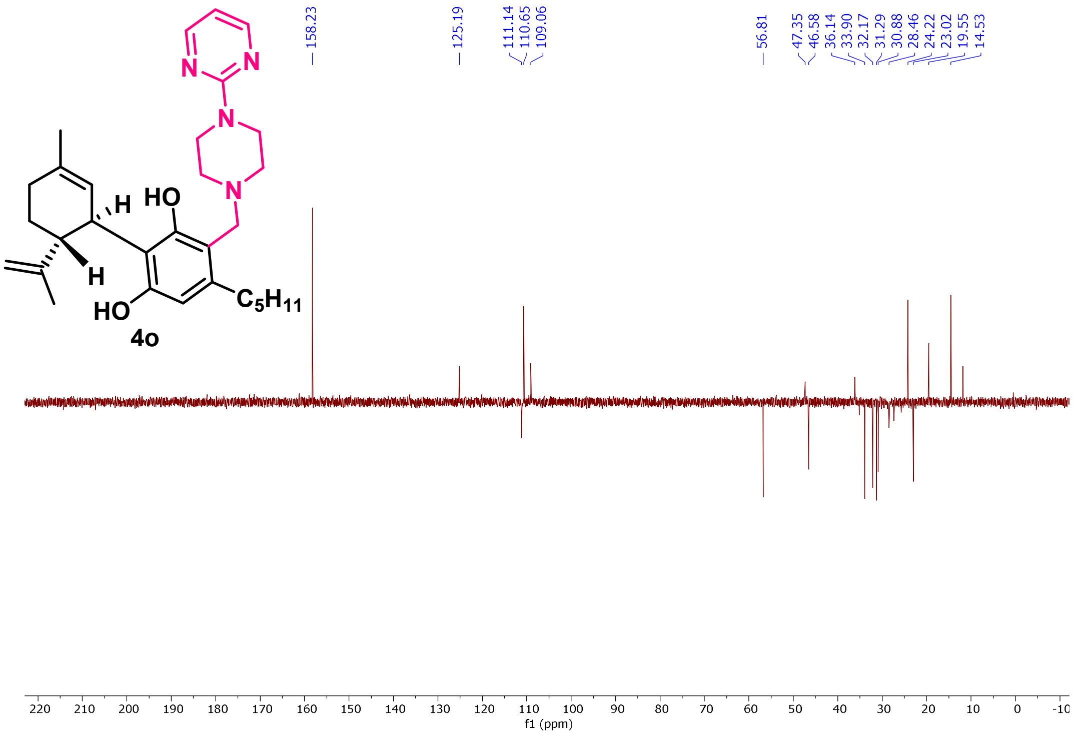

Figure S67. DEPT spectrum of **4o**

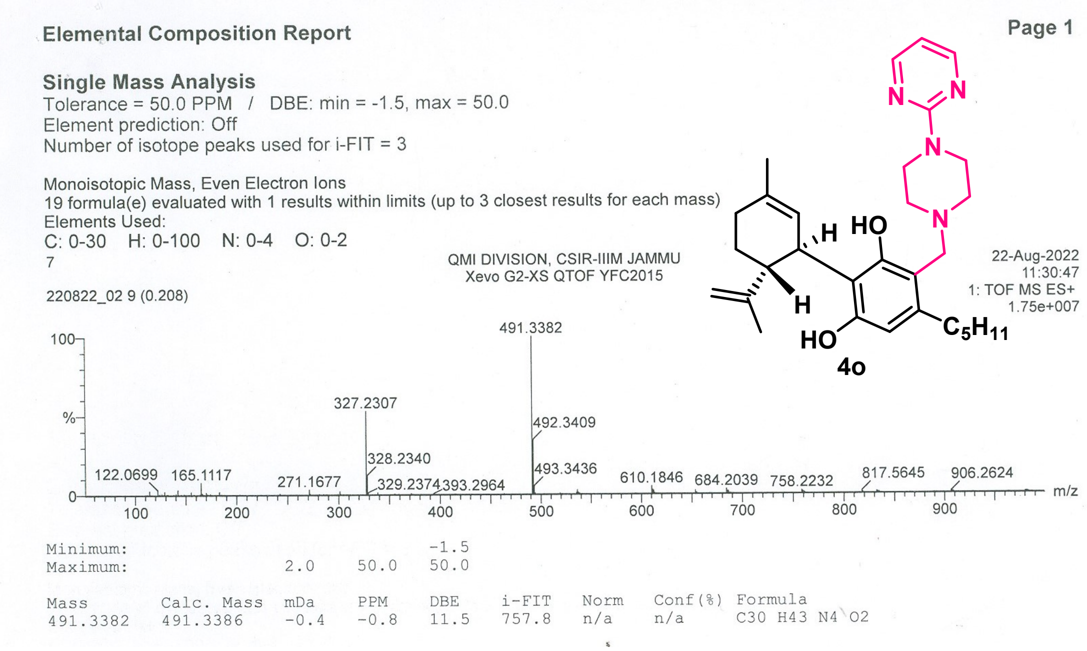

Figure S68. HRMS spectrum of **4o**

Molcular Docking studies

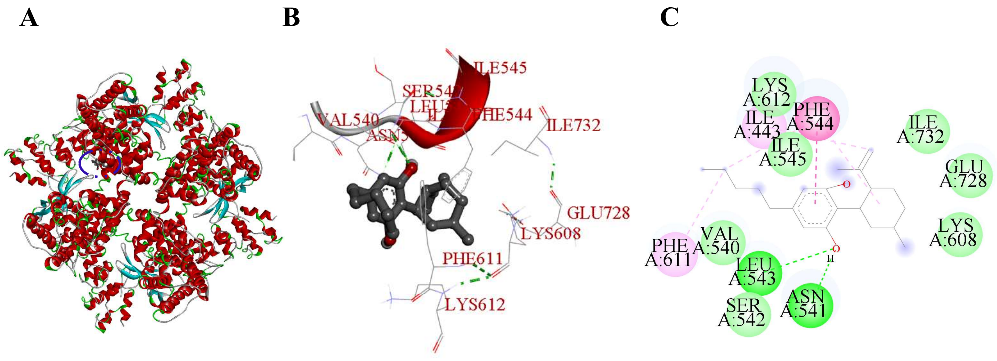

Figure S69 (a). Molecular docking interaction of **CBD-1** with 8T1F

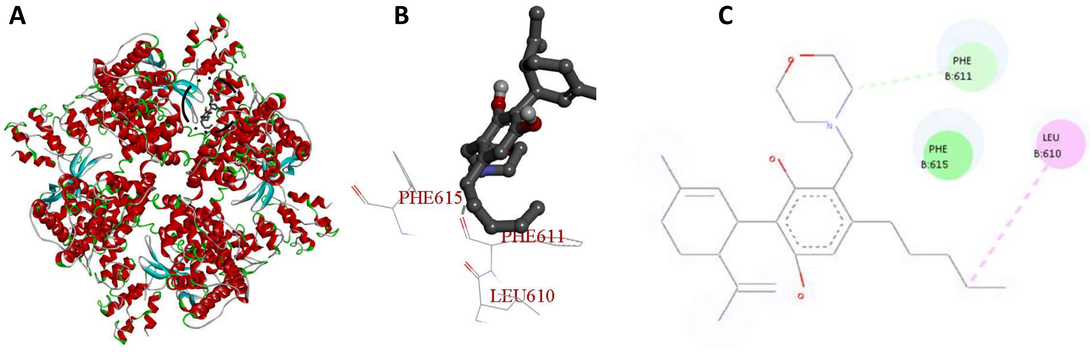

Figure S69 (b). Molecular docking interaction of **4a**

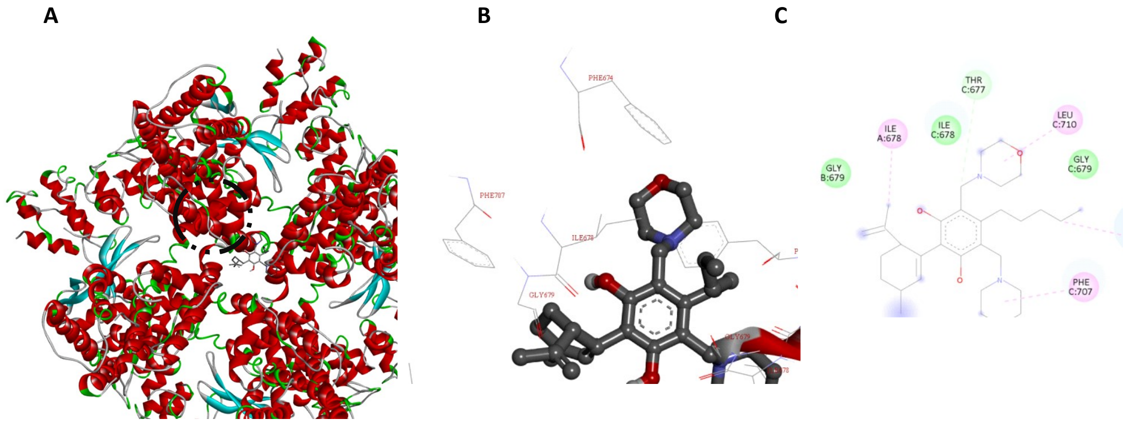

Figure S69 (c). Molecular docking interaction of **4a'**

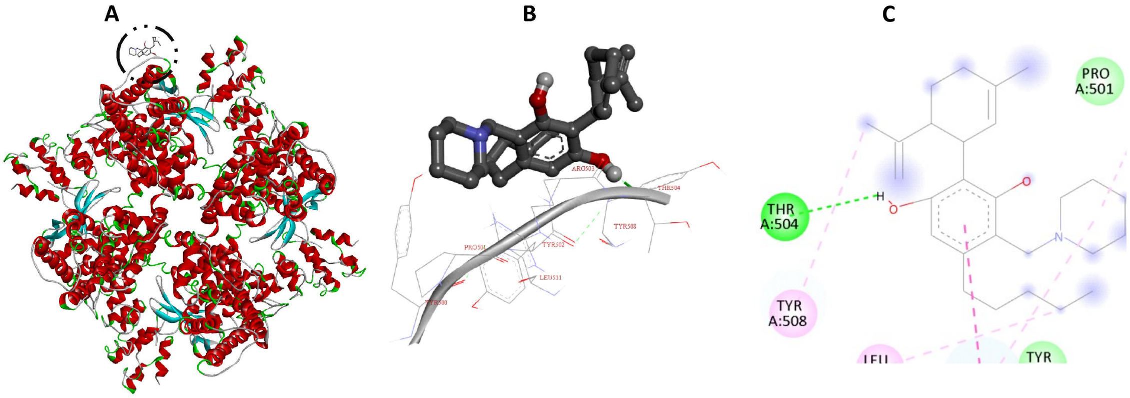

Figure S69 (d). Molecular docking interaction of **4b**

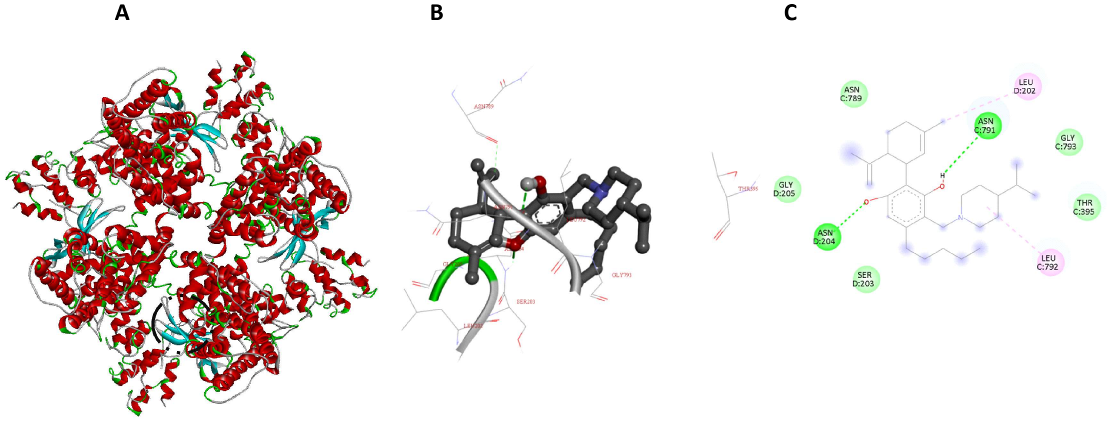

Figure S69 (e). Molecular docking interaction of **4c**

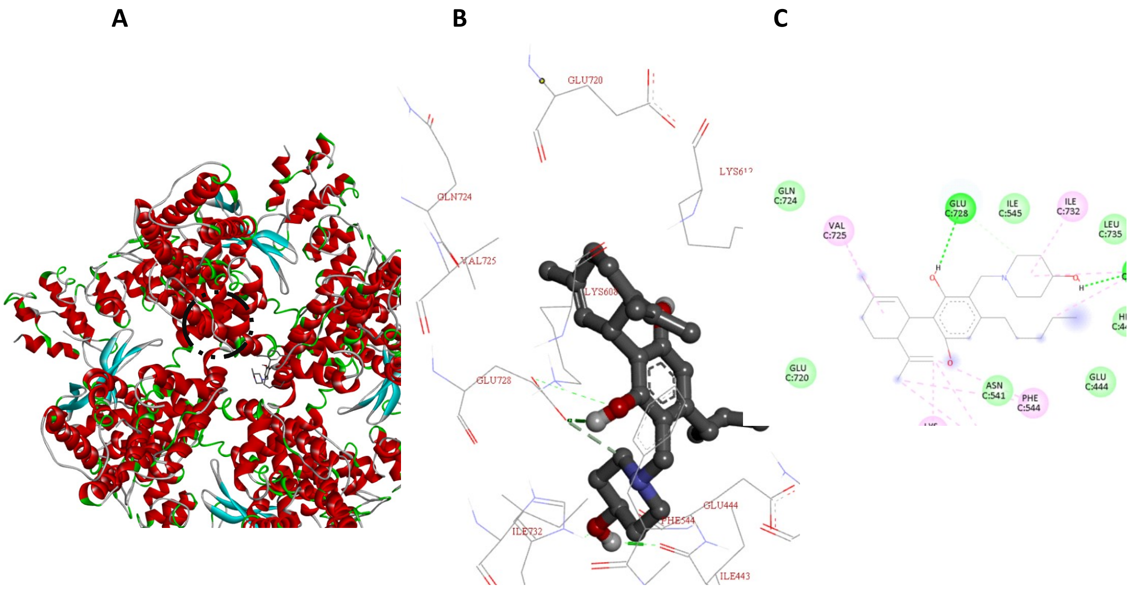

Figure S69 (f). Molecular docking interaction of **4d**

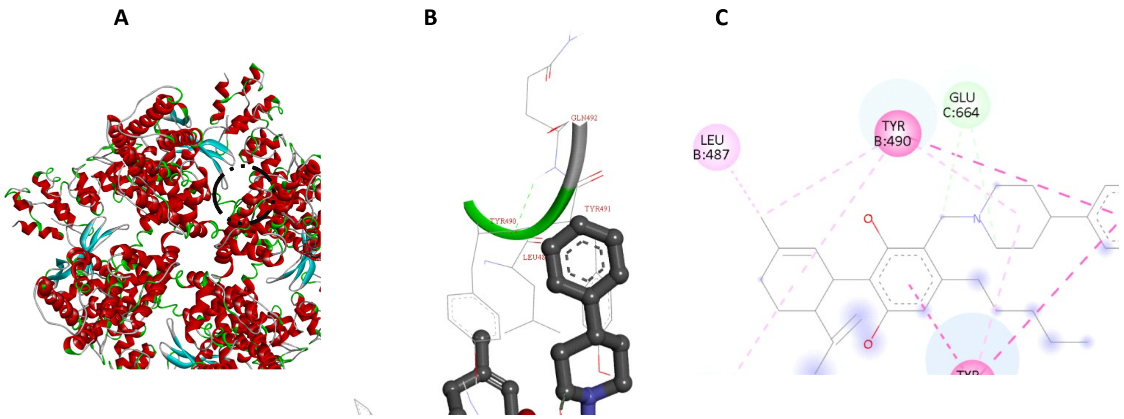

Figure S69 (g). Molecular docking interaction of **4e**

A B C

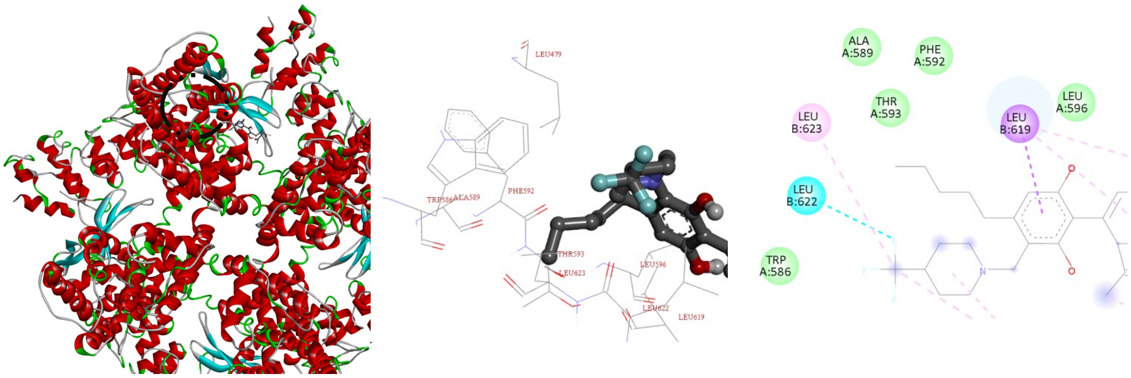

Figure S69 (h). Molecular docking interaction of **4f**

A B C

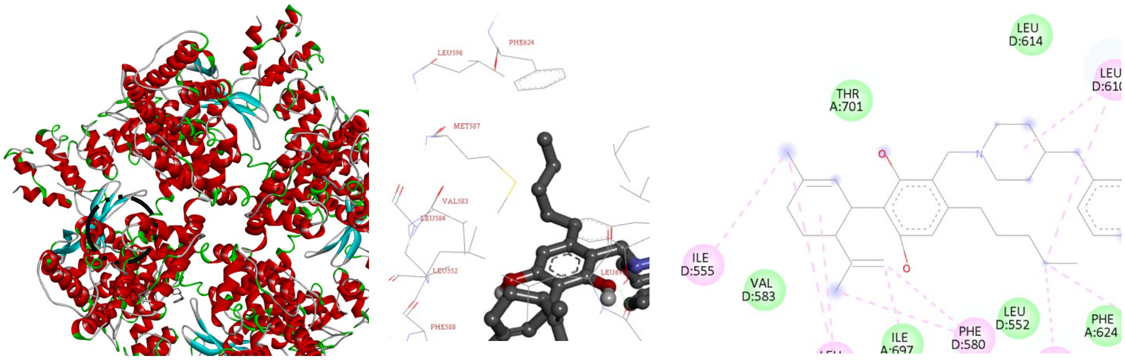

Figure S69 (i). Molecular docking interaction of **4g**

A B C

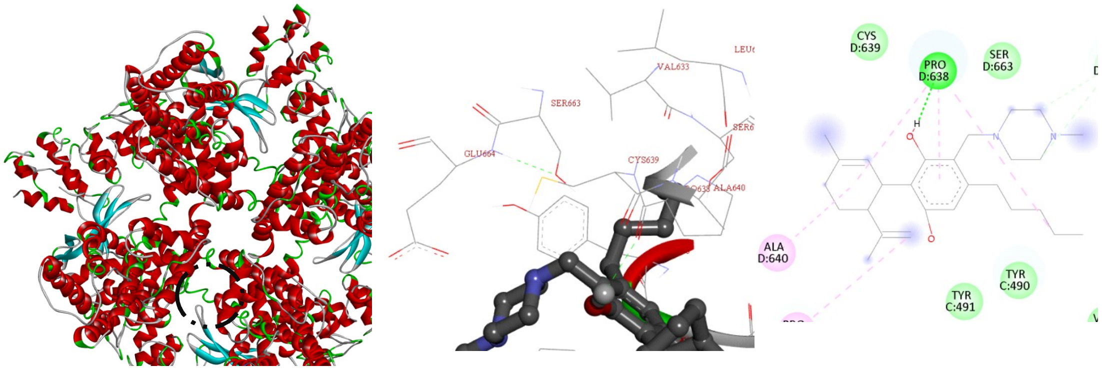

Figure S69 (j). Molecular docking interaction of **4h**

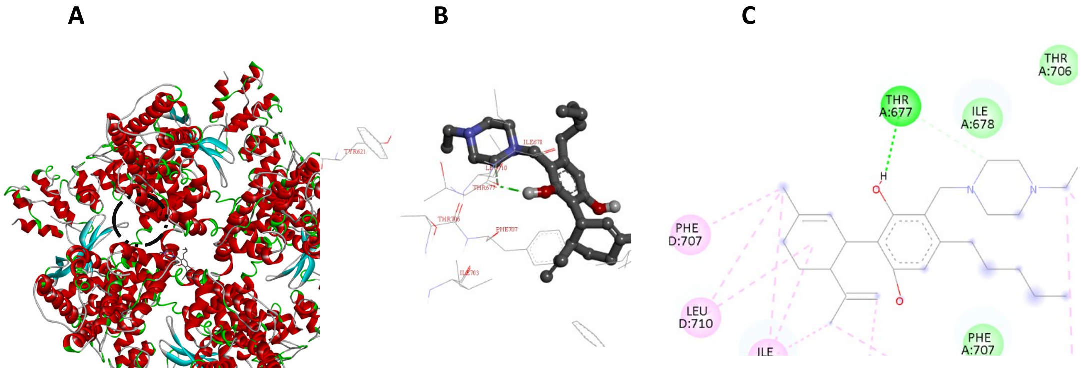

Figure S69 (k). Molecular docking interaction of **4i**

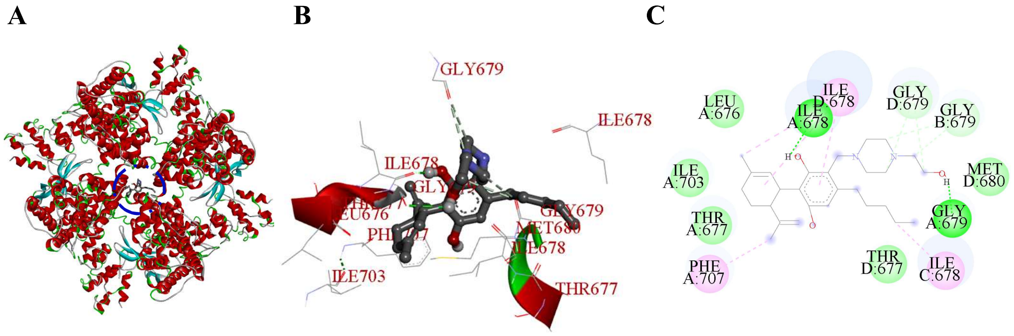

Figure S69 (l). Molecular docking interaction of **4j**

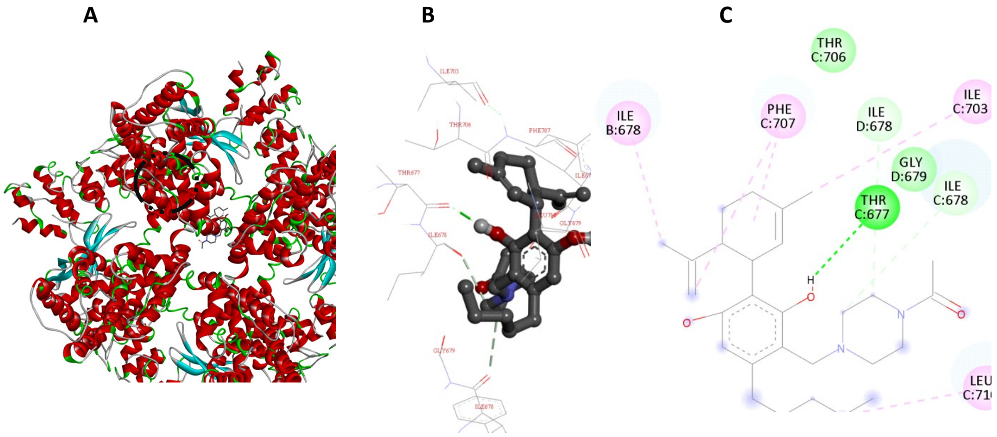

Figure S69 (m). Molecular docking interaction of **4k**

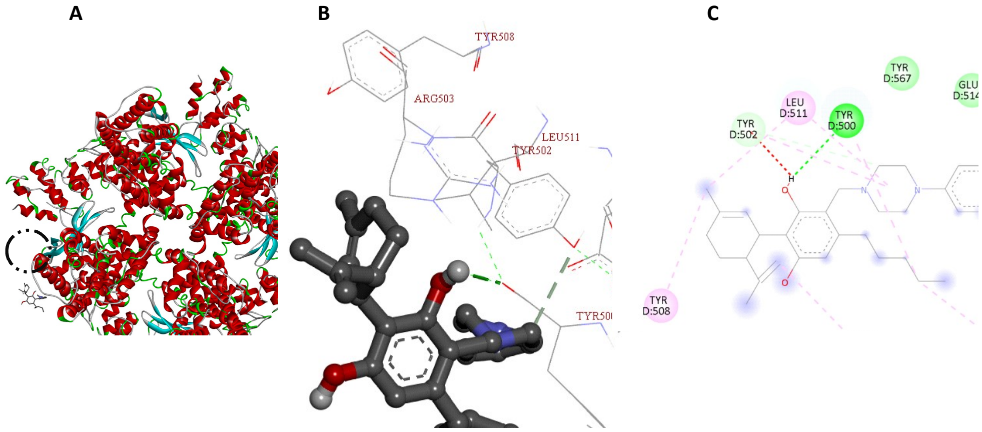

Figure S69 (n). Molecular docking interaction of **4l**

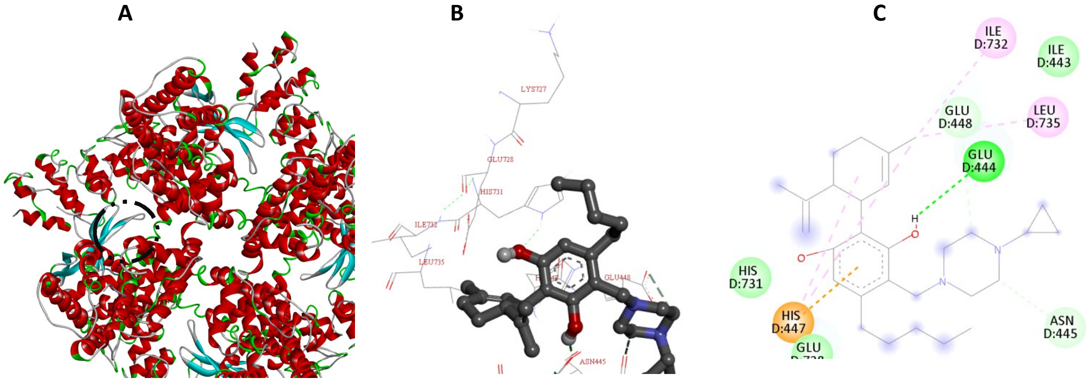

Figure S69 (o). Molecular docking interaction of **4m**

A B C

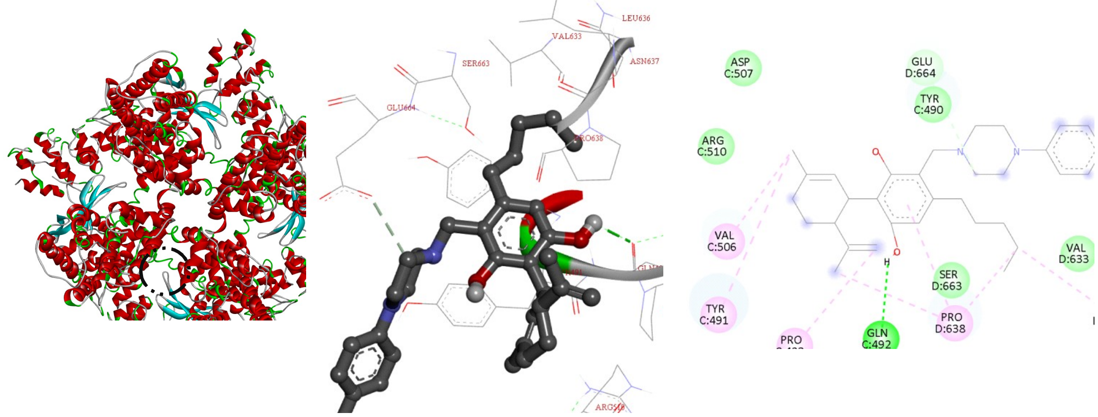

Figure S69 (p). Molecular docking interaction of **4n**

Figure S69. Molecular modelling studies

A. Ribbon representation of the molecular docked complex of TRPV4 (8T1F) protein with synthesized compounds (**4a-4o**) and **CBD-1** in space fill model.

B. 3D Binding pose of **4a-4o** and **CBD-1** with the residues of TRPV4 (8T1F) protein within 1 Å. Ligands are represented by ball and stick model and lines represent the residues of the protein.

C. 2D Binding interactions of **4a-4o** and **CBD-1** in the binding cavity of TRPV4 (8T1F) protein. Green colored dashed lines represent the hydrogen bonding interactions, the lavender colored dashed lines represent the π-π interactions and rest of residues show van der waals interactions.

Table S3. Binding Affinities of CS-85 and CBD 1 with TRPV4 (8T1F)

Compounds	Binding Energies	
	Antagonistic	Agonistic
**CBD 1**	-	–5.83
**4a**	-	0.01
**4a'**	–3.87	-
**4b**		-3.11
**4c**	-	-6.93
**4d**	–5.78	-
**4e**	-	–4.92
**4f**	-	–3.79
**4g**	-	–5.77
**4h**	-	–6.39
**4i**	–4.13	-
**4j**	–6.75	-
**4k**	–6.12	-
**4l**	-	–3.75
**4m**	-	–4.97
**4n**	-	–5.73
**4o**	-	3.33

Binding affinities of the **4(a–j)** and CBD **1** compounds with TRPV4 (8T1F) protein. Among the docked compounds 4a1, 4d, 4i, 4j and 4k bonded within the antagonistic binding site of the protein, **4j** exhibited the strongest binding affinity with the binding score of –6.75 kcal/mol.

Solubility studies of 4a, 4d, 4j (CS-85), 4k and CBD-1 *(in vitro)*

4a, **4d**, **4j** (**CS-85**) and **4k** were evaluated for solubility in various biorelevant media, like water, simulated gastric fluid (SGF) and simulated intestinal fluid (SIF) as compared to parent CBD **1**. Piperine was taken as a standard. The solubility studies were conducted following a previously reported procedure.[1, 2,3]

Table S4. Solubility in various biorelevant media

Solubility (µg/ml)				
**Entry**	**Compound**	**Water**	**SGF**	**SIF**
**1.**	**CBD-1**	<5	<5	<5
**2.**	**4a** (**CS-16**)	40<br>(>8 ↑)	>1500<br>(>300 ↑)	>1500<br>(> 300 ↑)
**3.**	**4d** (**CS-73**)	<5	>1500<br>(>300 fold ↑)	40<br>(8- fold ↑)
**4.**	**4j** (**CS-85**)	<5	>1500<br>(>300 fold ↑)	80<br>(16- fold ↑)
**5.**	**4k** (**CS-79**)	<5	>1500<br>(>300 fold ↑)	120<br>(24- fold ↑)
**6.**	Standard<br>(**Piperine**)	40	40	60

Table S5. Cell viability evaluation A375

S.No	Compound	Concentration	% cell viability
**1.**	**4a**	10μM	81.96±2.64
		5μM	90.41±0.78
**2.**	**4a'**	10μM	89.26±3.27
		5μM	94.87±2.66
**3.**	**4b**	10μM	73.7±5.21
		5μM	86.09±1.20
**4.**	**4c**	10μM	85.16±2.97
		5μM	90.69±0.74
**5.**	**4d**	10μM	92.24±1.87
		5μM	95.26±1.08
**6.**	**4e**	10μM	91.13±3.70
		5μM	94.12±1.81
**7.**	**4f**	10μM	90.53±3.46
		5μM	94.86±2.79
**8.**	**4g**	10μM	91.19±1.05
		5μM	95.29±2.24
**9.**	**4h**	10μM	94.42±0.88
		5μM	96.11±0.639
**10.**	**4i**	10μM	89.09±4.92
		5μM	93.52±1.72
**11.**	**4j** (**CS-85**)	10μM	94.26±1.57
		5μM	96.16±0.99
**12.**	**4k**	10μM	85.09±5.53
		5μM	94.76±1.13
**13.**	**4l**	10μM	92.68±2.15
		5μM	96.33±3.12
**14.**	**4m**	10μM	93.99±2.72
		5μM	96.71±1.67
**15.**	**4n**	10μM	91.37±2.49
		5μM	96.53±1.36
**16.**	**4o**	10μM	92.06±3.61
		5μM	94.86±1.21
**17.**	**CBD 1**	10μM	85.52±1.94
		5μM	93.24+ 0.327
**18.**	**Control**	-	100

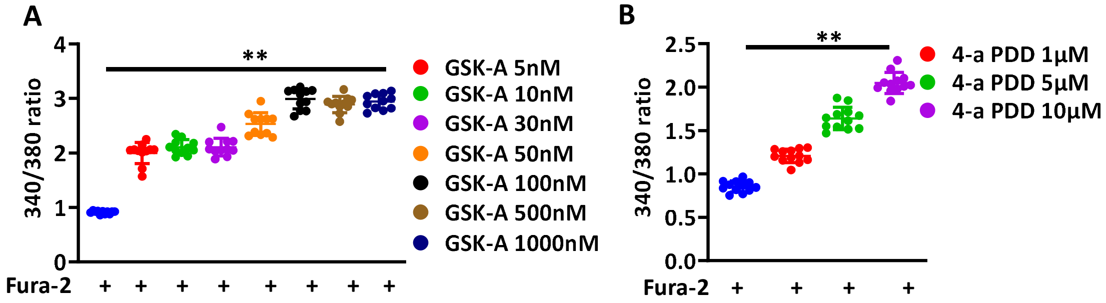

Figure S70 A,B. Concentration dependndent effect of TRPV4 agonists on Ca^{2+} influx: A375 cells were loaded with Fura-2 AM dye for 40 min followed by washing and treatment with GSK-A **(A)** or 4α-PDD **(B)** at indicated concentrations for 15 min. The cells were washed and fluorescence intensity measured at 340/510 and 380/510. Data are presented as twelve individual points from three independently performed experiments (n=3). Related to Figure 2. **<p0.001, compared to either GSK-A or 4α-PDD treated cells .

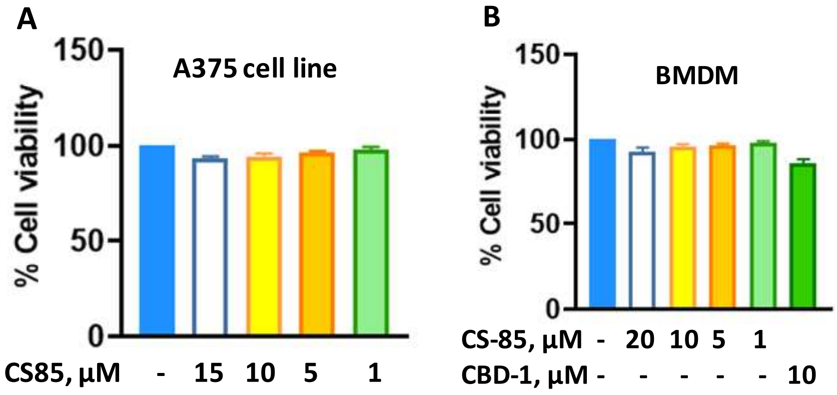

Figure S71 A,B. Cell Viability assays: Cell viability was evaluated by MTT assay. A375 cells **(A),** BMDMs **(B)** were treated with different concentration of CS-85 and/or CBD-1 as indicated, for 24 hour. Data are represented as mean ± SD of three independent experiments.

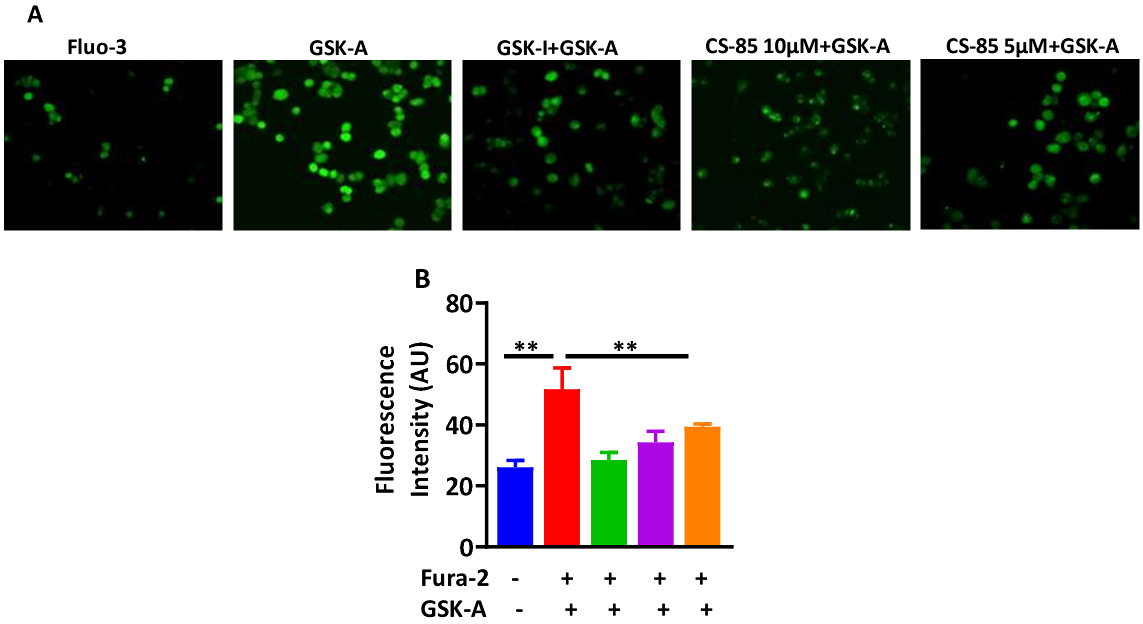

Figure S72 A,B. Effcet of CS-85 on TRPV4 mediated Ca^{2+} influx: A375 cells were loaded with Fluo-3 AM (7µM) for 40 min and washed. The cells were then treated with CS-85 for 15 min, followed by treatment with GSK-A (100nM) for additional 15 min. The images were taken using confocal microscope, 40 X Objective lens (CQ1 Confocal Imaging Cytometer, Yokogawa, Japan). Representative images of three experiments are shown **(A).** The histogram shows the quantitative analysis of images, performend on Image J software from three independently performed experiments **(B)**, (n=3). **<p0.001, compared to GSK-A treated cells .

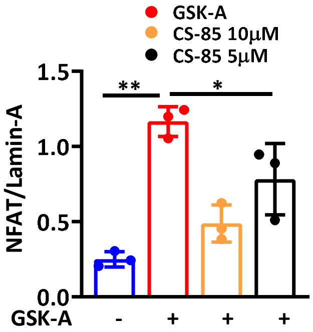

Figure S73. Densitometry analysis: BMDMs were stimulated with indicated concentrations of CS-85 for 30 min, followed by treatment with GSK-A (100nM) for 4h. The cytosolic fraction of cells was prepared using fractionation buffer. The nuclear fraction of NFAT1 was extracted by using RIPA lysis buffer, and analyzed by immunoblotting. Graph shows the

densitometry analysis of blots of three experiments of Figure 2K (n=3). **<p0.001, *<p0.05 compared to GSK-A treated cells .

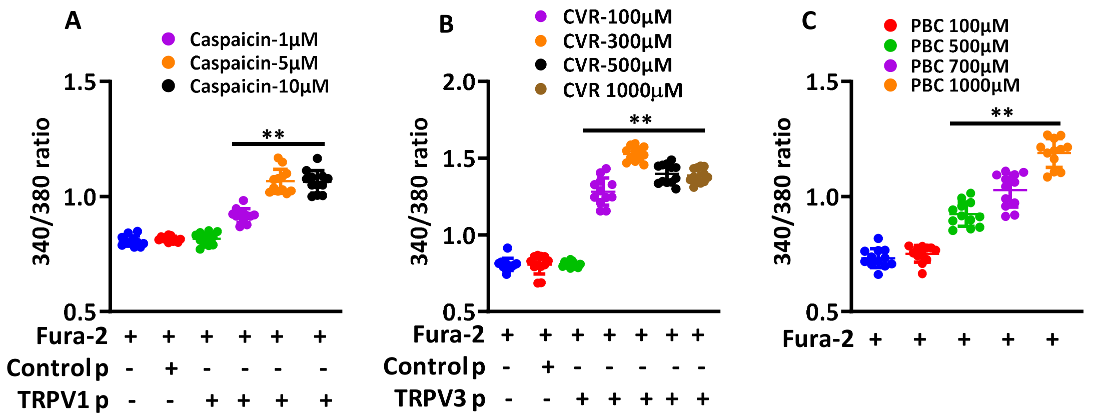

Figure S74. A,B. Calcium influx via TRPV channels: RAW 264.7 cells were transfected with either hTRPV1 or hTRPV3 plasmid DNA. 24 after transfection, the cells were loaded with Fura-2 AM dye. 40 min later, the cells were washed and treated with CS-85 at indicated concentrations for 15 mins. The cells were either incubated with Capsaicin (5μM) for 12 min at 37^{0}C for TRPV1 activation **(A)**, or Carvacrol (300μM) for 12 min at RT for TRPV3 activation **(B).** The cells were washed and and the fluorescence intensity was measured at 340/510 and 380/510. Data are presented as twelve individual points from three independently performed experiments (n=3).

C. HCT116 cells were loaded with Fura-2 AM dye. 40 min later, the cells were washed and treated with CS-85 for 15 mins followed by probenecid (1mM) treatment for next for 9 min at RT. The cells were washed and the fluorescence was measured at 340/510 and 380/510. Data are presented as twelve individual points from three independently performed experiments (n=3). Related to Figure 2. **<p0.001 compared to control cells . Related to Figure 2L,M and N.

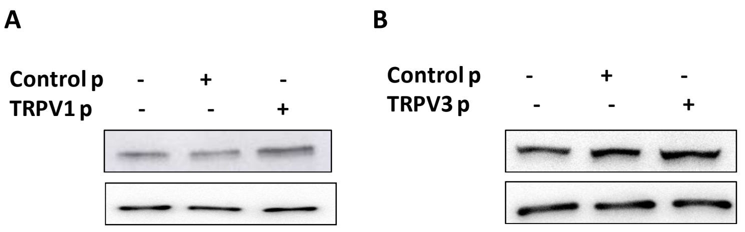

Figure S75 A, B.Transfection of hTRPV1 and hTRPV3 in RAW 264.7 cells. The cells were transfected with either hTRPV1 or hTRPV3 plasmid DNA for confirmation of transfection success of these plasmids. 24-hour post transfection, the cell lysates were prepared and subjected to immunoblotting. β-actin was used as an internal control (n=3). Realted to Figures 2L, M.

Table S6. Cell viability evaluation RAW 264.7

S.No	Compound	Concentration	% cell viability
**1.**	**4a**	10μM	83.51±0.67
		5μM	93.18±1.15
**2.**	**4a'**	10μM	92.46±1.29
		5μM	94.44±0.92
**3.**	**4b**	10μM	61.72±8.12
		5μM	65.99±6.5
**4.**	**4c**	10μM	89.9±1.25
		5μM	92.80±0.94
**5.**	**4d**	10μM	90.68±0.74
		5μM	93.86±1.14
**6.**	**4e**	10μM	90.40±0.95
		5μM	94.43±1.36
**7.**	**4f**	10μM	92.06±1.19
		5μM	94.14±1.82
**8.**	**4g**	10μM	90.18±1.31
		5μM	93.05±1.71
**9.**	**4h**	10μM	87.12±1.96
		5μM	94.72±2.74
**10.**	**4i**	10μM	87.64±0.18
		5μM	94.23±2.1
**11.**	**4j (CS-85)**	10μM	95.05±1.91
		10μM	97.31±0.73
**12.**	**4k**	10μM	91.62±0.25
		5μM	95.35±0.43
**13.**	**4l**	10μM	92.78±0.56
		5μM	94.58±1.08
**14.**	**4m**	10μM	89.34±2.08
		5μM	93.97±4.35
**15.**	**4n**	10μM	89.85±1.15
		5μM	95.21±0.73
**16.**	**4o**	10μM	89.92±0.94
		5μM	93.40±1.16
**17.**	**CBD 1**	10μM	91.0±1.64
		5μM	95.55±1.55
**18.**	**Basal Control**	-	100

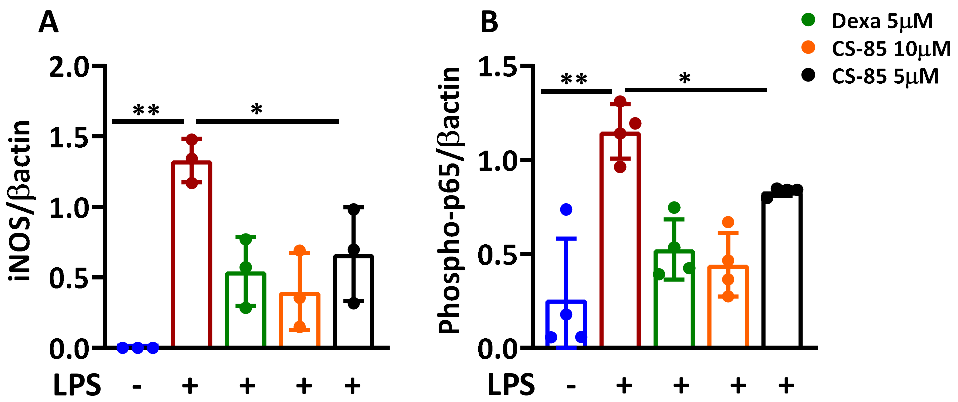

Figure S76 A,B. Densitometry analysis: BMDMs were treated with CS-85 or dexamethasone for 1h, followed by LPS stimulation for 24 h. The cell lysates were analyzed for phospho-p65 and iNOS by immunoblotting. Graphs show the densitometric analysis of blots of iNOS **(A)** and phospho-p65 **(B).** **<p0.001, *<p0.05 compared to LPS treated cells . Related to Figure 3H.

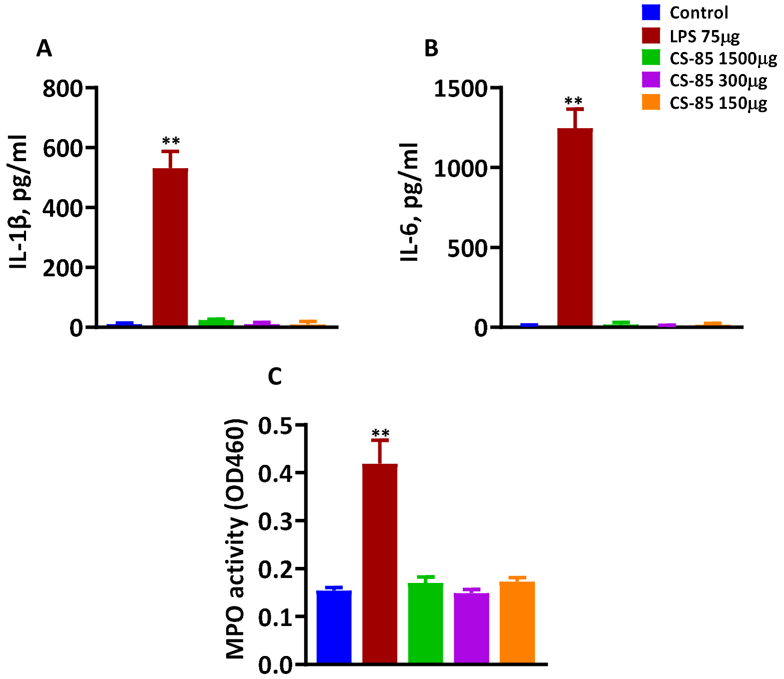

Figure S77 A-C. Pulmonary toxicity of CS-85. Different groups of mice received varying doses of CS-85 (30, 300 and 1500μg) administered endotracheally. Post 24 hour of CS-85 administration, BAL was collected from these mice and used for estimation of cytokines IL-1β **(A)** and IL-6 **(B).** Also, MPO activity assay was performd on lung homogenate samples of these mice **(C).** **<p0.001 compared to control mice.

References

1. Cham, P. S.; Deepika; Bhat, R.; Raina, D.; Manhas, D.; Kotwal, P.; Mindala, D. P.; Pandey, N.; Ghosh, A.; Saran, S.; Nandi, U.; Khan, I. A.; Singh, P. P., Exploring the Antibacterial Potential of Semisynthetic Phytocannabinoid: Tetrahydrocannabidiol (THCBD) as a Potential Antibacterial Agent against Sensitive and Resistant Strains of Staphylococcus aureus. *ACS Infectious Diseases* **2024,** *10* (1), 64-78.
2. Cham, P. S.; Kotwal, P.; Sharma, K.; Dhiman, S.; Singh, L.; Singh, V. P.; Kumar, A.; Nandi, U.; Singh, P. P., Cannabidiol-Based Prodrugs: Synthesis and Bioevaluation. *ACS Medicinal Chemistry Letters* **2024,** *15* (2), 221-229.
3. Cham, P. S.; Singh, A.; Jamwal, A.; Singh, R.; Anand, R.; Manhas, D.; Sharma, S.; Singh, V. P.; Nandi, U.; Singh, S. K.; Singh, P. P., Discovery of Ring-Annulated Analogues of Cannabidiol as Potential Anticancer Agents: Synthesis and Biological Evaluation. *ACS Medicinal Chemistry Letters* **2024,** *15* (11), 1832-1842.
